# Expanding the antiprotozoal activity and the mechanism of action of n-butyl and iso-butyl ester of quinoxaline-1,4-di-*N*-oxide derivatives against *Giardia lamblia*, *Trichomonas vaginalis*, and *Entamoeba histolytica.* An *in vitro* and *in silico* approach

**DOI:** 10.1080/14756366.2024.2413018

**Published:** 2024-10-29

**Authors:** Alonzo González-González, Oscar Sánchez-Sánchez, Lilián Yépez-Mulia, Timoteo Delgado-Maldonado, Lenci K. Vázquez-Jiménez, Gabriel López-Velázquez, José Ignacio de la Mora-de la Mora, Sebastian Pacheco-Gutierrez, Laura Chino-Ríos, Diego Arias, Adriana Moreno-Rodríguez, Alma Paz-González, Eyra Ortíz-Pérez, Gildardo Rivera

**Affiliations:** a Laboratorio de Biotecnología Farmacéutica, Centro de Biotecnología Genómica, Instituto Politécnico Nacional, Reynosa, México; b Unidad de Investigación Médica en Enfermedades Infecciosas y Parasitarias, Hospital de Pediatría, Instituto Mexicano del Seguro Social, México, México City; c Laboratorio de Biomoléculas y Salud Infantil, Instituto Nacional de Pediatría, México, México City; d Laboratorio de Enzimología Molecular, Instituto de Agrobiotecnología del Litoral (CONICET-UNL), Santa Fe, Argentina; e Facultad de Bioquímica y Ciencias Biológicas, Universidad Nacional del Litoral, Santa Fe, Argentina; f Laboratorio de Estudios Epidemiológicos, Clínicos, Diseños Experimentales e Investigación, Facultad de Ciencias Químicas, Universidad Autónoma “Benito Juárez” de Oaxaca

**Keywords:** Quinoxaline-1,4-di-*N*-oxide, antiprotozoal activity, *Giardia lamblia*, *Trichomonas vaginalis*, *Entamoeba histolytica*

## Abstract

In this study, n-butyl and iso-butyl quinoxaline-7-carboxylate-1,4-di-*N*-oxide derivatives were evaluated *in vitro* against *Giardia lamblia* (*G. lamblia*)*, Trichomonas vaginalis* (*T. vaginalis*), and *Entamoeba histolytica* (*E. histolytica*). The potential mechanism of action determination was approached by *in silico* analysis on *G. lamblia* and *T. vaginalis* triosephosphate isomerase (*Gl*TIM and *Tv*TIM, respectively), and on *E. histolytica* thioredoxin reductase (*EhTrxR*). Enzyme inactivation assays were performed on recombinant G*l*TIM and *Eh*TrxR. Compound T-167 showed the best giardicidal activity (IC_50_ = 25.53 nM) and the highest inactivation efficiency against G*l*TIM without significantly perturbing its human homolog. Compounds T-142 and T-143 showed the best amoebicidal (IC_50_ = 9.20 nM) and trichomonacidal (IC_50_ = 45.20 nM) activity, respectively. Additionally, T-143 had a high activity as giardicial (IC_50_ = 29.13 nM) and amoebicidal (IC_50_ = 15.14 nM), proposing it as a broad-spectrum antiparasitic agent. Compounds T-145, and T-161 were the best *Eh*TrxR inhibitors with IC_50_ of 16 µM, and 18 µM, respectively.

## Graphical Abstract

**Figure:**
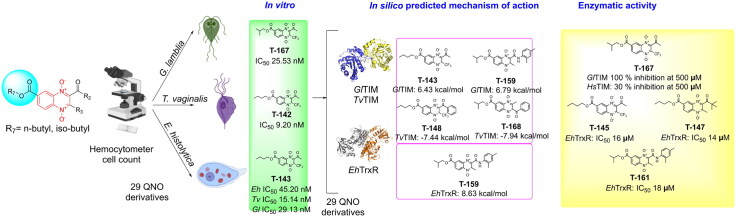

## Introduction

Parasitic diseases, particularly those caused by protozoa, constitute a significant global health concern, impacting more than one billion individuals worldwide, with a pronounced prevalence among populations in low-income countries. Among these diseases, American trypanosomiasis, leishmaniasis, giardiasis, amoebiasis, and trichomoniasis are included^1^. While the first two bear a commonality in that they are classified as Neglected Tropical Diseases (NTDs) and are commonly studied together, the last three are customarily grouped considering their carbohydrate metabolism and that they lack mitochondria^2^.

Giardiasis is one notable protozoan disease caused by *Giardia lamblia* (*G. lamblia*), responsible for approximately 280 million cases of diarrhoea annually in humans^3–4^. The symptoms of giardiasis encompass diarrhoea, abdominal bloating, cramps, malabsorption, and weight loss^3^^,^^5^. It has been associated with intestinal dysbiosis and the subsequent development of chronic post-infection inflammatory bowel syndrome^6^. The pharmacological treatment options for giardiasis involves nitroimidazoles (such as metronidazole, tinidazole, secnidazole, and ornidazole), benzimidazoles (albendazole, and mebendazole), nitazoxanide, furazolidone, quinacrine, chloroquine, and paromomycin. These treatments exhibit varying levels of effectiveness, ranging from 40% to 90%. However, these treatments are reported to have important side effects, including pancreatitis, diarrhoea, and even neuropathies^7^.

Trichomoniasis is the disease caused by *Trichomonas vaginalis* (*T. vaginalis*), which is one of the two most common women’s sexually transmitted infection^2^. A total of 270 million cases of trichomoniasis are reported annually^8^. The current treatment for trichomoniasis is using nitroimidazoles such as metronidazole and tinidazole. However, these drugs have severe side effects, including gastritis, nausea, vomiting, diarrhoea, seizures, and difficulty breathing, often leading to treatment abandonment^9^, as well as the development of parasite resistance to these drugs^10^.

Amoebiasis is another relevant protozoan disease caused by *Entamoeba histolytica* (*E. histolytica*), the second leading cause of death by parasitic diseases, recording 40,000 to 100,000 deaths annually^11^. The infection by *E. histolytica* may result in amoebic colitis and hepatic abscesses, though in some cases the infection is asymptomatic^9^. Nitroimidazoles such as metronidazole, tinidazole, secnidazole, and ornidazole are the current treatment for amoebiasis; among these, metronidazole is the most common, for its availability and low cost, and secnidazole, and their use holds the same challenges already described.

In common, these parasitic diseases (giardiasis, amoebiasis, and trichomoniasis) share the use of metronidazole, as the first line of treatment^2^^,^^8^^,^^12^; however, failed treatment and parasite resistance have been reported^13–15^. Besides, this drug causes genotoxicity in human cells, mutagenicity in bacteria, and carcinogenicity in rodents^9^. Additionally, metronidazole has been related to potential carcinogenic, teratogenic, and embryogenic effects with long treatment use or high doses^16^. The long treatment use has been linked to peripheral neuropathy, convulsions, and cerebellar ataxia^17^. This data makes it relevant to develop new and improved treatments with higher efficacy and a lowered or null toxicity towards humans.

On the other hand, quinoxaline-1,4-di-*N*-oxide (QNO) is a scaffold to develop new bactericidal, antimycobacterial, antitumoral, fungicidal, antiparasitic, anti-inflammatory, and antioxidant agents^18–23^. In particular, QNOs have trichomonacidal^24^, trypanocidal^25^^,^^26^, and leishmanicidal activity^27^^,^^28^. In the last decade, our research group has followed a systematic approach to determine the biological effect of the introduction of short-chain ester substitutions (methyl, ethyl, n-propyl, and iso-propyl, and n-butyl and iso-butyl) at 7-position on the quinoxaline 1,4-*N*-oxide ring, resulting in a series of compounds (7EQNOs) that exhibit noteworthy trypanocidal and leishmanicidal effects achieving efficacy within the low micromolar range, with comparable or better activity than reference drugs^29–32^.

Similarly, comparable successes regarding giardicidal, trichomonacidal, and amoebicidal activities have been reported for methyl, ethyl, n-propyl, and iso-propyl derivatives of esters of QNO, with half-maximal inhibitory concentration (IC_50_) values better than reference drug metronidazole^20^^,^^33–35^. These results emphasise their potential as broad-spectrum antiparasitic agents. In recent studies, the mode of action of QNOs revealed that these types of compounds act through the inhibition of important parasitic proteins, such as *T. vaginalis* triosephosphate isomerase (*Tv*TIM), as it plays an important role in efficient energy production since it is part of the glycolytic pathway^35^, and *E. histolytica* thioredoxin reductase (*Eh*TrxR) which is important for the redox homeostasis of the parasite^34^. Thus, these types of compounds may act selectively against parasitic cells and have low to null effects against human cells.

Considering the need for alternative, more effective giardicidal, trichomonacidal, and amoebicidal agents, and the favourable reported effect that QNO has for one-, two- and three-carbon esters at 7-position, in this study, the antiparasitic effect of extending to a four-carbon ester at 7-position in the newly synthesised n-butyl and iso-butyl esters of QNO was explored. The potential inhibitory effects that these derivatives may have on the drug targets, *Gl*TIM and *Tv*TIM, as well as on the *Eh*TrxR, was analyzed through molecular docking studies. Finally, the inhibitory effects of some of the best derivatives on the enzyme activity of G*l*TIM and *Eh*TrxR were evaluated.

## Material and methods

### N-butyl and iso-butyl quinoxaline-7-carboxylate-1,4-di-N-oxide derivatives

The *n*-butyl and iso-butyl quinoxaline-7-carboxylate-1,4-di-*N*-oxide derivatives (T-137 to T-170) were synthesised using the Beirut reaction as described by Gomez-Caro *et al.* in 2011^36^, and were structurally elucidated by NMR, FT-IR, and UPLC-MS analysis^32^.

### Biological evaluation

*G. lamblia* WB strain, *T. vaginalis* GT3 strain, and *E. histolytica* strain HM1-IMSS were used in all experiments, all parasitic strains were obtained from the Unidad de Investigación Médica en Enfermedades Infecciosas y Parasitarias, Hospital de Pediatría, Instituto Mexicano del Seguro Social. *In vitro* susceptibility assays to determine IC_50_ values were performed using a method previously described^37^. For this, 4 × 10^4^ *G. lamblia*; 6 × 10^3^ *E. histolytica,* or 4 × 10^4^ *T. vaginalis* trophozoites were incubated for 48 h at 37 °C with different concentrations of quinoxaline derivatives T-137 to T-170 (0.5 ng/mL to 10 μg/mL) using dimethyl sulfoxide (DMSO) (0.05%) as solvent. Metronidazole and albendazole were used as positive controls, and parasites in the medium without drugs containing DMSO (0.05%) were included as negative controls. At the end of the incubation period, trophozoites were washed and subcultured for another 48 h in a fresh medium without drugs. Then, trophozoites were counted with a haemocytometer and the IC_50_ was calculated by Probit analysis. Experiments were carried out in triplicate and repeated at least twice.

### Cytotoxic evaluation

The murine macrophage cell line J774.2 was maintained in culture flasks with RPMI 1640 medium supplemented with 10% FBS, and 100 U/mL antibiotic-antifungal mixture (Gibco). Cells were incubated at 37 °C with 5% CO_2_ and humidity. Murine macrophage cell line J774.2 is maintained at Laboratorio de Estudios Epidemiológicos, Clínicos, Diseños Experimentales e Investigación, Facultad de Ciencias Químicas, Universidad Autónoma “Benito Juárez” de Oaxaca. Macrophages were washed and viability was assessed by the MTT colorimetric assay. In a 96-well microplate, 1 × 10^5^ macrophages were added per well and a dose-response assay was performed. Compounds were evaluated using serial dilutions to determine half-maximal cytotoxic concentration (CC_50_) values in triplicate considering 0.2% DMSO as negative control.

### Molecular docking analysis

A molecular docking analysis was conducted to evaluate the possible interaction of all n-butyl and iso-butyl quinoxaline-7-carboxylate-1,4-di-*N*-oxide derivatives against three potential enzymatic targets: *Gl*TIM, *Tv*TIM, and *Eh*TrxR. The ligand structures were drawn with Marvin Sketch 21.13, and energy was minimised with OpenBabel software, saved in pdb format. The protein receptor TIM from *G. lamblia,* was obtained from the AlphaFold database from the Uniprot access code P36186, which is the unmutated model of PDB:2DP3; TIM from *T. vaginalis*, and TrxR from *E. histolytica* structures were obtained from the Protein Data Bank (PDB), access codes PDB:3QSP and 4A5L, respectively. Water molecules, co-crystalised ligands, and ions were removed from the protein structures, and they were then energy minimised using the Yasara Energy Minimization Server before docking simulation, protein structures were analysed to confirm that minimization did not alter catalytic site residue orientation significantly, Supplementary Figures S1–S3 show overlapping structures. All docking simulations were performed with gnina software v1.0.3^38^.

The TIM structures were 3D aligned using PyMol software, and molecular docking analysis for both was done on the catalytic site coordinate space (X= −8.704, Y = 31.659, and Z= −14.097) using 24 Å in each axis with 1 Å spacing in the Grid Box, additionally for *Gl*TIM two other docking sites were explored, interface, and a site near Cys222 considering same Grid Box dimensions. For TrxR, a blind docking was carried out in SwissDock server, and the two highest populated clusters were considered for two docking sites encompassing redox-active site and NADPH-binding site (X= −8.26, Y= −19.502, and Z= −4.903, and X = 7.292, Y= −18.324, and Z= −11.892, respectively) using 24 Å in each axis with 1 Å spacing in the Grid Box. After to assess specificity towards parasitic TIM, molecular docking was conducted in human TIM (PDB: 4POC) considering the same conditions and docking space as parasitic proteins. The results were analysed considering the lowest binding free energy (BFE) for each protein–ligand complex, which were later analysed to determine the molecular interactions between protein and ligand utilising the Protein–Ligand Interaction Profiler (PLIP) software^39^.

### Inhibition of GlTIM and HsTIM enzyme activity

*Escherichia coli* BL21(DE3) pLys bacteria containing the plasmid pET3a-HisTEV with the gene coding for *Gl*TIM wild type were grown in LB medium supplemented with 0.1 mg/mL ampicillin and incubated at 37 °C^40^. When the cultures reached Abs_600_ = 0.8, they were induced to express the recombinant enzyme using 0.4 mM IPTG and incubated overnight at 30 °C with shaking at 180 rpm. After induction and growth, the bacteria were collected by centrifugation (6500 rpm, 15 min) and suspended in 40 mL of lysis buffer (pH 8.0) containing 50 mM Tris, 50 mM NaCl, 5 mM β-mercaptoethanol, and 1 mM phenylmethylsulfonyl fluoride (PMSF). The bacterial suspension was disrupted by sonication and centrifuged at 9000 rpm for 1 h, at 4 °C. Protein purification was performed via IMAC using a Profinity Ni^2+^ charged resin previously equilibrated with lysis buffer. The soluble protein fraction was mixed with the equilibrated Ni^2+^ charged resin and incubated at room temperature with shaking for 30 min. Then, the column was washed with the same buffer (10 column volumes) to remove proteins without the His-tag sequence. The desired proteins were eluted with lysis buffer containing 200 mM imidazole and adjusted to pH 8.0. The purified protein was concentrated using Amicon ultrafiltration units. The purity of the enzymes was analysed via sodium dodecyl sulfate-polyacrylamide gel electrophoresis (16% SDS–PAGE gel) and stained with colloidal Coomassie Brilliant Blue. The enzyme concentration was spectrophotometrically determined (Spectrophotometer Cary 50, Varian Inc.) at 280 nm using the extinction coefficient ε_280_ = 26,600 M^−1 ^cm^−1^.

Expression of human TIM (*Hs*TIM) was performed by using the *E. coli* BL21-CodonPlus (DE3)-RIL strain (Stratagene) containing the plasmid pET3a-HisTEV with the gene coding for *Hs*TIM. Expression and purification steps were as above-mentioned for *Gl*TIM. The enzyme concentration was spectrophotometrically determined (Spectrophotometer Cary 50, Varian Inc.) at 280 nm using the extinction coefficient ε_280_ = 33,460 M^−1 ^cm^−1^.

For the inhibition assays, freshly purified recombinant *Gl*TIM was incubated at 0.2 mg/mL for 2 h at 37 °C in the presence of 500 μM of each n-butyl and iso-butyl quinoxaline-7-carboxylate-1,4-di-*N*-oxide derivatives. After incubation, the samples were diluted, and 5 ng/mL were taken to measure their enzymatic activity. Enzyme activity was spectrophotometrically measured (Spectrophotometer Cary 50, Agilent Technologies, CA, USA) by following DHAP synthesis with a coupled system that followed the oxidation of NADH at 340 nm^41^. Results are expressed as the percent activity versus each compound at 500 μM, with the enzyme activity without any drug set as 100%. Additionally, *Gl*TIM and *Hs*TIM (separately) were incubated at 0.2 mg/mL for 2 h at 37 °C in the presence of 5, 10, 50, 150, 250, 350, and 500 μM of the **T-167** derivative. Results are expressed as the percent activity versus **T-167** concentration. All results represent the arithmetic mean of at least four independent experiments.

### Inhibition of EhTrxR

Recombinant *E. histolytica* thioredoxin reductase (*Eh*TRXR) was expressed in *E. coli* BL21 (DE3) as His-tag (N-terminal) recombinant proteins using pET28a vector, and it was purified using IMAC as previously described^42^. 5,5′-Dithiobis(2-nitrobenzoic acid) (DTNB) reductase activity was measured by monitoring the production of thionitrobenzoate at 405 nm in a reaction mixture comprising 50 mM potassium phosphate, pH 7.0, 2 mM EDTA, 300 µM NADPH, 5 mM DTNB, and 0.1 µM *Eh*TRXR. Activity was calculated using the molar extinction coefficient at 405 nm of 13.6 mM^−1^cm^− 1^ and considering that 1 mol of NADPH yields 2 mol of thionitrobenzoate^42^. The enzyme assays were performed at 30 °C, in a final volume of 50 μL, and using a Multiskan Ascent one-channel vertical light path filter photometer (Thermo Electron Co.). All results represent the arithmetic mean of at three independent experiments.

## Results and discussion

Twenty-eight n-butyl and iso-butyl quinoxaline-7-carboxylate 1,4-di-*N*-oxide derivatives were synthesised through the Beirut reaction. All compounds were characterised by infra-red (IR), proton and carbon nuclear magnetic resonance (^1^H-NMR and ^13^C-NMR), and ultra-performance liquid chromatography-mass spectrometry (UPLC-MS) for further biological evaluation (See Supplementary Material).

### Biological activity

Table 1 summarises the IC_50_ values determined for n-butyl and iso-butyl quinoxaline series against *G. lamblia*, *T. vaginalis*, and *E. histolytica*. Albendazole and metronidazole were used as reference drugs.

**Table 1.** Antiparasitic activity (IC_50_) of *n*-butyl and iso-butyl quinoxaline-7-carboxylate-1,4-di-*N*-oxide derivatives against *G. lamblia, T. vaginalis,* and *E. histolytica.*

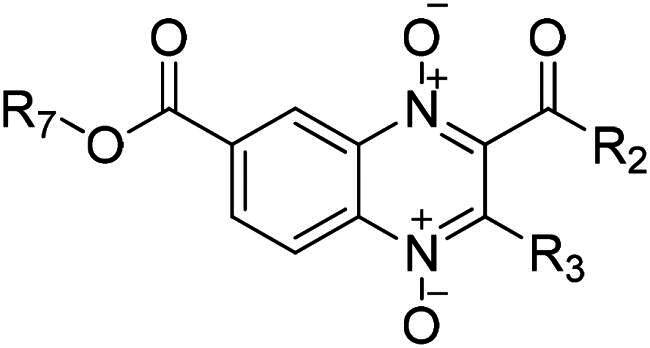							
Code	R_2_	R_3_	R_7_	*G. lamblia* (IC_50_ nM)	*T. vaginalis* (IC_50_ nM)	*E. histolytica* (IC_50_ nM)	Cytotoxicity (Macrophages J774.2) (nM)
T-137	-CH_3_	-CH_3_	CH_3_(CH_2_)_3_-	77.01 ± 9.43	257.76 ± 6.29	83.3 ± 1.57	58390 ± 1125
T-138	-OCH_3_	-CH_3_	CH_3_(CH_2_)_3_-	48.48 ± 1.50	88.29 ± 11.98	40.4 ± 4.49	46240 ± 3263
T-139	-OCH_2_CH_3_	-CH_3_	CH_3_(CH_2_)_3_-	47.39 ± 5.75	103.41 ± 2.87	34.47 ± 7.18	> 200000
T-140	-OC(CH_3_)_3_	-CH_3_	CH_3_(CH_2_)_3_-	49.18 ± 2.66	101.03 ± 3.99	29.24 ± 2.66	> 200000
T-141	-OCH_2_ C_6_H_5_	-CH_3_	CH_3_(CH_2_)_3_-	70.70 ± 1.22	165.79 ± 6.10	40.22 ± 2.44	>200000
T-142	-C_6_H_5_	-CH_3_	CH_3_(CH_2_)_3_-	36.82 ± 0.66	81.54 ± 3.95	9.2 ± 1.32	17650 ± 2143
T-143	-HN-*p*-C_6_H_4_Cl	-CH_3_	CH_3_(CH_2_)_3_-	29.13 ± 5.83	45.20 ± 6.99	15.14 ± 2.33	50280 ± 2801
T-144	-NH-[2,4(CH_3_)_2_C_6_H_3_]	-CH_3_	CH_3_(CH_2_)_3_-	46.07 ± 1.18	118.15 ± 4.73	47.26 ± 4.73	ND
T-145	-CH_3_	-CF_3_	CH_3_(CH_2_)_3_-	40.32 ± 6.72	107.50 ± 16.13	57.78 ± 6.72	> 200000
T-146	-OCH_2_CH_3_	-CF_3_	CH_3_(CH_2_)_3_-	26.11 ± 6.22	101.96 ± 2.49	64.66 ± 3.73	> 200000
T-148	-C_6_H_5_	-CF_3_	CH_3_(CH_2_)_3_-	39.16 ± 3.46	82.92 ± 8.06	16.12 ± 3.46	48370 ± 1648
T-149	-C_4_H_3_S	-CF_3_	CH_3_(CH_2_)_3_-	27.26 ± 2.27	97.71 ± 9.09	18.17 ± 2.27	ND
T-150	-C_10_H_7_	-CF_3_	CH_3_(CH_2_)_3_-	51.64 ± 2.27	123.93 ± 2.27	36.14 ± 1.14	> 200000
T-151	-NH-C_6_H_5_	-C_6_H_5_	CH_3_(CH_2_)_3_-	113.74 ± 4.38	153.11 ± 3.28	65.62 ± 2.19	44140 ± 3454
T-155	-CH_3_	-CH_3_	(CH_3_)_2_CHCH_2_-	157.17 ± 4.72	191.75 ± 6.29	58.16 ± 1.57	87220 ± 213
T-156	-OCH_3_	-CH_3_	(CH_3_)_2_CHCH_2_-	95.77 ± 4.49	248.41 ± 5.99	59.85 ± 5.99	ND
T-157	-C_6_H_5_	-CH_3_	(CH_3_)_2_CHCH_2_-	43.40 ± 1.32	152.57 ± 6.58	55.24 ± 5.26	34220 ± 3037
T-158	-NHC_6_H_5_	-CH_3_	(CH_3_)_2_CHCH_2_-	62.00 ± 1.27	164.49 ± 10.13	37.96 ± 2.53	> 200000
T-159	-NH-[2,4(CH_3_)_2_C_6_H_3_]	-CH_3_	(CH_3_)_2_CHCH_2_-	94.52 ± 3.54	105.15 ± 1.18	30.71 ± 5.91	> 200000
T-161	-HN-*p-*C_6_H_4_Cl	-CH_3_	(CH_3_)_2_CHCH_2_-	120.01 ± 1.16	137.49 ± 3.50	29.13 ± 3.50	ND
T-163	-C_6_H_5_	-CF_3_	(CH_3_)_2_CHCH_2_-	34.55 ± 2.30	100.20 ± 5.76	57.58 ± 2.30	ND
T-164	-C_4_H_3_S	-CF_3_	(CH_3_)_2_CHCH_2_-	106.80 ± 9.21	118.16 ± 10.53	27.26 ± 5.26	43850 ± 1890
T-165	-C_4_H_3_O	-CF_3_	(CH_3_)_2_CHCH_2_-	101.39 ± 2.36	124.97 ± 2.36	30.65 ± 2.36	81840 ± 3586
T-166	-C_10_H_7_	-CF_3_	(CH_3_)_2_CHCH_2_-	41.31 ± 2.07	82.62 ± 4.13	50.6 ± 5.17	ND
T-167	-CH_3_	-CF_3_	(CH_3_)_2_CHCH_2_-	25.53 ± 1.34	90.03 ± 6.72	64.5 ± 5.38	25400 ± 81.5
T-168	-CH_3_	-C_6_H_5_	(CH_3_)_2_CHCH_2_-	39.45 ± 2.63	149.94 ± 14.47	49.98 ± 2.63	28530 ± 1687
T-169	-NHC_6_H_5_	-C_6_H_5_	(CH_3_)_2_CHCH_2_-	98.43 ± 2.19	115.93 ± 5.47	43.74 ± 2.19	> 200000
T-170	-CH_3_	-NH-[2,4(CH_3_)_2_C_6_H_3_]	(CH_3_)_2_CHCH_2_-	108.70 ± 3.55	129.96 ± 8.27	33.08 ± 2.36	> 200000
Albendazole				48.99 ± 5.65	1827.844 ± 86.68	43981.31 ± 2468	
Metronidazole				1150.97 ± 40.90	210.3295 ± 8.76	251.2269 ± 5.48	

#### Giardicidal activity

Ten n-butyl derivatives (**T-138**, **T-139**, **T-142**-**T-146**, and **T-148**-**T-150**) had better giardicidal activity than reference drugs albendazole and metronidazole (IC_50_ values of 48.99, and 1150.97 nM, respectively). For n-butyl series, the effect of the aromatic ring size when comparing **T-148**, **T-149**, and **T-150** suggests that steric effect at 2-position is an important factor, as two-member ring **T-150** is the least active compound. From bio-isosteres phenyl and thienyl, the latter is most active suggesting that the addition of the sulphur atom favours biological activity. The introduction of an ester favours activity, as the change from ketone **T-137** to ester **T-138** leads to a 1.6-fold increase in activity, the length or size of the ester has a negligible effect, as **T-138**, **T-139**, and **T-140** have almost identical IC_50_ values. On the other hand, the nature of the ester plays a more noteworthy role, as the change from an aliphatic ester to an aromatic ester (**T-141**) causes a 1.4-fold decrease in giardicidal activity. The introduction of substituted benzamides at 2-position favours activity, where chloro-substituted on the phenyl ring is more active than dimethyl-substituted. The introduction of trifluoromethyl group at 3-position leads to an increased giardicidal activity.

For iso-butyl series, like n-butyl series, bulkier aromatic rings led to a decreased giardicidal activity when comparing **T-163**, and **T-166** (phenyl vs naphthyl) which is further emphasised by comparing with **T-167** introducing a methyl group instead of a bulkier aromatic ring, being the most giardicidal compound. Unlike n-butyl, introducing heterocycles at 2-position leads to a 3.1-fold decrease going from phenyl to thienyl or furyl (**T-163** vs. **T-164** and **T-165**), and nearly identical IC_50_ values between the latter. Analysis of the benzamide substitution at 2-position shows that unsubstituted ring **T-158** is more active than dimethyl and chloro-substituted derivatives **T-159** and **T-161**. Introduction of a phenyl ring at 3-position going from **T-158** to **T-169** results in a 1.5-fold decrease in activity, suggesting a detrimental effect of multiple bulky groups at 2-, and 3-position. Isomers comparison only reflects minor giardicidal activity differences for **T-157** vs. **T-168**, and **T-159** vs. **T-170**, and the bulkier group position effect is mixed, in the former case the bulkier at 3-position slightly favours while the latter is the bulkier at 2-position, suggesting the importance of their presence not their position. Like for n-butyl series the introduction of a trifluoromethyl group at 3-position leads to an increased giardicidal activity.

It was observed that for 7-position substitution, most derivatives are favoured by n-butyl chain substitution (6 out of 9 compounds) with differences in giardicidal activity going from 1.2 to 4.1-fold higher activity; still, there are derivatives (3 out of 9 compounds) that are favoured by iso-butyl chain substitution, but these only show 1.1- to 1.6-fold higher activity.

**Figure 1.**
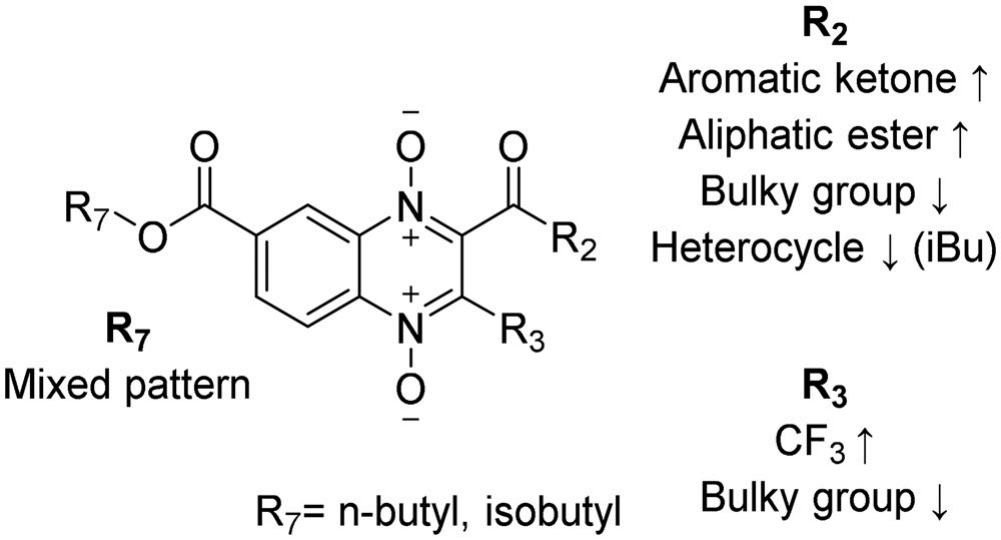
Structure–activity relationship summary for giardicidal activity.

In 2020, Barbosa *et al.* reported the giardicidal activity of methyl, ethyl, n-propyl, and iso-propyl quinoxaline-1,4-di-*N*-oxide derivative series with IC_50_ values 1.4–10930 nM^33^. There are 13 analogues between Barbosa’s study and the derivatives here reported, in eight of these compounds n-butyl and iso-butyl derivatives have a better giardicidal activity than all four previously reported series, for example, n-butyl, and iso-butyl derivatives **T-137** and **T-155** are 3- and 1.3-fold more active than its methyl analogue; n-butyl and iso-butyl derivatives with aliphatic esters at 2-position were ∼6–200-fold more active than their methyl, ethyl, n-propyl, and iso-propyl analogues; n-, and iso-butyl derivatives **T-142**, and **T-157** were ∼3–20-fold more active than their methyl, ethyl, and iso-propyl analogues. On the other hand, n-butyl derivative **T-146** is 4-fold less active than its n-propyl, and iso-propyl derivatives; n-butyl and iso-butyl derivatives **T-148**, and **T-163** are ∼6–10-fold less active than their methyl and iso-propyl analogues. Altogether, for the most part, the elongation of the chain at 7-position results in an increased giardicidal activity, still, there are few derivatives for which chain elongation results detrimental. A summary of the structure–activity relationship of the n-butyl and iso-butyl series is presented in Figure 1.

**Figure 2.**
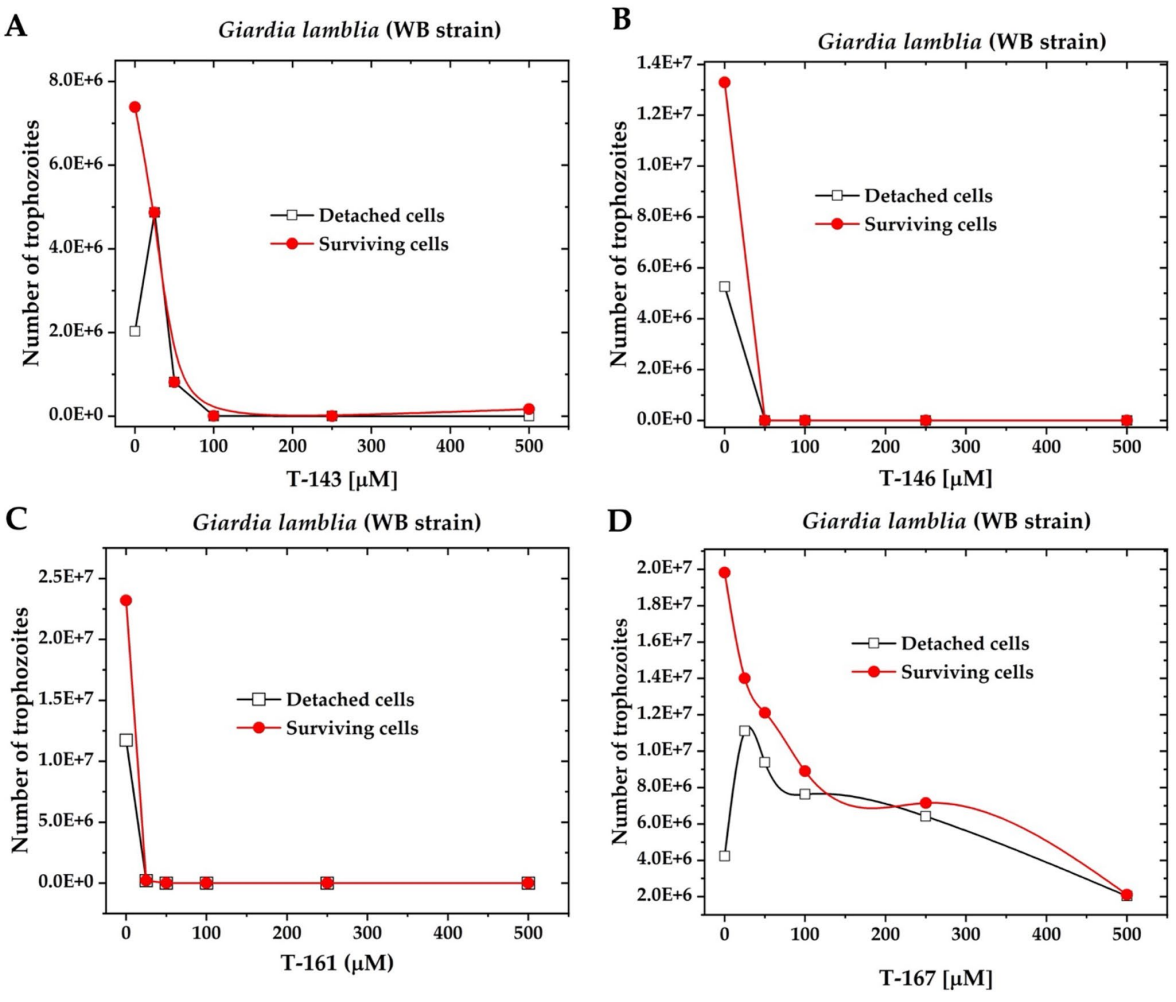
Effects of **T-143** (A), **T-146** (B), **T-161** (C), and **T-167** (D) on attachment and viability of *G. lamblia* trophozoites in increasing concentrations of the compounds.

Cytotoxicity of a drug is not the only important characteristic to cope with the pathogenesis of *G. lamblia*. It is known that the attachment of the trophozoites to the microvillus border of the small intestine is a crucial step for causing disease. Therefore, we assayed three compounds that were among the more effective in giardicidal effect (**T-143**, **T-146**, and **T-167**) and a compound with low giardicidal effect (**T-161**) against the WB strain to analyse the concentration effect on the number of detached and surviving cells. These four compounds were highly effective against *G. lamblia* trophozoites; detachment and cytotoxicity took impact with concentrations as low as 25 µM after 24 h of incubation at 37 °C (Figure 2). The observed effect for **T-143** and **T-167** showed a first step of cell detachment followed by a pronounced fall of trophozoites viability (Figure 2(A,C), respectively). **T-143** and **T-146** had the highest cytotoxic effect on giardia trophozoites (Figure 2(A,B), respectively). Detachment of trophozoites in cultures incubated with **T-146** or **T-161** was undetectable, with their impact directly through cytotoxicity (Figure 2(B)).

#### GlTIM molecular docking analysis

To know the potential mechanism of action of n-butyl and iso-butyl quinoxaline-1,4-di-*N*-oxide derivatives were evaluated by molecular docking based on two criteria: binding free energy (BFE) and protein-ligand interaction profile (PLIP) against triosephosphate isomerase of *G. lamblia.*

For triosephosphate isomerase from *G. lamblia* (*Gl*TIM), three potential sites were considered relevant to determine the inhibitory potential: residues part of the catalytic dyad (*Gl*TIM: K13, and H96) or nearby residues (*Gl*TIM: C14, Q65 and E98) and residues near to C222; additionally, the human triosephosphate isomerase (*Hs*TIM) was included to determinate a potential species-specific selectivity. The results are shown in Supplementary Table 1. BFE scores for the n-butyl and iso-butyl series at the active site range from −5.37 to −6.79 kcal/mol.

**Figure 3.**
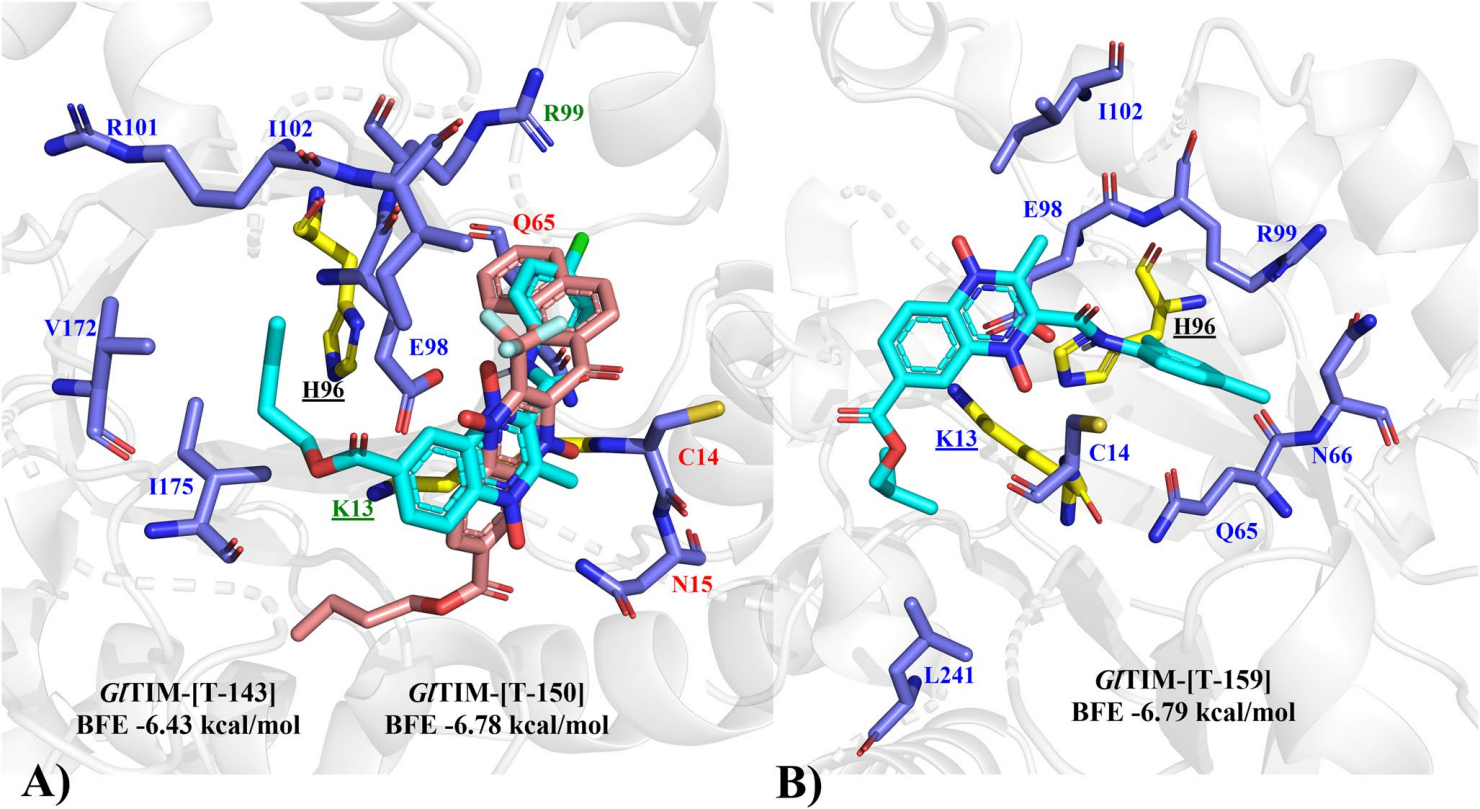
(A) Lead compounds structures for n-butyl series docked on *Gl*TIM: **T-143** (cyan), **T-150** (pink). Interactions: yellow/underlined: catalytic residues, blue label: **T-143** only, red label: **T-150** only, green label: shared, black label: not interacting. (B) Lead compound structure (**T-159**) for iso-butyl series docked on *Gl*TIM. Interactions: yellow/underlined: catalytic residues, blue label: interacting, black label: not interacting.

The presence of phenyl benzamides both substituted and unsubstituted at 2-position is the most common substituent in the quinoxaline derivatives scored with higher BFE values for *Gl*TIM active site binding: **T-143, T-144**, and **T-151** for n-butyl derivatives, and **T-158, T-159**, **T-161**, and **T-169** for iso-butyl derivatives. Similarly, the presence of aromatic ring substitutions at 2- or 3-position favours BFE value: **T-150, T-142**, **T-148**, and **T-149** for n-butyl series, and **T-157**, **T-165, T-166**, and **T-168** (3-position) for iso-butyl series. Altogether, pointing towards the importance of aromatic substituents at 2- and 3-position. The most common interaction profiles involve catalytic dyad K13, additionally with residues C14, E98, G176, and G238, which are in the vicinity of the catalytic dyad and hold a potential that if blocked, the enzymatic activity may be hindered. From the n-butyl series **T-143 (**Figure 3(A)) (−6.43 kcal/mol, π-cation K13, H-bond E98), and **T-150 **(Figure 3(A)) (−6.78 kcal/mol, π-cation, and salt bridge K13, H-bond C14, and N15), while for the iso-butyl series **T-159** (Figure 3(B)) (−6.79 kcal/mol, π-cation K13, H-bond C14, and E98) may be proposed with the most potential to behave as *Gl*TIM inhibitors, having highest BFE and interactions blocking access to catalytic cavity may be proposed with the most potential to behave as *Gl*TIM inhibitor.

**Figure 4.**
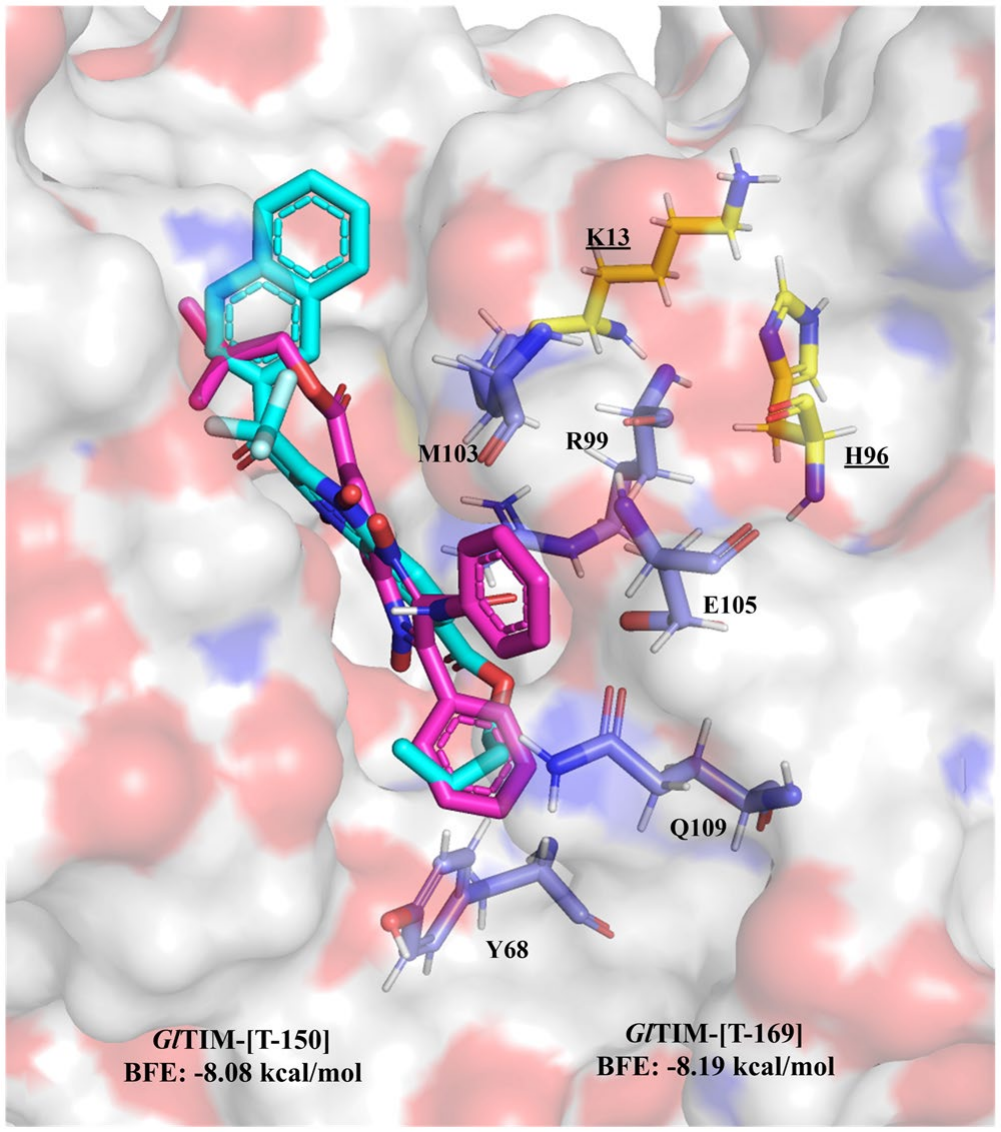
The top two n-butyl and iso-butyl quinoxaline derivatives bound at the *Gl*TIM interface, blocking the access to the cavity: **T-150** (cyan), and **T-169** (magenta). Yellow/underlined: catalytic residues, blue residues are the most common interactions among quinoxaline derivatives.

Exploration of alternative docking sites for *Gl*TIM revealed that the interface may be proposed as a better binding site for quinoxaline derivatives than the active site, as the BFE scores are better for this site than the active. The tendencies observed for the active site hold true for this site as well, as benzamides and other aromatic substituents at 2-position favoured BFE score. Most of the interactions that these compounds hold is with residues Y68, R99, M103, E105, and Q109, which reside at the cavity entrance of the interface and in the near vicinity of the catalytic site, thus suggesting that these may act as gatekeepers, not by directly interacting with the catalytic residues but by blocking the access to the cavity. Moreover, such interactions established between these compounds and the mentioned aminoacyl residues can possibly perturb the association between the two G*l*TIM monomers, as reported for *T. cruzi* TIM^43^. **T-150**, and **T-169** (Figure 4) show the best-predicted interaction on this site. The docking analysis near C222 (omeprazole derivatising residue)^44^^,^^45^ did not show any significant potential for any of the quinoxaline derivatives, as they are unlikely to form covalent bonds with C222; therefore, this inhibition may hardly occur in this study.

**Figure 5.**
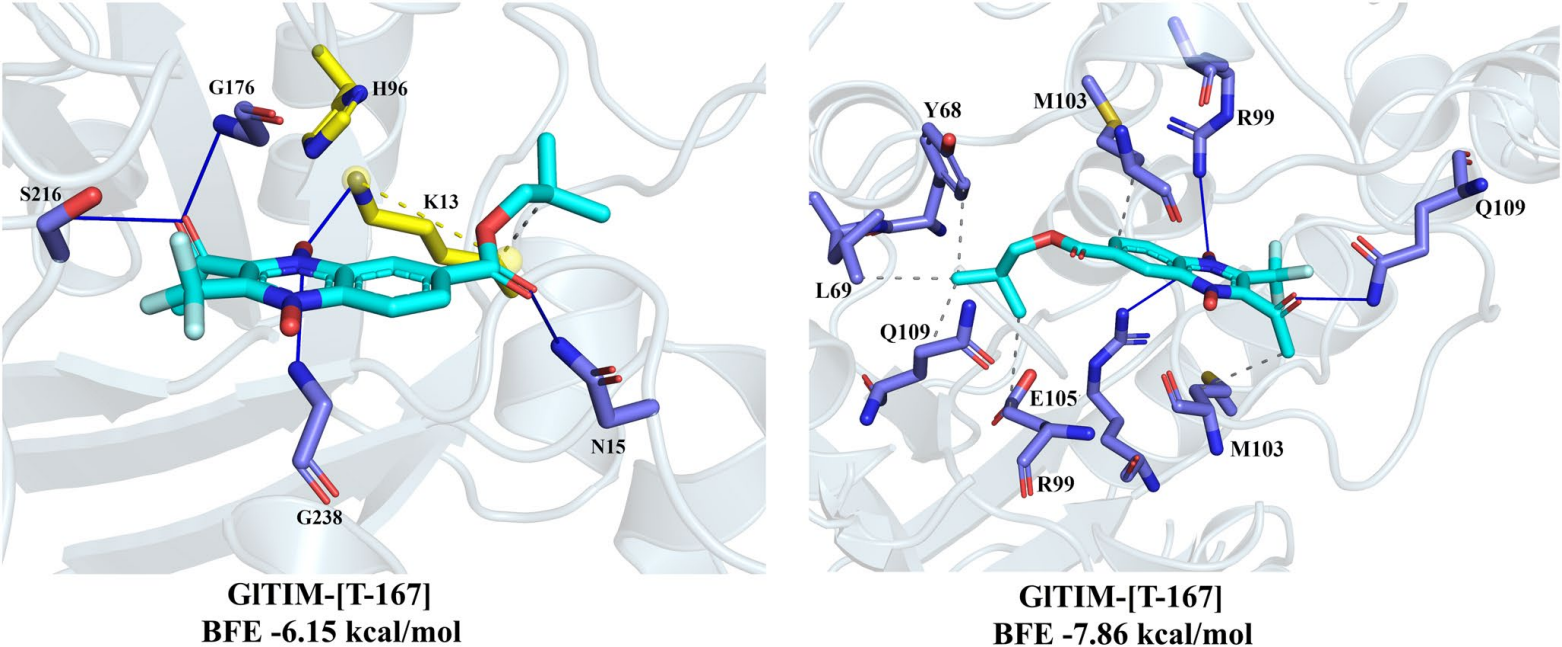
The predicted docking pose for **T-167,** the newly found GlTIM inhibitor, on the active site (A), and on the interface (B). Yellow residues at (A) panel represent the catalytic dyad.

Additional to the results observed for the top BFE scored compounds, it is noteworthy that the predicted interaction between **T-167** and the active site of *Hs*TIM did not show involvement of the important catalytic residue K13, thus being consistent with the behaviour observed in the *in vitro* assays, the docking pose is presented in Figure 5(A). While the docking for **T-167** at the interface shows interactions between the ligand and both interface chains at the cavity entrance suggesting that this could be a stable binding pose that permits inhibition Figure 5(B).

#### GlTIM enzymatic evaluation

**Figure 6.**
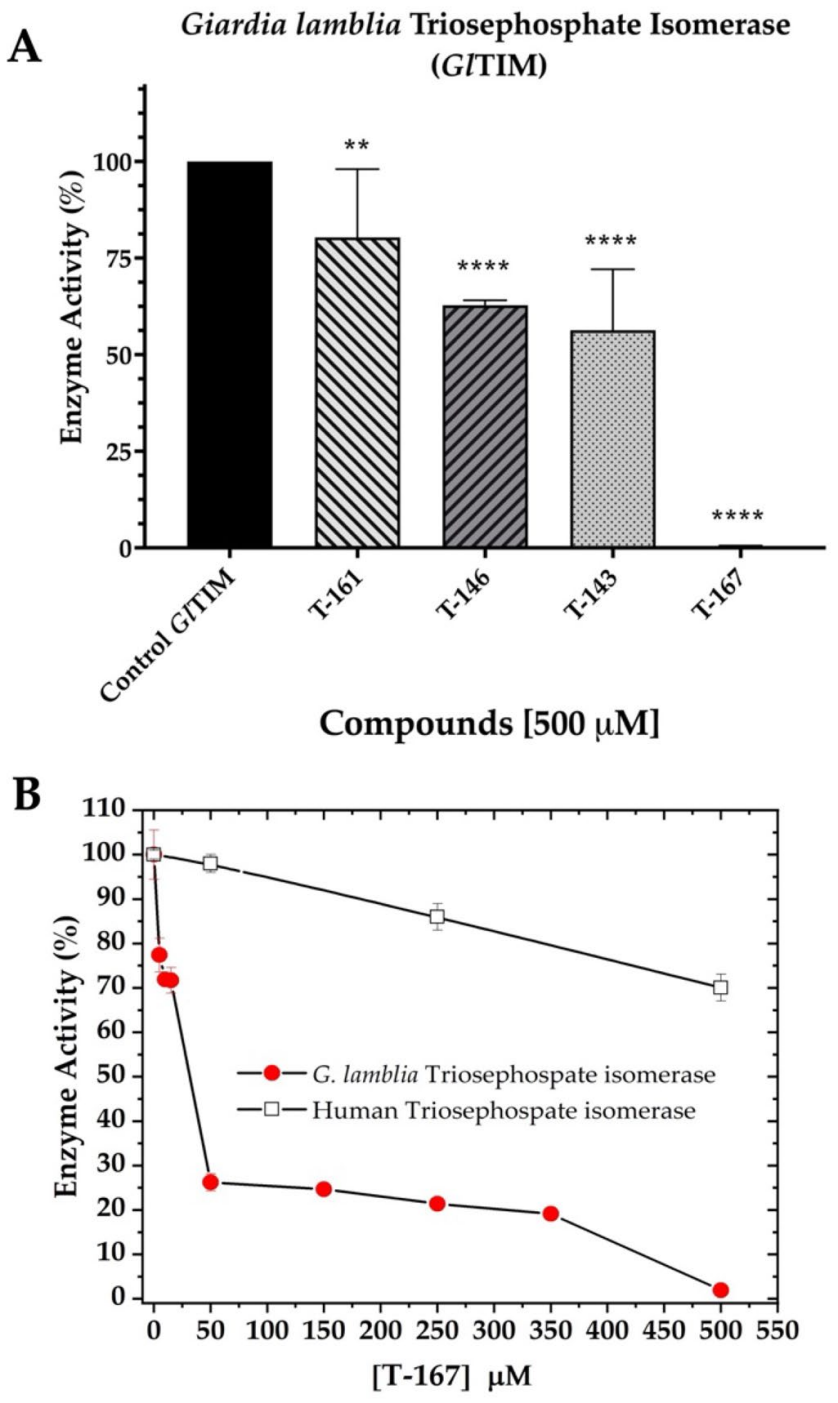
*In vitro* enzyme inhibition effects of **T-143**, **T-146**, **T-161**, and **T-167** on *Gl*TIM. (A) High concentration of these compounds shows the highest inhibitory efficiency to *Gl*TIM of **T-167** when compared with **T-143**, **T-146**, and **T-161.** (B) Kinetics of the inhibitory effects of **T-167** demonstrates its species-specific action.

We were able to perform enzyme inhibition assays on the recombinant *Gl*TIM for the most promising *in silico* (**T-143**), and the most giardicidal derivatives (**T-146**, and **T-167**), and low giardicidal agent and moderate *in silico* potential (**T-161**). One of the highest antigiardial concentrations (500 µM) of **T-143**, **T-146**, **T-161**, and **T-167** was tested through enzyme inhibition assays on the recombinant *Gl*TIM (Figure 6(A)). As shown, **T-167** was the most effective of these four compounds in inhibiting *Gl*TIM (99.7%), whereas **T-143**, **T-146**, and **T-161** only inhibited *Gl*TIM in 43.7%, 37.3%, and 19.7%, respectively. Based on these enzyme inhibition results, we analysed the effects of **T-167** in ranging concentrations against *Gl*TIM and *Hs*TIM (Figure 6(B)). As low as 10 µM concentration of **T-167** inhibited *Gl*TIM enzyme activity above 20%, whereas *Hs*TIM was unaffected.

Kinetics of the enzyme inhibition seems to indicate a three-step mechanism in which low concentrations of **T-167** (<10 to 50 µM) decrease enzyme activity of *Gl*TIM above 70% with almost no *Hs*TIM affectation as opposed to known inhibitor omeprazole which attains 70% inhibition at about 300 µM^44^. In the second phase of *Gl*TIM inhibition, it maintains a residual activity of ∼25% in a range of 50 to 350 µM concentrations of **T**-**167**. During this “stationary phase” of the inhibition process of *Gl*TIM, the *Hs*TIM is minimally affected by losing no more than 10% of its activity. After this, *Gl*TIM activity is drastically and wholly inhibited by 500 µM, as opposed to omeprazole causing similar effect by 750 µM^44^. Significantly, when *Gl*TIM activity is completely depleted, the *Hs*TIM retains more than 75% of its activity.

Integrating *in silico* predictions and *in vitro* observations, we may argue that although, **T-167**, was “lower” ranked in the predicted *Gl*TIM inhibition through interactions with its active site (-6.14 kcal/mol), it shows a high efficiency as a new molecule with exceptional giardicidal effects (IC_50_ 25.53 nM), and a potent *Gl*TIM inhibitory activity. The predicted interactions can be further discussed, in addition to interacting with the catalytic residue K13, T-167 interacts with the key amino acid residues S216 and G176 of *Gl*TIM, which play important roles in the conformational switch from open to closed of the flexible loop 6. S216 belongs to the highly conserved YGGS motif (residues 213–216 in loop 7) that interacts with loop 6 (residues 171–181) when the catalytic lid is in the closed conformation^46^^,^^47^. In *Gl*TIM, there is an H-bond between N^δ2^ of N221 and the peptide oxygen atom of S216, which also is H-bonded with S174 and G178. Molecular dynamics assays previously proposed that the affectation at this region directly impacts the enzyme activity of *Gl*TIM by impairing the hinge of the catalytic lid^48^.

Besides, **T-167** interacts with N15 residue, which has been pointed out in other TIMs as an inductor of critical structural changes in the interface (leading to loss of enzyme activity) when a negative charge is introduced at that position^40^. Moreover, **T-167** interacts with E105, which is contained in a conserved cluster of residues formed by N65, D77, R98, E104 (equivalent to E105 in *Gl*TIM), and K112 between the two subunits of TIMs. In this cluster, the two subunits are oriented face to face with ∼10Å distance between residues 105 of the two subunits. E105 is essential because its affectation can disrupt contacts of the amino acid side chains in the conserved cluster. This leads to a perturbation of the water network in which the water–protein and water–water interactions that join the two subunits are significantly weakened and diminished^49^.

#### Trichomonacidal activity

For n-butyl series, the effect of the aromatic ring size when comparing **T-148**, **T-149**, and **T-150** suggests that steric effect at 2-position is an important factor, as two-member ring **T-150** is the least active compound. The bio-isostere effect observed for trichomonacidal activity favours phenyl over thienyl at 2-position, suggesting sulphur atom addition results detrimental to trichomonacidal activity. The introduction of an ester at 2-position favours activity, as the change from ketone **T-137** to ester **T-138** leads to a nearly 3-fold increase in activity, the size of ester has only a minor effect, as small-size ester **T-138** has an IC_50_ of 88 nM, and mid-size **T-139**, **T-140**, and **T-146** have IC_50_ values around 100 nM, the change from an aliphatic ester to aromatic ester results in almost a 2-fold decrease in trichomonacidal activity. The introduction of substituted benzamides at 2-position favours trichomonacidal activity, where chloro-substituted on the phenyl ring **T-142** is the most active trichomonacidal agent from both series is 2.6-fold more active than dimethyl substituted **T-144**. The introduction of a trifluoromethyl at 3-position leads to a 2.4-fold increased trichomonacidal activity for **T-137** vs. **T-145** and a nearly negligible effect for **T-142** vs. **T-148**, and **T-139** vs. **T-146**.

For iso-butyl series, unlike n-butyl series, bulkier aromatic rings led to an increased trichomonacidal activity when comparing **T-163**, and **T-166** (phenyl vs naphthyl). Like n-butyl, the introduction of heterocycles at 2-position resulted in a slightly decreased trichomonacidal activity ∼1.2-fold decrease going from phenyl to thienyl or furyl (**T-163** vs **T-164** and **T-165**). Change from oxygen to sulphur atom for **T-164** and **T-165** has a negligible effect as these derivatives have nearly identical IC_50_ values. Introduction of substituted benzamide favours trichomonacidal activity, where dimethyl substituted on the phenyl ring is 1.3-fold more active than chloro- substituted, unsubstituted benzamide is least active. Introduction of a phenyl ring at 3-position going from **T-158** to **T-169** results in a 1.4-fold increase in activity, suggesting a favourable effect of multiple bulky groups at 2-, and 3-position. Isomers comparison reflects minor trichomonacidal activity differences for **T-157** vs. **T-168**, and **T-159 **vs.** T-170**, and the bulkier group position effect is mixed, in the former case the bulkier at 3-position slightly favours while the latter is the bulkier at 2-position, suggesting the importance of their presence not their position. The introduction of trifluoromethyl at 3-position leads to an increased trichomonacidal activity.

It was observed that most derivatives (5 out of 9 compounds) are favoured by n-butyl chain substitution at 7-position with differences in trichomonacidal activity going from 1.2 to 3-fold higher activity; still, there are derivatives (4 out of 9 compounds) that are favoured by iso-butyl chain substitution, but these only show 1.1- to 1.5-fold higher activity. A similar biological behaviour was observed for *G. lamblia.*

**Figure 7.**
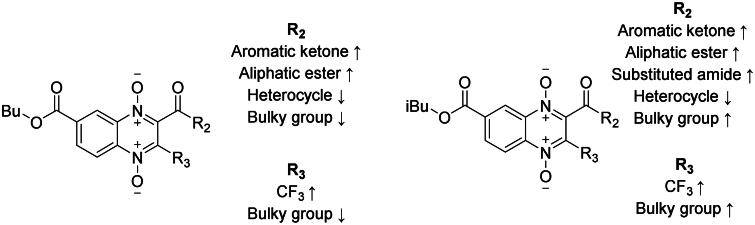
Structure–activity relationship summary for trichomonacidal activity.

The biological activity reported for each of the n-butyl and iso-butyl ester quinoxaline derivatives have better antiprotozoal effect (IC_50_ values ranging from 47–257 nM) than the methyl, ethyl, n-propyl, and iso-propyl ester quinoxaline derivatives previously reported by Palos *et al.* (2021) (IC_50_ values ∼1000–10000 nM). Our quinoxaline derivatives were one to two orders of magnitude more effective, indicating that the elongation of the ester chain drastically favoured trichomonacidal activity. Palos *et al.* (2021) also reported clear improved trichomonacidal activity for compounds bearing trifluoromethyl at 3-position, however, for n-butyl and iso-butyl derivatives in most cases introduction of trifluoromethyl results in a slight decrease in activity. Regarding 2-position, in the current study, most compounds were benefitted from aromatic substitutions regarding their trichomonacidal activity, meanwhile, in Palos *et al.*’s study esters and alkyl chains substitutions also favour the antiparasitic activity^35^. A summary of the structure-activity relationship of the n-butyl and iso-butyl series is presented in Figure 7.

#### TvTIM molecular docking analysis

To predict potential mechanism of action of n-butyl and iso-butyl quinoxaline-1,4-di-*N*-oxide derivatives were evaluated by molecular docking based against triosephosphate isomerase of *T. vaginalis.*

For *Tv*TIM one potential active site was considered to determine the inhibitory potential: residues part of the catalytic dyad (*Tv*TIM: K11, and H94) and nearby residues (*Tv*TIM: A12, E63, and E96); additionally, the *Hs*TIM was included to determinate a potential selectivity. The results are shown in Supplementary Table 2. BFE scores for the n-butyl and iso-butyl series range from −6.07 to −8.68 kcal/mol, all compounds except **T-137, T-138, T-139, T-155**, and **T-156** have a binding energy better than reported TIM inhibitor EQX-20^35^.

The presence of aromatic ring substitutions at 2-position **T-142**, **T-148**, and **T-150** for n-butyl series, and **T-157**, **T-163**, **T-166**, and **T-168** (3-position) for iso-butyl series. Similarly, the presence of phenyl benzamides both substituted and unsubstituted **T-143, T-144,** and **T-151** for n-butyl, and **T-158**, **T-161**, and **T-169** for iso-butyl. Altogether, pointing towards the importance of aromatic substituents at 2- and 3-position.

**Figure 8.**
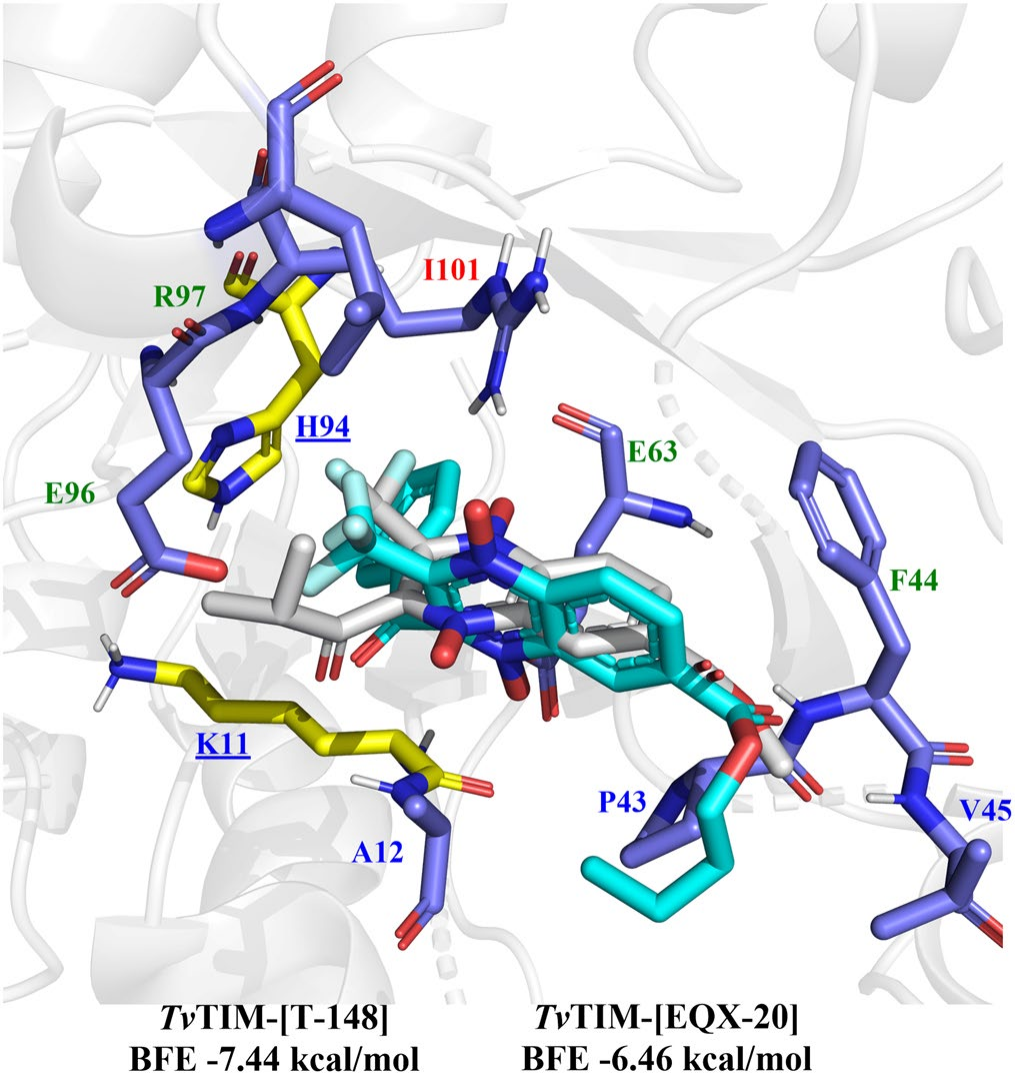
Lead compound structure for n-butyl series docked on *Tv*TIM and control EQX-20: **T-148** (cyan), EQX-20 (white). Interactions: yellow/underlined: catalytic residues, blue label: **T-148** only, red label: EQX-20 only, green label: shared.

Twelve from the fourteen n-butyl derivatives bear an interaction with at least one residue from the catalytic dyad, while **T-148** (Figure 8) bears interaction with both residues from the dyad, thus suggesting that this is the most promising derivative among the n-butyl series to act as a *Tv*TIM inhibitor. Additionally, **T-148** has a high trichomonacidal activity suggesting that its mode of action may be related to *Tv*TIM inhibition.

**Figure 9.**
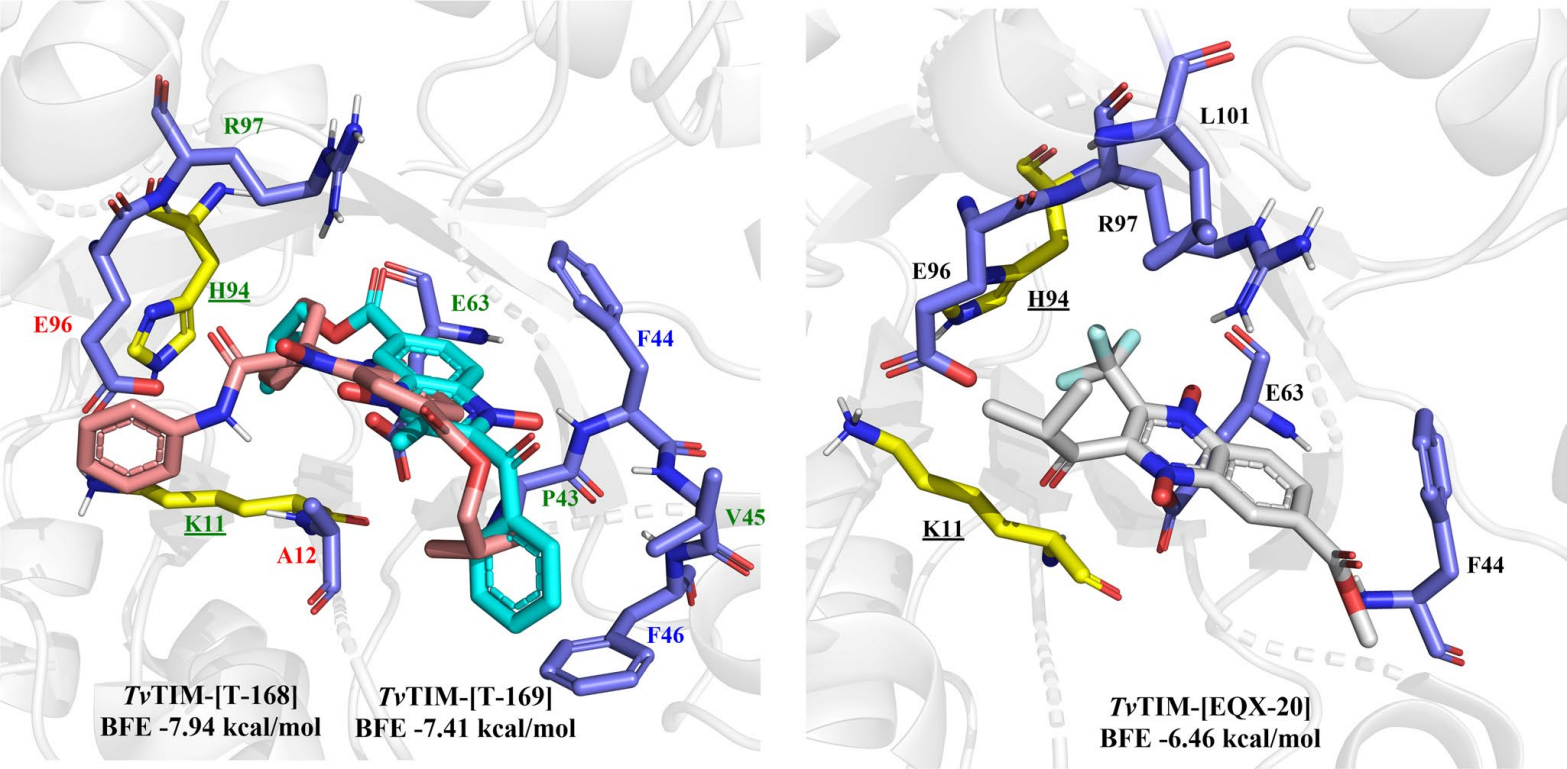
Lead compounds structures for iso-butyl series docked on *Tv*TIM and control EQX-20: **T-168** (cyan), **T-169** (pink), EQX-20 (white). Interactions: yellow/underlined: catalytic residues, blue label: **T-168** only, red label: **T-169** only, green label: shared, black label: not interacting (EQX-20 all interact except catalytic residues).

In the case of the iso-butyl series, eleven of the fourteen derivatives bear interaction with at least one residue from the catalytic dyad, and seven of these eleven derivatives hold hydrophobic interactions with both residues from dyad K11 and H94: **T-158, T-161**, **T-164, T-165, T-168**, and **T-169,** these last two being the most promising *Tv*TIM inhibitors. All other interactions near the catalytic dyad occur with Ala12, Glu63, and Glu96, all of which are residues oriented towards the dyad (Figure 9). Notably, all compounds had a better BFE score towards parasitic than human protein, thus suggesting their good trichomonicidal activity may be related to their potential to behave as moderate to high selective inhibitors of *Tv*TIM.

#### Amoebicidal activity

For n-butyl series, the effect of the aromatic ring size when comparing **T-148**, **T-149**, and **T-150** suggests that steric effect at 2-position is an important factor, as two-member ring **T-150** is the least active compound. Phenyl and thienyl at 2-position function as true bio-isosteres, as they have nearly identical amoebicidal activity. The introduction of an ester favours activity, ester length plays only a minor role, as mid-size esters **T-139**, and **T-140** have IC_50_ values around 30 nM, while small ester **T-138** and bulkier **T-141** have IC_50_ values around 40 nM. On the other hand, the nature of the ester plays a negligible role, as the change from an aliphatic ester to an aromatic ester (**T-141**) causes nearly no change in amoebicidal activity. The introduction of substituted benzamides at 2-position favours activity, where chloro-substituted on phenyl ring is ∼3-fold more active than dimethyl-substituted. The introduction of a trifluoromethyl at 3-position leads to a 1.75- and 1.88-fold decrease in amoebicidal activity for **T-142** vs. **T-148** and **T-139** vs. **T-146** respectively, while a 1.4-fold increase for **T-137** vs. **T-145**.

For iso-butyl series, unlike n-butyl series, bulkier aromatic rings led to an increased amoebicidal activity when comparing **T-163**, and **T-166** (phenyl vs. naphthyl). The introduction of heterocycles at 2-position resulted in an increased amoebicidal activity ∼2-fold increase going from phenyl to thienyl or furyl (**T-163** vs. **T-164** and **T-165**). Change from oxygen to sulphur atom for **T-164** and **T-165** has a negligible effect as these derivatives have nearly identical IC_50_ values. Introduction of benzamide at 2-position favours amoebicidal activity, where dimethyl and chloro- substituted on the phenyl ring have nearly identical IC_50_ values around 30 nM, while unsubstituted benzamides are least active around 40 nM. Introduction of a phenyl ring at 3-position going from **T-158** to **T-169** results in a 1.16-fold decrease in activity, suggesting a minor detrimental effect of multiple bulky groups at 2-, and 3-position. Isomers comparison reflects minor amoebicidal activity differences for **T-157** vs. **T-168**, and **T-159** vs. **T-170**, and the bulkier group position effect is mixed, in the former case the bulkier at 3-position slightly favours while the latter is the bulkier at 2-position, suggesting the importance of their presence not their position. The introduction of a trifluoromethyl group at 3-position leads to a slight decrease in amoebicidal activity. Still, there are derivatives which bear a trifluoromethyl group at 3-position which have an important amoebicidal activity.

Most derivatives (7 out of 9 compounds) are favoured by n-butyl chain substitution at 7-position with differences in amoebicidal activity going from 1.1 to 5.5-fold higher activity; still, there are derivatives (2 out of 9 compounds) that are favoured by iso-butyl chain substitution, but these only show 1.5-fold higher activity. A similar biological behaviour was observed against *G. lamblia,* and *T. vaginalis.*

**Figure 10.**
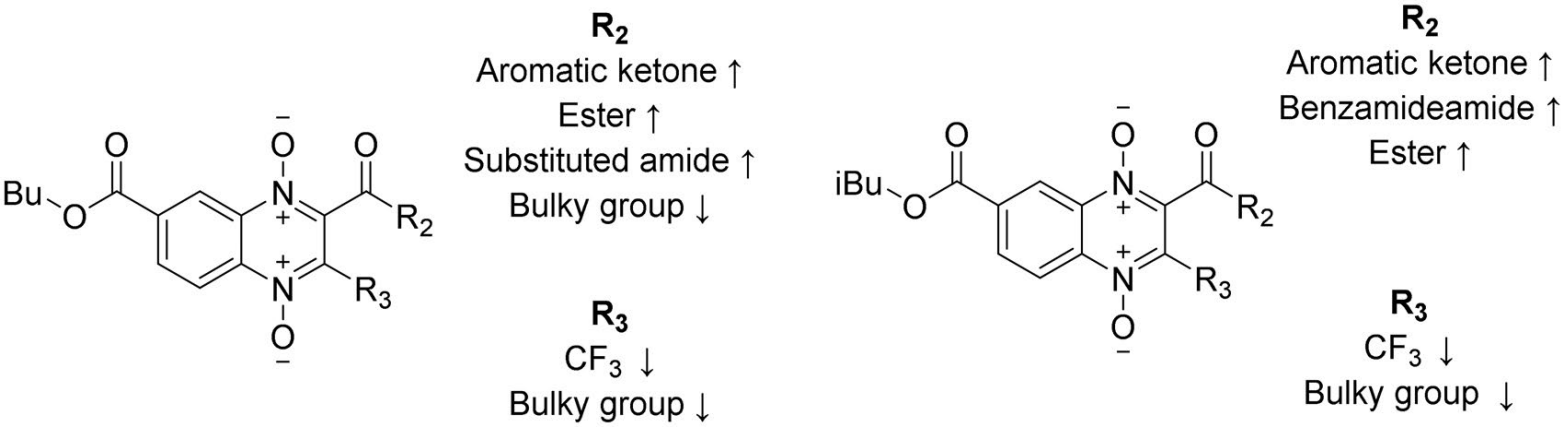
Structure–activity relationship summary for amoebicidal activity.

Two previous studies using esters of methyl and ethyl^20^, and n-propyl and iso-propyl^34^ quinoxaline-1,4-di-*N-*oxide derivatives have been reported. All compounds reported in these two previous studies show a lower amoebicidal activity (IC_50_ ∼300–2000 nM) than n-butyl and iso-butyl derivatives reported in the present study (IC_50_ 9–83 nM), thus suggesting that the chain elongation at 7-position enhance the biological activity. The findings in Duque-Montaño and Soto-Sanchez’s studies contrast with this study, as in this study trifluoromethyl group at 3-position slightly lowers activity. Earlier investigations revealed that aromatic rings, aliphatic chains, and esters substitutions 2-position favoured amoebicidal activity, which coincides with our findings that aromatic rings and esters favour amoebicidal activity. A summary of the structure–activity relationship of the n-butyl and iso-butyl series is presented in Figure 10.

#### EhTrxR molecular docking analysis

To know the potential mechanism of action of n-butyl and iso-butyl quinoxaline-1,4-di-*N*-oxide derivatives were evaluated by molecular docking against thioredoxin reductase of *E. histolytica.*

In the case of thioredoxin reductase from *E. histolytica* (*Eh*TrxR) two potential active sites were considered as relevant to determine the inhibitory potential: the residues that naturally interact with the NADPH binding site (K122, G160, A163, H182, R183, and R291) and redox active site (C140, C143), which have a slight overlap, such finding agreed to the sites predicted by Soto-Sánchez *et al.* (2020) using MOE software^34^. The results obtained are shown in Supplementary Table 3. BFE scores for the n-butyl and iso-butyl series range from −6.46 to −8.63 kcal/mol at the NADPH site, all having a higher affinity than metronidazole (−4.76 kcal/mol) and −6.15 to −8.4 kcal/mol for active site. The interaction profile observed for the different derivatives shows that interactions with NADPH binding residues are the most commonly present, while interactions with catalytic residue C140 rarely appears, and with C143 does not occur.

The presence of phenyl benzamides both substituted and unsubstituted at 2-position is the most common substituent in the quinoxaline derivatives scored with higher BFE values for *Eh*TrxR active site binding: **T-143, T-144**, and **T-151** for n-butyl derivatives, and **T-158, T-159**, **T-161** at 2-position, and **T-170** at 3-position for iso-butyl derivatives. Similarly, the presence of aromatic ring substitutions at 2- or 3-position favours BFE value: **T-150, T-142**, **T-148**, and **T-149** for n-butyl series, and **T-157**, **T-163**, **T-165, T-166**, and **T-168** (3-position) for iso-butyl series. Altogether, pointing towards the importance of aromatic substituents at 2- and 3-position. These same characteristics are preserved for the active site docking, where these same compounds are scored top suggesting that these substituents at 2- and 3-position favour *Eh*TrxR inhibition at two different docking sites.

**Figure 11.**
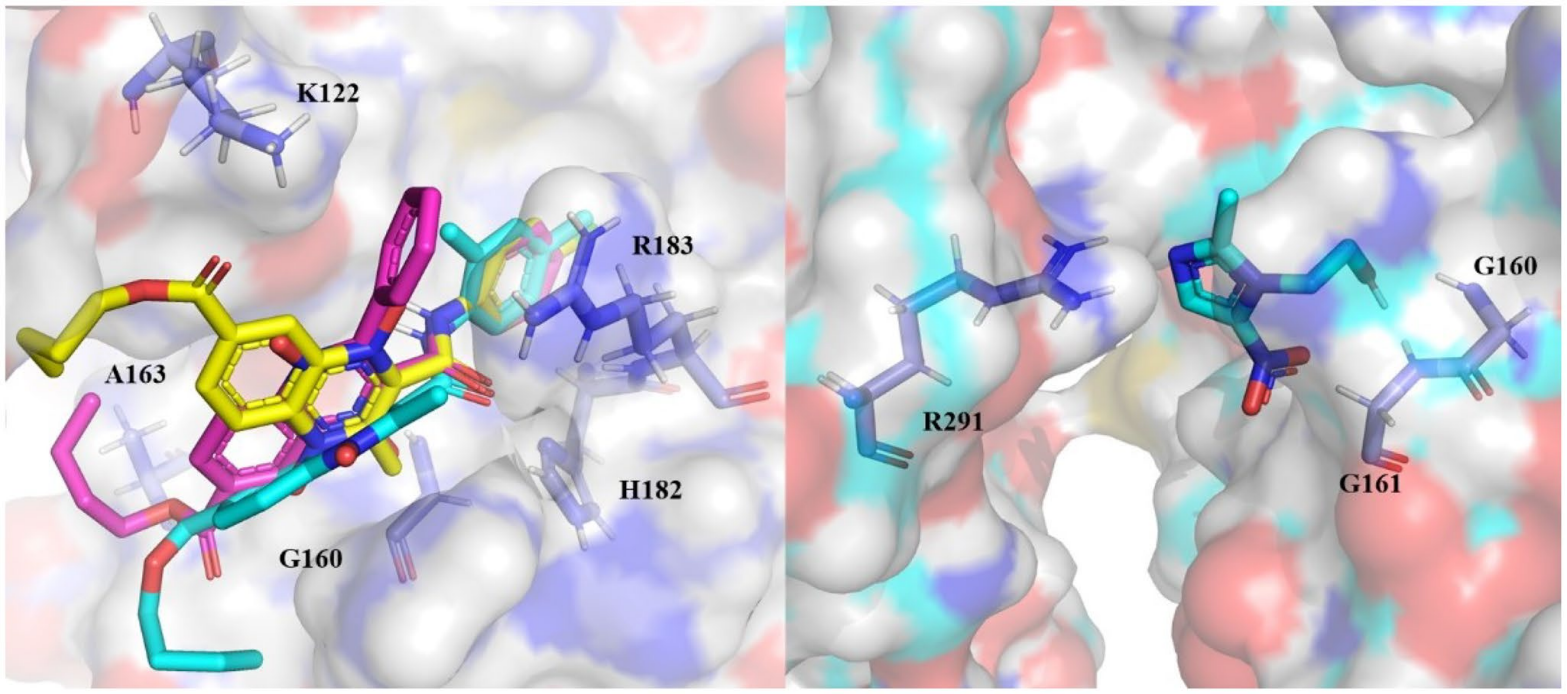
Compounds from the n-butyl series bearing benzamide substituents docked on the NADPH site of *Eh*TrxR and docked pose for metronidazole (right), (residues displayed bind NADPH to *Eh*TrxR).

For the n-butyl series, of the 14 derivatives, 11 bear at least two polar (H-bond, salt bridge, or π-cation) interactions with the residues that bind NADPH to *Eh*TrxR (K122, G160, A163, H182, R183, and R291) all five from the top-scored fulfilled this criterion: **T-141, T-143**, **T-144**, **T-150**, and **T-151**. The benzamide-substituted **T-143**, **T-144**, and **T-151** orientation on the binding pocket suggests that the amide geometry favours this binding (Figure 11), thus suggesting their potential as *Eh*TrxR inhibitors. The docking centre on the active site resulted in all compounds bound to a nearby cavity but without interactions with the catalytic triad, thus suggesting that the quinoxaline derivatives do not behave as inhibitors at the disulphide active site (C140, and C143).

**Figure 12.**
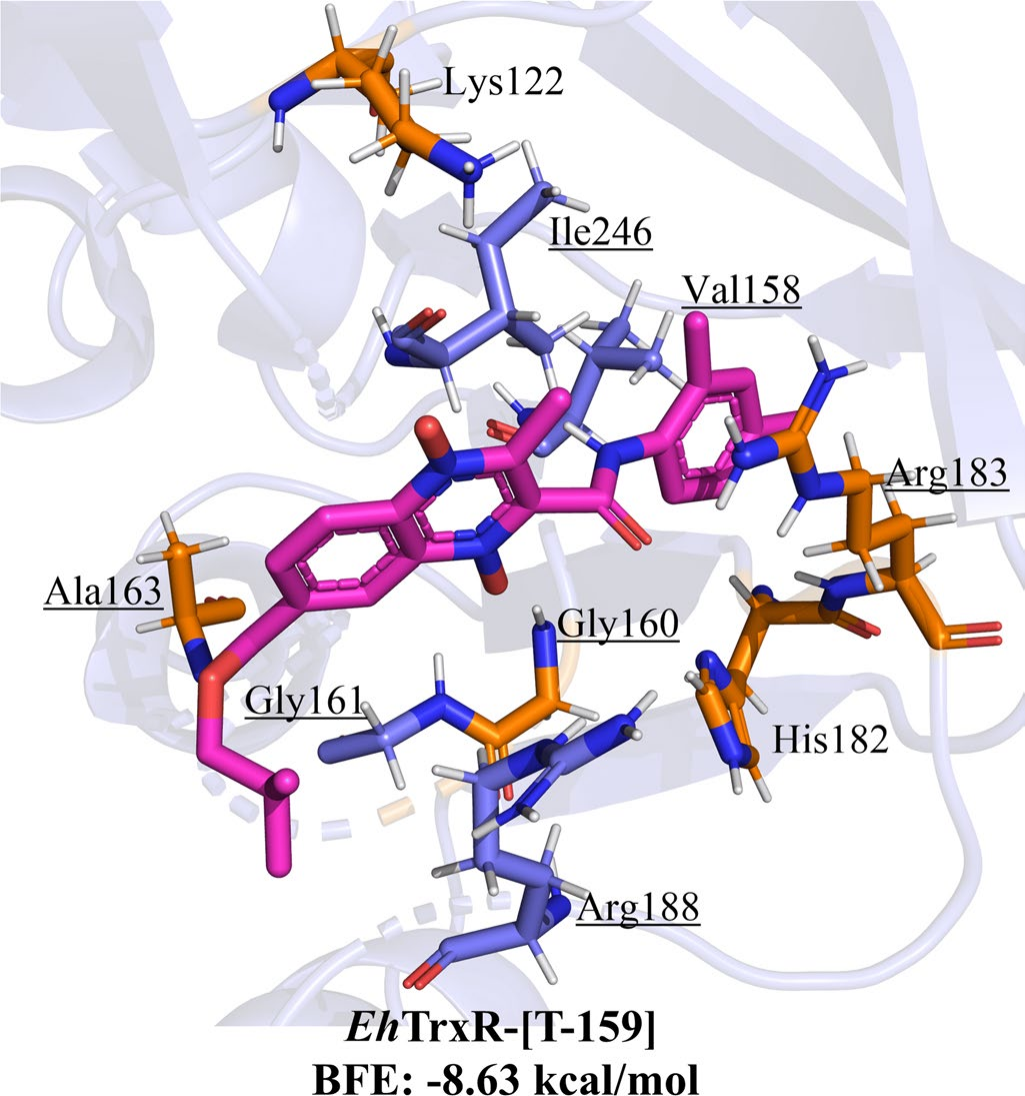
The most promising compound **T-159** (magenta) docked on the NADPH site of *Eh*TrxR, and control metronidazole (white), (orange: NADPH binding ­residues, underlined: interacting residues).

For the iso-butyl series, of the fourteen derivatives, ten bear at least two polar (H-bond, salt bridge, or π-cation) interactions with the residues that bind NADPH to *Eh*TrxR. Interestingly, **T-158, T159, **and** T161** are three of the top-scored that bear interactions with three residues that bind NADPH. After considering both criterion BFE and PLIP, it is suggested that **T-159** has the highest potential to behave as *Eh*TrxR, with a BFE of −8.63 kcal/mol and three interactions with residues that bind NADPH (Figure 12), and as a second potential derivative from this series is **T-161** with BFE −7.84 kcal/mol and three polar interactions with NADPH-binding residues. Derivative **T-161** was shown *in vitro* that it holds an inhibitory activity against *Eh*TrxR.

#### EhTrxR enzymatic evaluation

**Figure 13.**
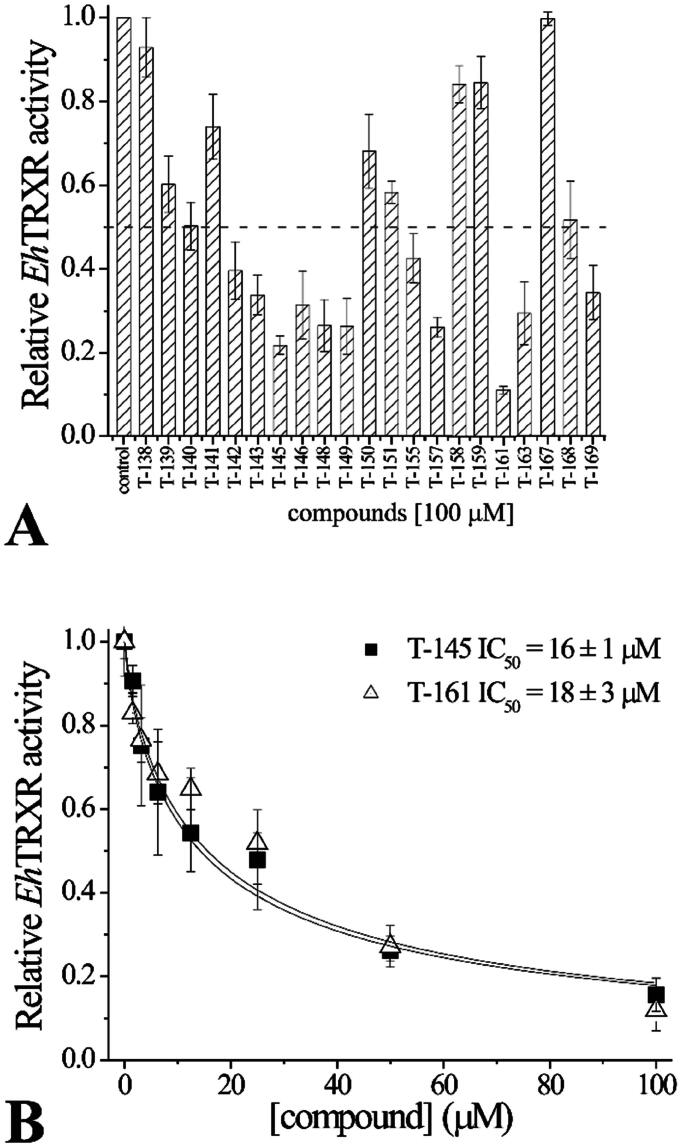
Evaluation of the inhibitory effect of quinoxaline-1,4-di-*N*-oxide derivatives on *Eh*TrxR activity. (A) Inhibition of disulphide reductase activity; assays were performed at 30 °C and pH 7.0 in the presence of 5 mM DTNB, 300 µM NADPH, 0.1 µM *Eh*TRXR, in the absence or in the presence of 100 μM of the respective compound. The activities were normalised to measurements performed in the absence of the compounds. (B) *Eh*TRXR disulphide reductase activity inhibition profiles at different concentrations of **T-145**, or **T-161**. The activities were normalised to measurements performed in the absence of the compounds. All results are displayed as mean ± standard error of three independent experiments.

We evaluated the putative inhibitory effect of quinoxaline-1,4-di-*N*-oxide derivatives on disulphide reductase activity of recombinant *Eh*TrxR using DTNB as direct disulphide substrate. The compounds were evaluated through enzyme inhibition assays on the recombinant *Eh*TrxR (Figure 13(A)) at concentration of 100 µM. As shown, the compounds **T-140**, **T-142**, **T-143**, **T-146**, **T-148**, **T-149**, **T-155**, **T-157**, **T-163**, and **T-169** exhibited an inhibitory effect between 50 to 75%. In addition, the compounds **T-145**, and **T-161** presented an inhibitory effect higher than 75%. The assays showed that the compounds **T-145**, and **T-161** were the best inhibitors, with IC_50_ values of 16 ± 1 µM, and 18 ± 3 µM, respectively (Figure 13(B)).

**Figure 14.**
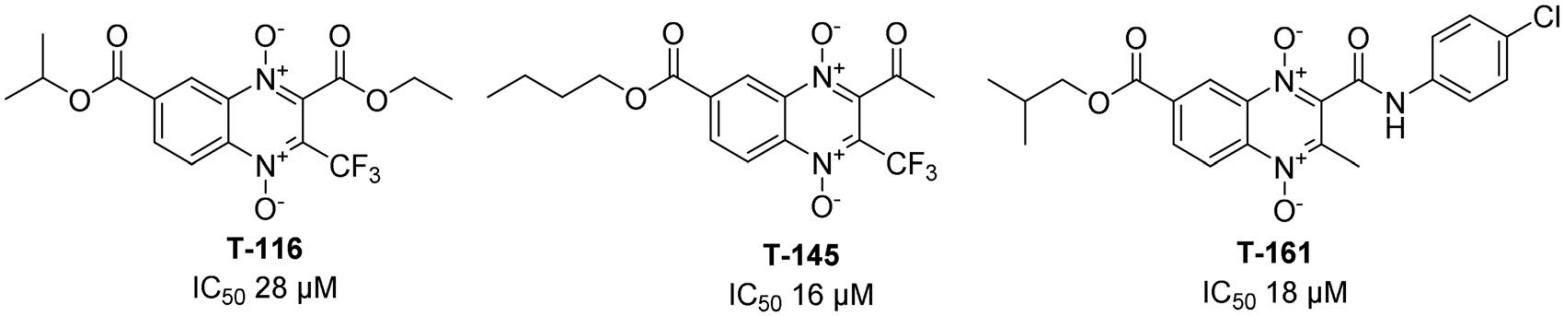
Structures for previously reported *Eh*TrxR inhibitor T-116, and newly found *Eh*TrxR inhibitors T-145, and T-161.

The activity observed for both inhibitors **T-145**, and **T-161**, shows IC_50_ values of 16, and 18 µM respectively, as opposed to **T-116** reported by Sóto-Sánchez *et al.* in 2020 which showed an inhibition of 28 µM^34^, thus proposing them as better inhibitors that these previous findings. Substitutions at 7-position show that the change from a 3-carbon alkyl of **T-116** chain to 4-carbon of **T-145**, and **T-161** favours *Eh*TrxR inhibition, similarly it may be observed that trifluoromethyl substitution is present at 3-position in both **T-116**, and **T-145** suggesting a potential benefit of including such substituent. While **T-116** bears an ethyl ester at 2-position, **T-145** bears an acetyl group, and **T-161** bears a 4-chlorobenzamide (Figure 14), not a clear tendency as to type of substitution is most favourable, instead suggesting that not a single functional group is responsible for the inhibition. Additionally, putting the *in silico* predicted behaviour and observed inhibition it is possible to propose that **T-145**, and **T-161** may act as inhibitors at the NADPH binding site, as they are predicted to interact with residues important for the binding of the natural dinucleotide ligand Ala163, and Arg183.

### Cytotoxic activity

The compounds were measured their cytotoxic activity against macrophages J774.2, where they ranged from 17650 to over 200000 nM (Table 1), thus showing selectivity index values ranging from 190 to 7660-fold more active against parasites over macrophages. The selectivity indices for the most active compounds **T-142** (1918-fold more active *E. histolytica* versus macrophages), **T-143** (1726-, 1112-, and 3321-fold more active for *G. lamblia*, *T. vaginalis*, and *E. histolytica* versus macrophages respectively), and **T-167** (994-fold more active against *G. lamblia* versus macrophages). Additional to the selectivity of the most active compounds it was observed that all tested quinoxalines showed selectivity over 10^2^-fold more activity against parasites over mammalian macrophages, thus presenting these series of compounds as potentially safe candidates for further studies.

## Conclusions

In this study, n-butyl, and iso-butyl quinoxaline-7-carboxylate 1,4-di-*N*-oxide derivatives were tested as new giardicidal, amoebicidal, and trichomonacidal agents. Five compounds (**T-143**, **T-146**, **T-149**, **T-155,** and **T-167**) had better giardicidal activity (IC_50_ < 30 nM), and five compounds (**T-138**, **T-142**, **T-143**, **T-148**, and **T-167**) had better trichomonacidal activity (IC_50_ < 90 nM) that reference drugs, albendazole, and metronidazole. Both biological activity effects are favoured by the presence of aliphatic esters at 2-position, and trifluoromethyl group at 3-position on the quinoxaline ring. Additionally, compounds with better amoebicidal activity than reference drugs were **T-142**, **T-143**, **T-148**, and **T-149**. Interestingly, compound **T-143** is highlighted as the best broad-spectrum activity. This biological effect is favoured by the presence of aromatic ketones and substituted amides at 2-position.

The predicted *Gl*TIM inhibition quinoxaline-7-carboxylate 1,4-di-*N*-oxide derivatives on the active site was favoured by the presence of aromatic rings at 2- or 3- position, either as ketones, benzamides, or esters, permitting important BFE values and interactions with catalytic residue: K13, and neighbouring residues E98, G176. Interestingly, *in vitro* enzyme inhibition assays demonstrated that **c**ompound **T-167** is a potent and specific *Gl*TIM inhibitor that wholly inactivates parasitic protein, while leaving over 75% of human TIM activity unhindered. Although, others pharmacological targets for **T-167** in *G. lamblia* cannot be discarded, our results strongly support that its giardicidal effects could be understood through the inhibition of *Gl*TIM.

The predicted *Tv*TIM inhibition was favoured, like *Gl*TIM, by the presence of aromatic rings at 2- or 3- position, either as ketones, benzamides, or esters, permitting better BFE values than reported inhibitor EQX20 (-6.46 kcal/mol), and interactions with catalytic residues: K11, and H94, and neighbouring residues E63, and E96. The highest potential for *Tv*TIM inhibition was observed for **T-148** (-7.44 kcal/mol), **T-168** (-7.94 kcal/mol), and **T-169** (-7.41 kcal/mol). Therefore, the moderate trichomonacidal activity of **T-148** may be proposed because of the inhibition of *Tv*TIM.

The predicted *Eh*TrxR inhibition potential was favoured by the presence of aromatic rings and benzamides at 2- or 3-position, permitting interactions with G160, A163, and R183, which are residues that participate in the NADPH natural binding, displaying **T-159** the best inhibitory potential, although, in *in vitro* studies had a low inhibition. Compounds **T-145**, and **T-161** were the best *Eh*TrxR inhibitors with IC_50_ of 16 ± 1 µM, and 18 ± 3 µM, respectively. Therefore, it’s necessary to continue exploring the potential binding site for these derivatives.

Finally, these results support the use of esters of quinoxaline 1,4-di-*N*-oxide derivatives to develop new specific inhibitors of *Gl*TIM and *Eh*TrxR as potent giardicidal and amoebicidal agents, respectively. Although, more studies are necessary to know the specific binding site on the pharmacological targets.

## Contributions statement

Conceptualisation, G.R. and A.G.G.; methodology, A.G.G., O.S.S., L.Y.M., G.L.V., J.I.M.M., T.D.M., L.K.V.J., S.P.G., L.C.R., D.G.A., A.M.R., A.D.P.G., and E.O.P.; computational studies data interpretation and analysis, A.G., T.D.M., L.K.V.J.; validation, G.R., and A.G.; formal analysis, A.G.G., and G.R.; investigation, A.G.G., O.S.S.; resources, G.R.; data curation, L.Y.M., G.L.V., D.G.A., and G.R.; writing—original draft preparation, A.G.G., and G.R.; writing—review and editing, A.G,G., O.S.S., T.D.M., L.K.V.J., L.Y.M., G.L.V., D.G.A., A.M.R., and G.R.; visualisation, A.G.G., and G.R.; supervision, G.R., L.Y.M., G.L.V., D.G.A., and A.M.R.; project administration, G.R.; funding acquisition, G.R. All authors have read and agreed to the published version of the manuscript.

## Supplementary Material

24092024_SuppMat_JEIMC_1_.docx

## Data Availability

The data that support this study can be made available from the corresponding author upon reasonable request.
