# Supplementary material for: Expanding the antiprotozoal activity and the mechanism of action of n-butyl and iso-butyl ester of quinoxaline-1,4-di-N-oxide derivatives against Giardia lamblia, Trichomonas vaginalis, and Entamoeba histolytica. An in vitro and in silico approach
Source: J Enzyme Inhib Med Chem. 2024 Oct 29;39(1):2413018. doi: 10.1080/14756366.2024.2413018 (PMC11523249; doi:10.1080/14756366.2024.2413018)
Supplement: 24092024_SuppMat_JEIMC_1_.docx [file IENZ_A_2413018_SM6283.docx]

SUPPLEMENTARY INFORMATION

Expanding the Antiprotozoal Activity and the Mechanism of Action of n-Butyl and iso-Butyl Ester of Quinoxaline-1,4-di-*N*-oxide Derivatives against *Giardia lamblia*, *Trichomonas vaginalis*, and *Entamoeba histolytica.* An *in vitro* and *in silico* Approach

Alonzo González-González^a^, Oscar Sánchez-Sánchez^a^, Lilián Yépez-Mulia^b^, Timoteo Delgado-Maldonado^a^, Lenci K. Vázquez-Jiménez^a^, Gabriel López-Velázquez^c^, José Ignacio de la Mora-de la Mora^c^, Sebastián Pacheco-Gutierrez^c^, Laura Chino-Ríos^c^, Diego G. Arias^d,e^, Adriana Moreno-Rodríguez^f^, Alma D. Paz-González^a^, Eyra Ortíz-Pérez^a^, Gildardo Rivera^a*^

^a^ Laboratorio de Biotecnología Farmacéutica, Centro de Biotecnología Genómica, Instituto Politécnico Nacional, Reynosa 88710, México, ^b^ Unidad de Investigación Médica en Enfermedades Infecciosas y Parasitarias-Pediatría, Hospital de Pediatría, Instituto Mexicano del Seguro Social, México City 06720, México, ^c^ Laboratorio de Biomoléculas y Salud Infantil, Instituto Nacional de Pediatría, México City 04530, México, ^d^ Laboratorio de Enzimología Molecular, Instituto de Agrobiotecnología del Litoral (CONICET-UNL), Santa Fe, Argentina, ^e^ Facultad de Bioquímica y Ciencias Biológicas, Universidad Nacional del Litoral, Santa Fe, Argentina, ^f^ Laboratorio de Estudios Epidemiológicos, Clínicos, Diseños Experimentales e Investigación, Facultad de Ciencias Químicas, Universidad Autónoma “Benito Juárez” de Oaxaca

*** Corresponding author:** Ph.D Gildardo Rivera, Laboratorio de Biotecnología Farmacéutica, Centro de Biotecnología Genómica, Instituto Politécnico Nacional, Boulevard del Maestro, S/N, Col. Narciso Mendoza, Reynosa 88710, México. Phone: +52 899 9243627. E-mail: giriveras@ipn.mx

**Structural elucidation**

**T-137**: 3-acetyl-6-(butoxycarbonyl)-2-methylquinoxaline 1,4-dioxide. 2.37 % yield, yellow solid (80-82 °C). FT IR (ν cm^−1^): 2900-3000 (ArC-H); 2900-2800 (C-H); 1727, 1700 (C=O); 1323 (*N-*oxide); 1160-1300 ((C=O)-C-O). ^1^H NMR (400 MHz, DMSO) δ 8.97 – 8.92 (m, 1H), 8.55 (dd, *J* = 9.0, 0.6 Hz, 1H), 8.37 (dd, *J* = 8.9, 1.8 Hz, 1H), 4.41 (t, *J* = 6.5 Hz, 2H), 2.67 (s, 3H), 2.40 (s, 3H), 1.84 – 1.72 (m, 2H), 1.55 – 1.41 (m, 2H), 0.98 (t, *J* = 7.4 Hz, 3H). ^13^C-NMR (101 MHz, DMSO) δ 195.61, 164.58, 141.15, 139.73, 138.88, 137.86, 133.48, 130.93, 121.58, 121.22, 66.06, 30.60, 29.96, 19.18, 14.30, 14.02. UPLC-MS: rt. 1.15 min, 319.05 m/z.

**T-138**: 6-(butoxycarbonyl)-3-(methoxycarbonyl)-2-methylquinoxaline 1,4-dioxide. 1.69 % yield, dark red think oil. FT IR (ν cm^−1^): 2900-3000 (ArC-H); 2900-2800 (C-H); 1745, 1719 (C=O); 1331 (*N-*oxide); 1160-1300 ((C=O)-C-O). ^1^H NMR (400 MHz, DMSO) δ 8.95 – 8.85 (m, 1H), 8.61 – 8.50 (m, 1H), 8.38 (ddd, *J* = 14.0, 8.9, 1.8 Hz, 1H), 4.41 (t, *J* = 6.6 Hz, 2H), 4.04 (s, 3H), 2.46 (s, 3H), 1.79 – 1.74 (m, 2H), 1.50 – 1.44 (m, 2H), 0.98 (t, 3H). ^13^C-NMR (101 MHz, DMSO) δ 164.53, 160.49, 139.90, 138.95, 138.13, 133.64, 132.11, 131.03, 121.63, 121.45, 66.04, 54.47, 30.60, 19.18, 14.61, 14.01. UPLC-MS: rt. 1.15 min, 335.00 m/z

**T-139**: 6-(butoxycarbonyl)-3-(ethoxycarbonyl)-2-methylquinoxaline 1,4-dioxide. 2.03 % yield dark red think oil. FT IR (ν cm^−1^): 2900-3000 (ArC-H); 2900-2800 (C-H); 1331 (*N-*oxide); 1745, 1719 (C=O); 1160-1300 ((C=O)-C- O). ^1^H NMR (400 MHz, DMSO) δ 8.94 – 8.88 (m, 1H), 8.56 (dd, *J* = 17.9, 8.9 Hz, 1H), 8.37 (dd, *J* = 8.9, 1.8 Hz, 1H), 4.53 (q, *J* = 7.1 Hz, 2H), 4.40 (t, *J* =6.5 Hz, 2H), 2.46 (s, 3H), 1.79 – 1.74 (m, 2H), 1.49 – 1.44 (m, 2H), 1.38 (t, *J* = 7.1 Hz, 3H), 0.97 (t, *J* = 7.4 Hz, 3H). ^13^C-NMR (126 MHz, CDCl_3_) δ 164.33, 159.57, 134.42, 133.45, 132.45, 131.36, 122.54, 122.32, 121.00, 120.79, 66.22, 63.88, 30.64, 19.23, 14.44, 14.01, 13.73. UPLC-MS: rt. 1.17 min, 348.06 m/z

**T-140**: 6-(butoxycarbonyl)-2-(tert-butoxycarbonyl)-3-methylquinoxaline 1,4-dioxide. 1.25 % yield, dark red think oil. FT IR (ν cm^−1^): 2900-3000 (ArC-H); 2900-2800 (C-H); 1724 (C=O); 1335 (*N-*oxide); 1160-1300 ((C=O)-C-O). ^1^H NMR (400 MHz, DMSO) δ 8.93 (d, *J* = 1.7 Hz, 1H), 8.55 – 8.52 (m, 1H), 8.36 (dd, *J* = 8.9, 1.8 Hz, 1H), 4.40 (t, *J* = 6.5 Hz, 2H), 2.48 (s, 3H), 1.80 – 1.74 (m, 2H), 1.62 (s, 9H), 1.51 – 1.44 (m, 2H), 0.97 (t, *J* = 7.4 Hz, 3H). ^13^C-NMR (101 MHz, DMSO) δ 164.56, 158.83, 139.39, 139.02, 138.02, 136.90, 133.49 130.99, 121.67, 121.41, 86.28, 66.05, 30.60, 28.06 (3C), 19.17, 14.30, 14.01. UPLC-MS: rt. 1.68 min, 377.11 m/z.

**T-141**: 3-((benzyloxy)carbonyl)-6-(butoxycarbonyl)-2-methylquinoxaline 1,4-dioxide. 1.38 % yield, dark red think oil. FT IR (ν cm^−1^): 2900-3000 (ArC-H); 2900-2800 (C-H); (*N-*oxide); 1749, 1719 (C=O); 1331; 1300-1160 ((C=O)-C-O). ^1^H NMR (400 MHz, DMSO) δ 8.93 (d, *J* = 1.8 Hz, 1H), 8.55 (d, *J* = 8.9 Hz, 1H), 8.37 (dd, *J* = 9.0, 1.8 Hz, 1H), 7.55 – 7.51 (m, 2H), 7.46 – 7.36 (m, 3H), 5.56 (s, 2H), 4.40 (t, *J* = 6.5 Hz, 2H), 2.42 (s, 3H), 1.78 (d, *J* = 4.5 Hz, 2H), 1.61 – 1.37 (m, 2H), 0.97 (t, *J* = 7.4 Hz, 3H). ^13^C-NMR (101 MHz, DMSO) δ 13.74, 14.26, 19.24, 30.64, 66.22, 69.34, 121.03, 122.31. 128.81, 128.87, 128.91, 129.16, 130.89, 131.34, 132.46, 133.98, 134.43, 137.79, 138.72, 139.77, 159.47, 164.32. UPLC-MS: rt. 1.76 min, 411.11 m/z.

**T-142**: 3-benzoyl-6-(butoxycarbonyl)-2-methylquinoxaline 1,4-dioxide. 2.48 % yield, light-yellow solid (124-126 °C). FT IR (ν cm^−1^): 2900-3000 (ArC-H); 2900-2800 (C-H); 1721, 1674 (C=O); 1325 (*N-*oxide); 1160-1300 ((C=O)-C-O). ^1^H NMR (400 MHz, DMSO) δ 9.04 – 8.99 (m, 1H), 8.52 (dd, *J* = 8.9, 0.6 Hz, 1H), 8.38 (dd, *J* = 9.0, 1.8 Hz, 1H), 8.12 – 8.05 (m, 2H), 7.79 (ddt, *J* = 8.7, 7.1, 1.3 Hz, 1H), 7.65 – 7.51 (m, 2H), 4.43 (t, *J* = 6.5 Hz, 2H), 2.34 (s, 3H), 1.85 – 1.70 (m, 2H), 1.55 – 1.41 (m, 2H), 0.98 (t, *J* = 7.4 Hz, 3H). ^13^C-NMR (101 MHz, DMSO) δ 187.43, 164.65, 140.20, 140.01, 139.29, 138.45, 135.93, 134.55, 133.44, 130.85, 129.95, 129.91, 121.68, 121.29, 66.08, 30.61, 19.19, 14.30, 14.03. UPLC-MS: rt. 1.45 min, 381.06 m/z.

**T-143**: 6-(butoxycarbonyl)-3-((4-chlorophenyl)carbamoyl)-2-methylquinoxaline 1,4-dioxide. 4.39 % yield, light-yellow solid (123-125 °C). FT IR (ν cm^−1^): 3223.86 (N-H), 2900-3000 (ArC-H), 2900-2800 (C-H), 1720, 1676 (C=O); 1342 (*N-*oxide), 1160-1300 ((C=O)-C-O); 600-830 (ArC-Cl). ^1^H NMR (400 MHz, DMSO) δ 11.12 (s, 1H), 9.01 (d, *J* = 1.8 Hz, 1H), 8.62 (d, *J* = 9.0 Hz, 1H), 8.42 (dd, *J* = 8.9, 1.8 Hz, 1H), 7.71 (d, *J* = 8.9 Hz, 2H), 7.51 (d, *J* = 10.8 Hz, 2H), 4.42 (t, *J* = 6.6 Hz, 3H), 2.53 (s, 3H), 1.81–1.74 (m, 2H), 1.49–1.42 (m, 2H), 0.98 (t, *J* = 7.4 Hz, 3H). ^13^C-NMR (101 MHz, DMSO) δ 163.64, 156.02, 143.21, 138.47, 138.04, 135.56, 135.05, 134.20, 132.21, 130.53, 129.03, 121.28, 120.84, 120.78, 66.48, 30.68, 19.23, 15.09, 13.78. UPLC-MS: rt. 1.38 min, 430.04 m/z.

**T-144**: 6-(butoxycarbonyl)-3-((2,4-dimethylphenyl)carbamoyl)-2-methylquinoxaline 1,4-dioxide. 2.68 % yield, yellow solid (124-126 °C). FT IR (ν cm^−1^): 3362.94 (N-H); 2900-3000 (ArC-H); 2900-2800 (C-H); 1720, 1695 (C=O); 1318 (*N-*oxide); 1160-1300 ((C=O)-C-O). ^1^H NMR (400 MHz, DMSO) δ 10.24 (s, 1H), 9.01 (d, *J* = 1.8 Hz, 1H), 8.64 (d, *J* = 9.0 Hz, 1H), 8.41 (dd, *J* = 8.9, 1.8 Hz, 1H), 7.51 (d, *J* = 8.0 Hz, 1H), 7.12 (s, 1H), 7.08 (dt, *J* = 8.3, 2.5 Hz, 1H), 4.41 (q, *J* = 6.8 Hz, 2H), 2.58 (s, 3H), 2.31 (s, 3H), 2.28 (s, 3H), 1.82 – 1.75 (m, 2H), 1.55 – 1.44 (m, 2H), 0.98 (t, *J* = 7.4 Hz, 3H). ^13^C-NMR (101 MHz, DMSO-d6) δ 164.58, 157.84, 139.70, 139.03, 137.55, 135.93, 133.52, 132.49, 132.11, 131.62, 131.07, 130.40, 127.25, 125.09, 121.68, 121.62, 66.08, 30.66, 20.98, 19.19, 18.17, 14.73, 14.02. UPLC-MS: rt. 1.30 min, 423.14 m/z.

**T-145**: 3-acetyl-6-(butoxycarbonyl)-2-(trifluoromethyl)quinoxaline 1,4-dioxide. 6.72 % yield, dark yellow solid (138-139 °C). FT IR (ν cm^−1^): 2900-3000 (ArC-H); 2900-2800 (C-H); 1726, 1714 (C=O); 1339 (*N-*oxide); 1160-1300 ((C=O)-C-O) 1176-1234 (C-F). ^1^H NMR (400 MHz, DMSO) δ 8.93 (d, *J* = 1.7 Hz, 1H), 8.59 (dd, *J* = 8.9, 0.6 Hz, 1H), 8.52 (dd, *J* = 8.9, 1.8 Hz, 1H), 4.42 (t, *J* = 6.5 Hz, 2H), 2.63 (s, 3H), 1.84 – 1.73 (m, 2H), 1.55 – 1.41 (m, 2H), 0.98 (t, *J* = 7.4 Hz, 3H). ^13^C-NMR (101 MHz, DMSO) δ 191.77, 164.21, 140.81, 140.14, 138.97, 134.14, 133.46, 129.48, 121.65, 121.42, 120.86-118.14, 66.28, 30.56, 29.64, 19.18, 14.01. UPLC-MS: rt. 1.99 min, 373.11 m/z.

**T-146**: 6-(butoxycarbonyl)-3-(ethoxycarbonyl)-2-(trifluoromethyl)quinoxaline 1,4-dioxide. 6.21 % yield, light-yellow solid (117-118 °C). FT IR (ν cm^−1^): 2900-3000 (ArC-H); 2900-2800 (C-H); 1744, 1729 (C=O); 1347 (*N-*oxide); 1160-1300 ((C=O)-C-O), 1156-1225 (C-F). ^1^H NMR (400 MHz, DMSO) δ 8.90 (d, *J* = 1.7 Hz, 1H), 8.58 (dd, *J* = 9.0, 0.6 Hz, 1H), 8.50 (dd, *J* = 9.0, 1.8 Hz, 1H), 4.51 (q, *J* = 7.1 Hz, 2H), 4.42 (t, *J* = 6.5 Hz, 2H), 1.84 – 1.72 (m, 2H), 1.55 – 1.41 (m, 2H), 1.36 (t, *J* = 7.1 Hz, 3H), 0.98 (t, *J* = 7.4 Hz, 3H). ^13^C-NMR (101 MHz, DMSO) δ 164.20, 158.13, 140.87, 139.39, 135.03, 134.25, 133.50, 121.74, 121.65, 120.7-117.97, 66.27, 64.27, 30.56, 19.17, 14.07, 14.00. UPLC-MS: rt. 1.84 min, 403.09 m/z.

**T-148**: 3-benzoyl-6-(butoxycarbonyl)-2-(trifluoromethyl)quinoxaline 1,4-dioxide. 19.02 % yield, light-yellow solid (162-164 °C). FT IR (ν cm^−1^): 2900-3000 (ArC-H); 2900-2800 (C-H); 1724, 1689 (C=O); 1339 (*N-*oxide); 1300-1160 ((C=O)-C-O); 1228-1135 (C-F). ^1^H NMR (400 MHz, DMSO) δ 8.98 (dd, *J* = 1.6, 0.8 Hz, 1H), 8.58 – 8.48 (m, 2H), 8.19 – 8.11 (m, 2H), 7.84 – 7.75 (m, 1H), 7.66 – 7.58 (m, 2H), 4.44 (t, *J* = 6.5 Hz, 2H), 1.79 (dq, *J* = 7.9, 6.6 Hz, 2H), 1.56 – 1.42 (m, 2H), 0.98 (t, *J* = 7.4 Hz, 3H). ^13^C-NMR (101 MHz, DMSO) δ 184.53, 164.28, 141.21, 139.69, 138.97, 135.90, 134.40, 134.15, 133.38, 130.52-130.18, 129.90, 129.71, 121.76, 121.50, 120.93-118.22, 66.29, 30.58, 19.18, 14.02. UPLC-MS: rt. 2.13 min, 435.05 m/z.

**T-149**: 6-(butoxycarbonyl)-3-(thiophene-2-carbonyl)-2-(trifluoromethyl)quinoxaline 1,4-dioxide. 20.58 % yield, light-yellow solid (164-166 °C). FT IR (ν cm^−1^): 2900-3000 (ArC-H); 2900-2800 (C-H); 1721, 1661 (C=O); 1330 (*N-*oxide); 1300-1160 ((C=O)-C-O); 1227-1134 (C-F); 700-600 (ArC-S). ^1^H NMR (400 MHz, DMSO) δ 8.98 (d, *J* = 1.6 Hz, 1H), 8.60 – 8.49 (m, 2H), 8.29 (dd, *J* = 4.9, 1.2 Hz, 1H), 8.23 (dd, *J* = 3.9, 1.2 Hz, 1H), 7.32 (dd, *J* = 4.9, 3.9 Hz, 1H), 4.44 (t, *J* = 6.5 Hz, 2H), 1.85 – 1.74 (m, 2H), 1.56 – 1.42 (m, 2H), 0.98 (t, *J* = 7.4 Hz, 3H). ^13^C-NMR (101 MHz, DMSO) δ 176.30, 164.26, 141.29, 141.24, 139.61, 139.02, 138.43, 138.37, 134.20, 133.30, 129.90, 121.73, 121.64, 120.86-118.15, 66.29, 30.57, 19.18, 14.02. UPLC-MS: rt. 1.83 min, 441.02 m/z.

**T-150**: 3-(2-naphthoyl)-6-(butoxycarbonyl)-2-(trifluoromethyl)quinoxaline 1,4-dioxide. 1.17 % yield, light-yellow solid (165-167 °C). FT IR (ν cm^−1^): 2900-3000 (ArC-H); 2900-2800 (C-H); 1726, 1675 (C=O); 1338 (*N-*oxide); 1300-1160 ((C=O)-C-O); 1259-1156 (C-F). ^1^H NMR (400 MHz, DMSO) δ 9.03 (dd, *J* = 1.7, 0.7 Hz, 1H), 8.85 (d, *J* = 1.1 Hz, 1H), 8.61 – 8.50 (m, 2H), 8.14 (d, *J* = 1.3 Hz, 2H), 8.09 – 8.01 (m, 2H), 7.75 (ddd, *J* = 8.3, 6.9, 1.3 Hz, 1H), 7.66 (ddd, *J* = 8.1, 6.9, 1.3 Hz, 1H), 4.45 (t, *J* = 6.5 Hz, 2H), 1.84 – 1.76 (m, 2H), 1.52 – 1.46 (m, 2H), 0.99 (t, *J* = 7.4 Hz, 3H) ^13^C-NMR (101 MHz, DMSO-d6) δ 184.40, 164.28, 141.27, 139.65, 139.09, 136.51, 134.21, 133.14, 132.82, 131.89, 131.32, 130.11, 129.71, 128.45, 127.95, 123.45, 121.80, 121.63, 120.93-118.22, 66.32, 30.58, 19.19, 14.02. UPLC-MS: rt. 2.60 min, 484.33 m/z.

**T-151**: 6-(butoxycarbonyl)-2-phenyl-3-(phenylcarbamoyl)quinoxaline 1,4-dioxide. 21.88 % yield, yellow solid (118-120 °C). FT IR (ν cm^−1^): 3297.34 (N-H); 2900-3000 (ArC-H); 2900-2800 (C-H); 1719, 1666 (C=O); 1338 (*N-*oxide); 1160-1300 ((C=O)-C-O). ^1^H NMR (400 MHz, DMSO) δ 10.77 (s, 1H), 9.04 (dd, *J* = 3.0, 1.7 Hz, 1H), 8.69 (dd, *J* = 9.0, 2.1 Hz, 1H), 8.49 (dt, *J* = 8.9, 1.7 Hz, 1H), 7.67 – 7.61 (m, 2H), 7.52 – 7.46 (m, 4H), 7.38 (dt, *J* = 8.3, 1.5 Hz, 2H), 7.34 – 7.28 (m, 2H), 4.43 (td, *J* = 6.5, 3.6 Hz, 2H), 1.85 – 1.73 (m, 2H), 1.49 (hd, *J* = 7.4, 2.2 Hz, 2H), 0.98 (td, *J* = 7.4, 2.3 Hz, 3H). ^13^C NMR (101 MHz, DMSO) δ 164.54, 156.70, 140.94, 140.30, 139.81, 139.51, 138.26, 137.97, 133.74, 133.42, 132.21, 131.85, 130.80, 130.25, 129.47, 128.71, 128.42, 125.06, 122.28, 121.72, 119.96, 66.12, 30.61, 19.20, 14.03. UPLC-MS: rt. 1.30 min, 458.16 m/z.

**T-155**. 3-acetyl-6-(isobutoxycarbonyl)-2-methylquinoxaline 1,4-dioxide. 13.4 % yield, yellow solid (95-97 °C). FT IR (ν cm^−1^): 2962 (ArC-H), 1716 (C=O); 1328 (*N-*oxide). ^1^H NMR (400 MHz, DMSO) δ 8.96 (dd, *J* = 1.8, 0.5 Hz, 1H), 8.56 (dd, *J* = 8.9, 0.6 Hz, 1H), 8.38 (dd, *J* = 9.0, 1.8 Hz, 1H), 4.20 (d, *J* = 6.6 Hz, 2H), 2.67 (s, 3H), 2.40 (s, 3H), 2.12 (hept, *J* = 6.7 Hz, 1H), 1.03 (d, *J* = 6.8 Hz, 6H). ^13^C-NMR (101 MHz, DMSO) δ 195.61, 164.52, 141.16, 139.75, 138.91, 137.87, 133.45, 130.92, 121.58, 121.26, 71.96, 29.96, 27.83, 19.34, 14.00. UPLC-MS: rt. 1.73 min, 319.56 m/z.

**T-156**. 6-(isobutoxycarbonyl)-3-(methoxycarbonyl)-2-methylquinoxaline 1,4-dioxide. 4.3 % yield, yellow solid (115-117 °C). FT IR (ν cm^−1^): 2959-3099 (ArC-H), 1744, 1718 (C=O), 1327 (*N-*oxide). ^1^H NMR (400 MHz, DMSO) δ 8.96 – 8.87 (m, 1H), 8.56 (dd, *J* = 17.9, 9.0 Hz, 1H), 8.40 (ddd, *J* = 13.9, 8.9, 1.8 Hz, 1H), 4.20 (dd, *J* = 6.5, 2.0 Hz, 2H), 4.04 (s, 3H), 2.46 (d, *J* = 2.6 Hz, 3H), 2.11 (dpd, *J* = 13.3, 6.7, 2.6 Hz, 1H), 1.02 (dd, *J* = 6.7, 1.4 Hz, 6H). ^13^C-NMR (101 MHz, DMSO) δ 164.48, 160.46, 139.90, 138.15, 136.50, 132.65, 132.10, 131.02, 121.63, 121.44, 71.97, 54.47, 27.83, 19.32, 14.61. UPLC-MS: rt. 1.71 min, 335.54 m/z.

**T-157**. 3-benzoyl-6-(isobutoxycarbonyl)-2-methylquinoxaline 1,4-dioxide. 20 % yield, light-yellow solid (130-132 °C). FT IR (ν cm^−1^): 2970-3101 (ArC-H), 1726, 1675 (C=O), 1322 (*N-*oxide). ^1^H NMR (400 MHz, DMSO) δ 9.03 (d, *J* = 1.8 Hz, 1H), 8.52 (d, *J* = 8.9 Hz, 1H), 8.40 (dd, *J* = 8.9, 1.8 Hz, 1H), 8.12 – 8.05 (m, 2H), 7.83 – 7.74 (m, 1H), 7.65 – 7.55 (m, 2H), 4.23 (d, *J* = 6.5 Hz, 2H), 2.34 (s, 3H), 2.13 (hept, *J* = 6.7 Hz, 1H), 1.04 (d, *J* = 6.7 Hz, 6H). ^13^C-NMR (101 MHz, DMSO) δ 187.42, 164.60, 140.21, 140.01, 139.31, 138.47, 135.92, 134.55, 133.40, 130.84, 129.95, 129.90, 121.68, 121.34, 71.96, 27.85, 19.34, 14.30. UPLC-MS: rt. 1.77 min, 381.13 m/z.

**T-158**. 6-(isobutoxycarbonyl)-2-methyl-3-(phenylcarbamoyl)quinoxaline 1,4-dioxide. 22.5 % yield, yellow solid (135-137 °C). FT IR (ν cm^−1^): 3260 (N-H,), 2960-2874 (ArC-H), 1720, 1677 (C=O), 1375 (*N-*oxide). ^1^H NMR (400 MHz, DMSO) δ 10.96 (s, 1H), 9.02 (d, *J* = 1.8 Hz, 1H), 8.63 (d, *J* = 9.0 Hz, 1H), 8.43 (dd, *J* = 9.0, 1.8 Hz, 1H), 7.72 – 7.64 (m, 2H), 7.43 (t, *J* = 7.9 Hz, 2H), 7.23 – 7.18 (m, 1H), 4.22 (d, *J* = 6.5 Hz, 2H), 2.54 (s, 3H), 2.13 (hept, *J* = 6.6 Hz, 1H), 1.04 (d, *J* = 6.7 Hz, 6H). ^13^C-NMR (101 MHz, DMSO) δ 164.52, 157.30, 140.49, 139.32, 138.99, 138.27, 137.63, 133.59, 131.16, 129.63, 125.24, 121.72, 121.65, 120.10, 72, 27.84, 19.36, 14.72. UPLC-MS: rt. 1.80 min, 396.58 m/z.

**T-159**. 3-((2,4-dimethylphenyl)carbamoyl)-6-(isobutoxycarbonyl)-2-methylquinoxaline 1,4-dioxide. 3.7 % yield, yellow solid (128-131 °C). FT IR (ν cm^−1^): 3349 (N-H), 2957-2873 (ArC-H), 1717, 1695 (C=O), 1374 (*N-*oxide). ^1^H NMR (500 MHz, CDCl_3_) δ 10.75 (s, 1H), 9.26 (d, *J* = 1.7 Hz, 1H), 8.71 (d, *J* = 9.0 Hz, 1H), 8.47 (dd, *J* = 9.0, 1.8 Hz, 1H), 7.88 (d, *J* = 7.9 Hz, 1H), 7.09 (d, *J* = 7.9 Hz, 2H), 4.25 (d, *J* = 6.7 Hz, 2H), 2.97 (s, 3H), 2.44 (s, 3H), 2.35 (s, 3H), 2.23 – 2.13 (m, 1H), 1.09 (d, *J* = 6.7 Hz, 6H). ^13^C-NMR (126 MHz, CDCl_3_) δ 164.23, 159.91, 144.50, 138.11, 137.37, 137.15, 135.92, 134.39, 132.20, 131.65, 131.41, 129.71, 127.34, 123.11, 122.35, 121.23, 72.34, 27.87, 20.99, 19.19, 18.12, 15.84.

**T-161**. 3-((4-chlorophenyl)carbamoyl)-6-(isobutoxycarbonyl)-2-methylquinoxaline 1,4-dioxide. 14 % yield, yellow solid (130-132 °C). FT IR (ν cm^−1^): 3245-3188 (N-H), 2965-2873 (ArC-H), 1722, 1685 (C=O), 1342 (*N-*oxide), 829 (Ar-Cl). ^1^H NMR (400 MHz, DMSO) δ 11.12 (s, 1H), 9.02 (d, *J* = 1.7 Hz, 1H), 8.63 (d, *J* = 9.0 Hz, 1H), 8.43 (dd, *J* = 8.9, 1.8 Hz, 1H), 7.75 – 7.67 (m, 2H), 7.50 – 7.46 (m, 2H), 4.21 (d, *J* = 6.6 Hz, 2H), 2.53 (s, 3H), 2.12 (hept, *J* = 6.7 Hz, 1H), 1.04 (d, *J* = 6.7 Hz, 6H). ^13^C-NMR (101 MHz, DMSO) δ 164.50, 157.41, 140.47, 139.11, 138.96, 137.68, 137.18, 133.64, 131.20, 129.58, 128.95, 121.73, 121.71, 121.66, 72, 27.84, 19.36, 14.71. UPLC-MS: rt. 1.70 min, 430.12 m/z.

**T-163**. 3-benzoyl-6-(isobutoxycarbonyl)-2-(trifluoromethyl)quinoxaline 1,4-dioxide. 2.7 % yield, light-yellow solid (148-150 °C). . FT IR (ν cm^−1^): 2876-3095 (ArC-H), 1721, 1689 (C=O), 1338 (*N-*oxide), 1285-1132 (Ar-CF3). ^1^H NMR (400 MHz, DMSO) δ 8.99 (s, 1H), 8.54 (s, 1H), 8.15 (d, *J* = 7.2 Hz, 1H), 7.88 (d, *J* = 7.8 Hz, 2H), 7.78 (d, *J* = 7.5 Hz, 1H), 7.62 (t, *J* = 7.7 Hz, 2H), 4.24 (d, *J* = 6.5 Hz, 2H), 2.13 (dp, *J* = 13.3, 6.6 Hz, 1H), 1.04 (d, *J* = 6.7 Hz, 6H). ^13^C-NMR (101 MHz, DMSO) δ 184.52, 164.23, 141.25, 139.70, 135.90, 134.40, 134.11, 133.38, 129.90, 129.71, 128.96, 127.60, 121.74, 121.55, 72.15, 27.83, 19.32. UPLC-MS: rt. 3.072 min, 435.09 m/z.

**T-164**. 6-(isobutoxycarbonyl)-3-(thiophene-2-carbonyl)-2-(trifluoromethyl)quinoxaline 1,4-dioxide. 4.1 % yield, light yellow solid (167-168 °C). FT IR (ν cm^−1^): 2959 (ArC-H), 1720, 1657 (C=O), 1358 (*N-*oxide), 1155 (Ar-CF3). ^1^H NMR (400 MHz, DMSO) δ 8.99 (d, *J* = 1.7 Hz, 1H), 8.61 – 8.50 (m, 2H), 8.29 (dd, *J* = 4.9, 1.2 Hz, 1H), 8.22 (dd, *J* = 3.9, 1.2 Hz, 1H), 7.32 (t, *J* = 4.4 Hz, 1H), 4.24 (d, *J* = 6.5 Hz, 2H), 2.13 (hept, *J* = 6.6 Hz, 1H), 1.04 (d, *J* = 6.7 Hz, 6H). ^13^C-NMR (101 MHz, DMSO) δ 176.29, 164.22, 141.32, 141.24, 139.63, 139.02, 138.43, 134.16, 133.38, 129.92, 121.73, 121.69, 120.88-118.15, 72.15, 27.83, 19.32. UPLC-MS: rt. 2.66 min, 444.17 m/z.

**T-165**. 3-(furan-2-carbonyl)-6-(isobutoxycarbonyl)-2-(trifluoromethyl)quinoxaline 1,4-dioxide. 9.0 % yield, light-yellow solid (164-166 °C). FT IR (ν cm^−1^): 2962-2876 (ArC-H), 1722, 1665 (C=O), 1339 (*N-*oxide). ^1^H NMR (400 MHz, DMSO) δ 8.98 (d, *J* = 1.7 Hz, 1H), 8.56 (qd, *J* = 8.9, 1.2 Hz, 2H), 8.24 (dd, *J* = 1.7, 0.7 Hz, 1H), 7.89 (dd, *J* = 3.7, 0.8 Hz, 1H), 6.84 (dd, *J* = 3.7, 1.7 Hz, 1H), 4.23 (d, *J* = 6.5 Hz, 2H), 2.13 (hept, *J* = 6.7 Hz, 1H), 1.04 (d, *J* = 6.7 Hz, 6H). ^13^C-NMR (101 MHz, DMSO) δ 170.15, 164.19, 151.42, 150.47, 141.25, 139.61, 137.74, 134.25, 133.44, 130.27, 124.62, 121.75, 121.71, 120.88-118.15, 114.08, 72.16, 27.82, 19.31. UPLC-MS: rt. 2.41 min, 425.47 m/z.

**T-166**. 3-(2-naphthoyl)-6-(isobutoxycarbonyl)-2-(trifluoromethyl)quinoxaline 1,4-dioxide. 5.0 % yield, yellow solid (180-182 °C). FT IR (ν cm^−1^): 2875-3095 (ArC-H), 1722, 1687 (C=O), 1339 (*N-*oxide), 1170-1125 (Ar-CF3). ^1^H NMR (400 MHz, DMSO) δ 9.04 (s, 0H), 8.85 (d, *J* = 1.2 Hz, 1H), 8.62 – 8.52 (m, 2H), 8.14 (d, *J* = 1.3 Hz, 2H), 8.10 – 7.99 (m, 2H), 7.75 (ddd, *J* = 8.3, 6.9, 1.3 Hz, 1H), 7.66 (ddd, *J* = 8.2, 6.9, 1.3 Hz, 1H), 4.25 (d, *J* = 6.0 Hz, 1H), 2.13 (dh, *J* = 12.8, 6.4 Hz, 1H), 1.05 (d, *J* = 6.7 Hz, 6H). ^13^C-NMR (101 MHz, DMSO) δ 184.39, 164.24, 141.30, 139.66, 136.51, 134.18, 133.46, 133.15, 132.82, 131.89, 130.32, 130.11, 129.78, 128.47, 127.95, 123.45, 121.79, 121.68, 120.99-118.26, 72.16, 27.84, 19.32.

**T-167**. 3-acetyl-6-(isobutoxycarbonyl)-2-(trifluoromethyl)quinoxaline 1,4-dioxide. 4.8 % yield, yellow solid (102 °C). FT IR (ν cm^−1^): 2962-3093 (ArC-H), 1721 (C=O), 1334 (*N-*oxide), 1282-1152 (Ar-CF3). ^1^H NMR (400 MHz, DMSO) δ 8.96 – 8.91 (m, 1H), 8.60 (dd, *J* = 8.9, 0.6 Hz, 1H), 8.53 (dd, *J* = 9.0, 1.8 Hz, 1H), 4.22 (d, *J* = 6.5 Hz, 2H), 2.63 (s, 3H), 2.12 (hept, *J* = 6.7 Hz, 1H), 1.03 (d, *J* = 6.7 Hz, 6H). ^13^C-NMR (101 MHz, DMSO) δ 191.77, 164.16, 140.98, 140.14, 138.98, 134.10, 133.46, 129.15, 121.64, 121.46, 120.896-118.14, 72.15, 29.65, 27.81, 19.32. UPLC-MS: rt. 2.09 min, 373.08 m/z.

**T-168**. 2-benzoyl-6-(isobutoxycarbonyl)-3-methylquinoxaline 1,4-dioxide. 10.5 % yield, light-yellow solid (131-133 °C). FT IR (ν cm^−1^): 2874-3110 (ArC-H), 1718, 1685 (C=O), 1322 (*N-*oxide). ^1^H NMR (400 MHz, DMSO) δ 8.88 (d, *J* = 1.7 Hz, 1H), 8.66 (d, *J* = 9.0 Hz, 1H), 8.47 (dd, *J* = 4.0, 1.8 Hz, 1H), 8.13 – 8.05 (m, 2H), 7.83 – 7.74 (m, 1H), 7.64 – 7.56 (m, 2H), 4.21 (d, *J* = 6.5 Hz, 2H), 2.46 (s, 3H), 2.11 (dh, *J* = 13.3, 6.6 Hz, 1H), 1.01 (d, *J* = 5.4 Hz, 6H). ^13^C-NMR (101 MHz, DMSO) δ 187.40, 164.51, 141.75, 140.45, 138.53, 135.91, 133.52, 131.58, 130.39, 129.89, 128.97, 121.54, 121.35, 72.00, 30.26, 27.83, 19.32. UPLC-MS: rt. 1.65 min, 381.13 m/z.

**T-169**. 6-(isobutoxycarbonyl)-2-phenyl-3-(phenylcarbamoyl)quinoxaline 1,4-dioxide. 3.5 % yield, yellow solid (150-153 °C). FT IR (ν cm^−1^): 3198 (N-H), 2961 (ArC-H), 1723, 1685 (C=O), 1333 (*N-*oxide). ^1^H NMR (400 MHz, DMSO) δ 10.77 (d, *J* = 17.6 Hz, 1H), 9.06 (d, *J* = 1.9 Hz, 1H), 8.70 (d, *J* = 9.0 Hz, 1H), 8.50 (d, *J* = 8.9 Hz, 1H), 7.64 (dd, *J* = 6.5, 3.0 Hz, 2H), 7.51 – 7.47 (m, 3H), 7.39 (d, *J* = 8.0 Hz, 2H), 7.31 (t, *J* = 7.8 Hz, 2H), 7.11 (t, *J* = 7.2 Hz, 1H), 4.22 (d, *J* = 6.4 Hz, 2H), 2.12 (hept, *J* = 6.9 Hz, 1H), 1.04 (d, *J* = 6.8 Hz, 6H). ^13^C-NMR (101 MHz, DMSO) δ 164.49, 156.70, 140.96, 139.82, 139.53, 138.27, 137.97, 133.70, 131.84, 130.80, 130.26, 129.47, 128.71, 128.42, 125.06, 122.28, 121.76, 119.97, 72.02, 27.84, 19.35..

**T-170**. 2-((2,4-dimethylphenyl)carbamoyl)-6-(isobutoxycarbonyl)-3-methylquinoxaline 1,4-dioxide. 2.3 % yield, yellow solid (129-132 °C). FT IR (ν cm^−1^): 3103 (N-H), 2960-2872 (ArC-H), 1725, 1664 (C=O), 1335 (*N-*oxide). ^1^H NMR (500 MHz, CDCl_3_) δ 9.93 (s, 1H), 8.83 (d, *J* = 1.8 Hz, 1H), 8.49 – 8.38 (m, 1H), 8.18 (d-, *J* = 8.7 Hz, 1H), 8.04 (dd, *J* = 8.0, 5.3 Hz, 1H), 7.16 – 7.09 (m, 2H), 4.24 (d, *J* = 6.7 Hz, 2H), 3.26 (s, 3H), 2.44 (s, 3H), 2.36 (s, 3H), 2.19 (ddt, *J* = 13.4, 9.6, 6.7 Hz, 1H), 1.11 (dd, *J* = 6.7, 2.5 Hz, 6H). ^13^C-NMR (101 MHz, DMSO) δ 165.58, 161.67, 144.67, 142.41, 140.89, 134.96, 133.24, 132.95, 131.79, 131.29, 131.20, 130.89, 129.45, 128.79, 127.45, 121.95, 71.86, 27.93, 25.13, 20.96, 19.23, 17.78. UPLC-MS: rt. 1.67 min, 424.18 m/z.

T-137 IR


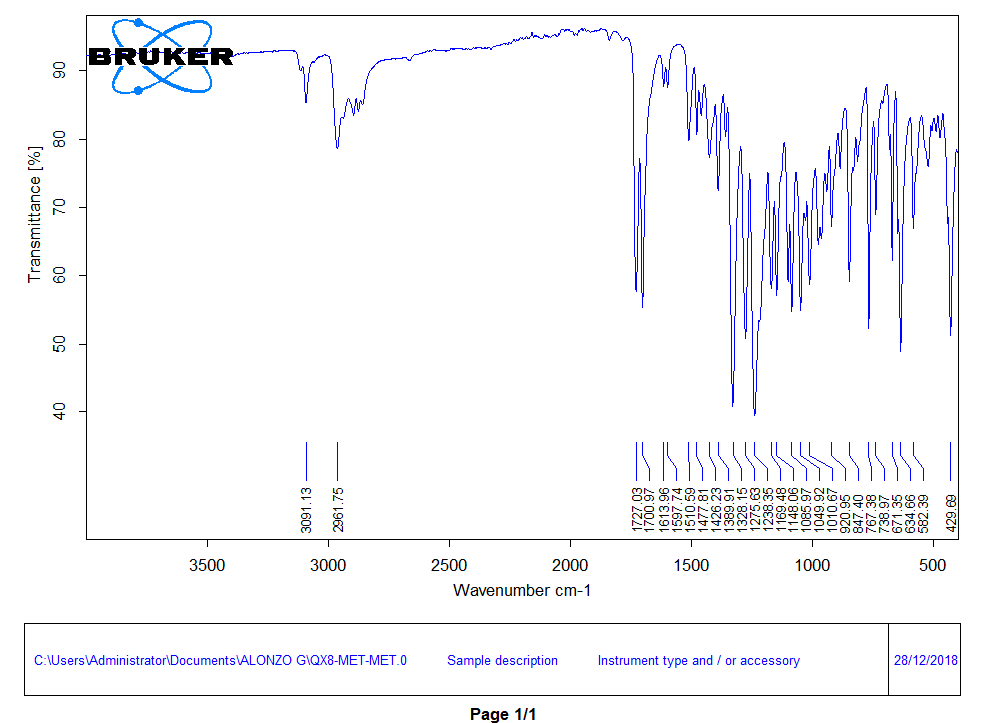


T-137 ^1^H-NMR


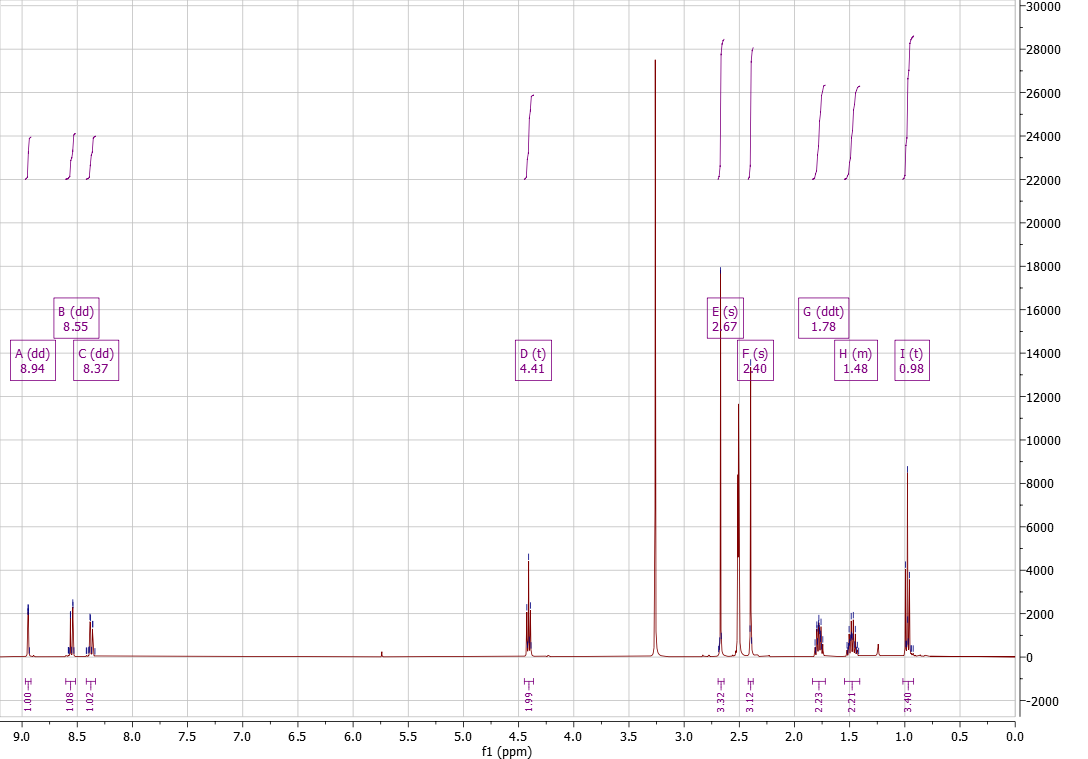


T-137 ^13^C-NMR


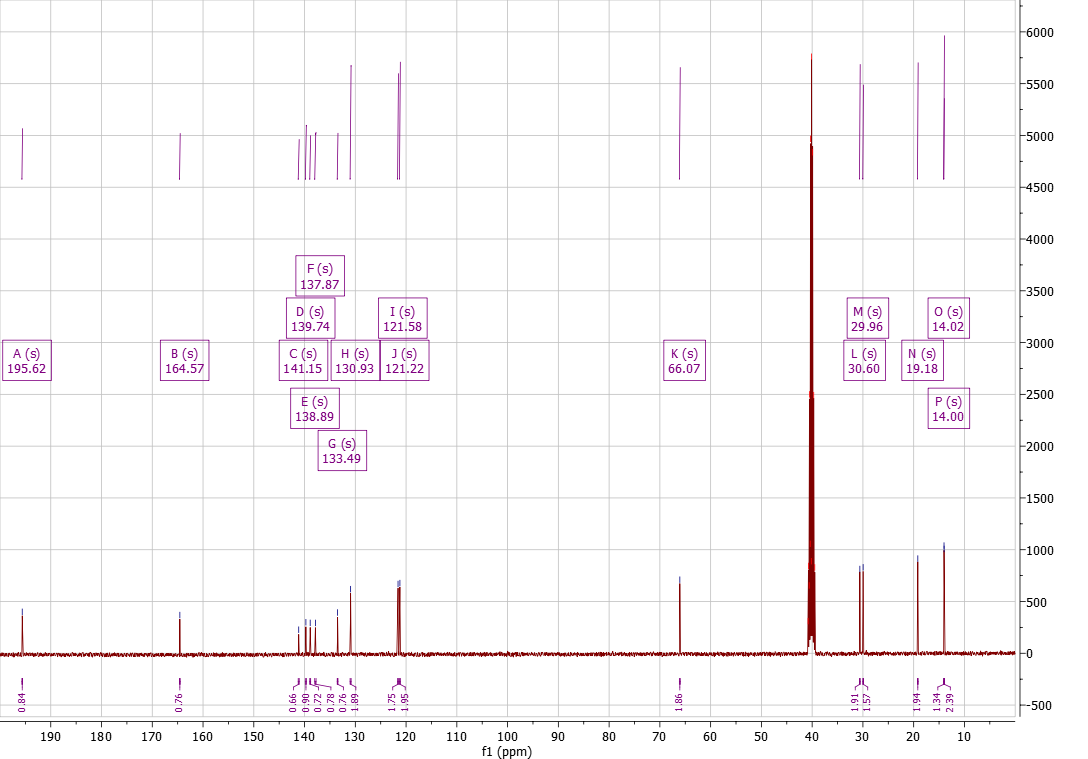


T-137 UPLC-MS


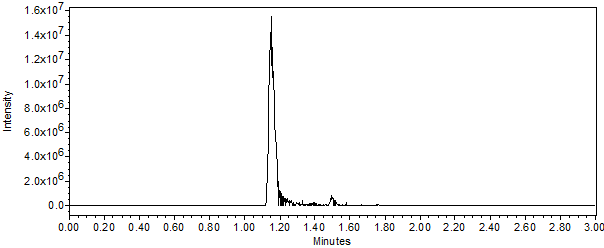


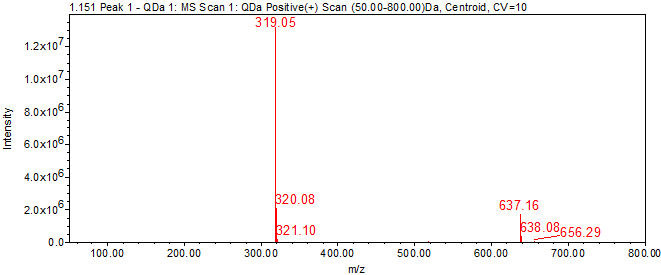


T-138 IR


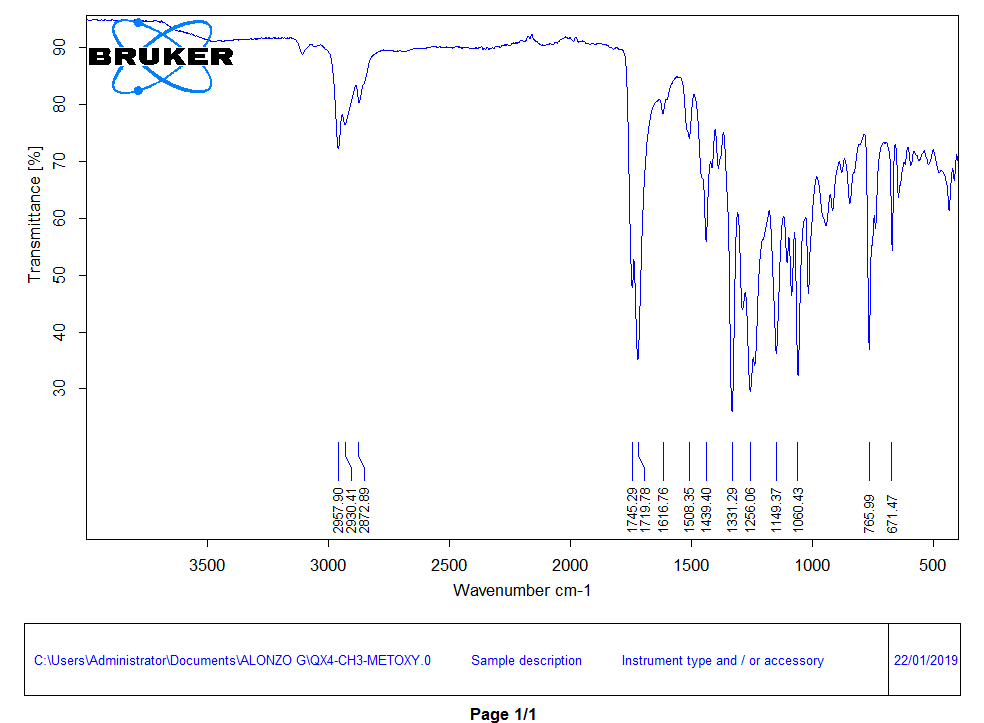


T-138 ^1^H-NMR


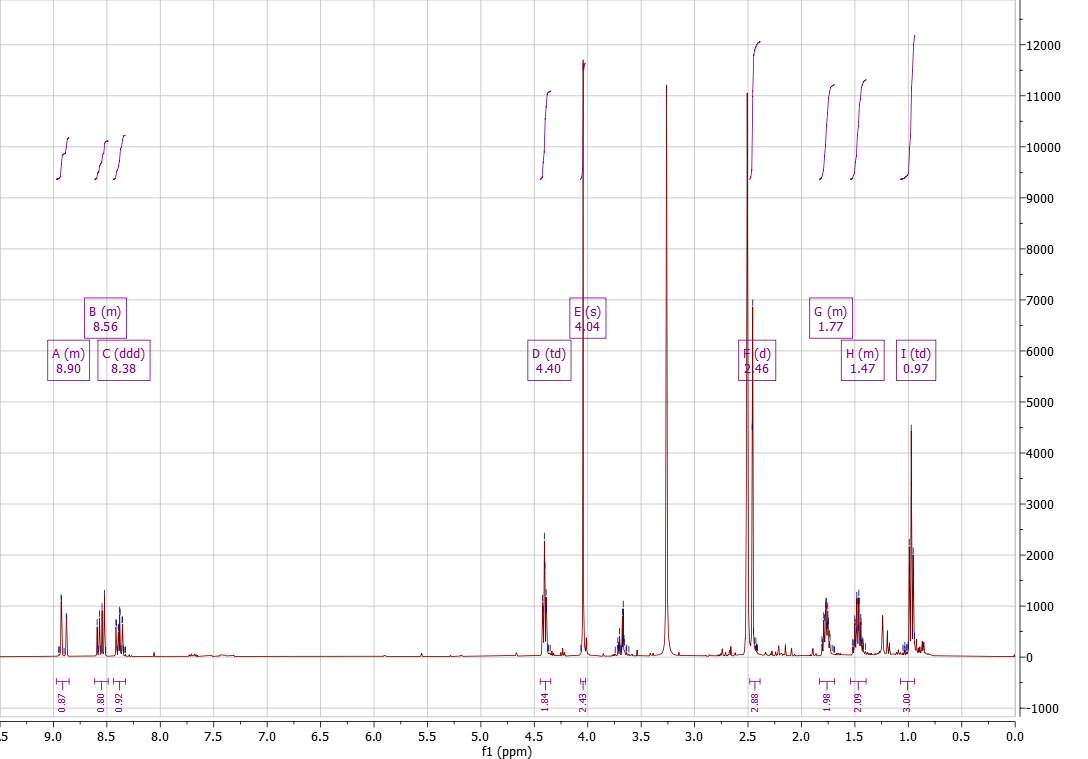


T-138 ^13^C-NMR


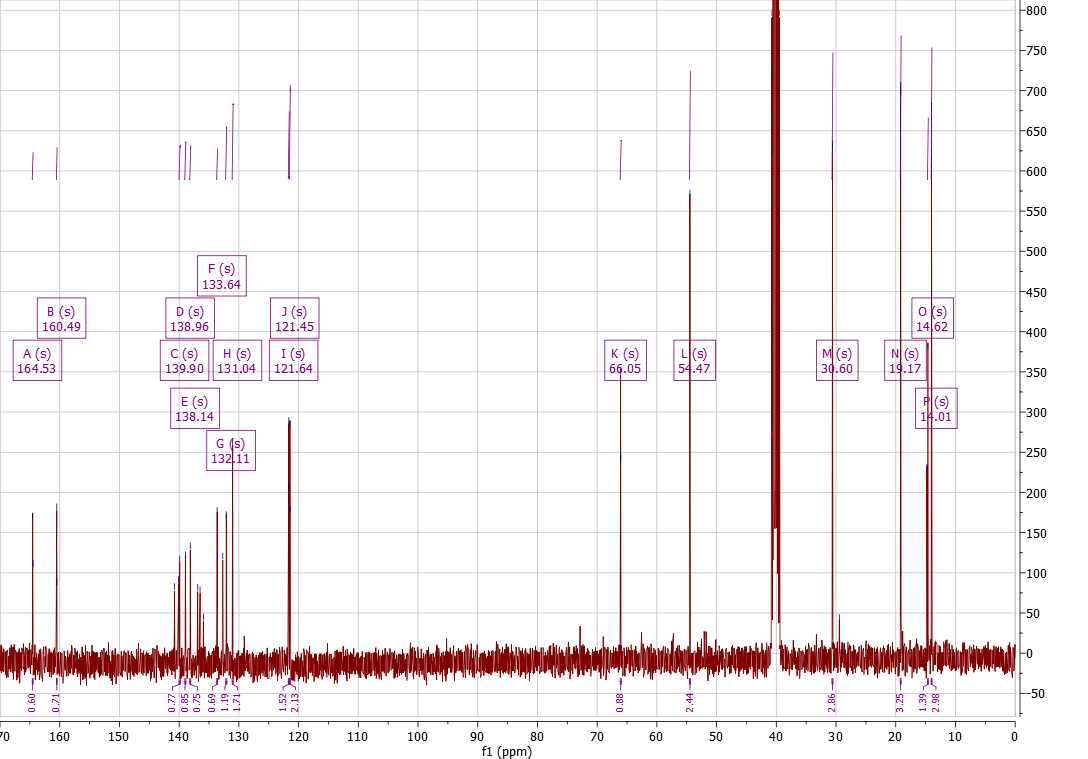


T-138 UPLC-MS


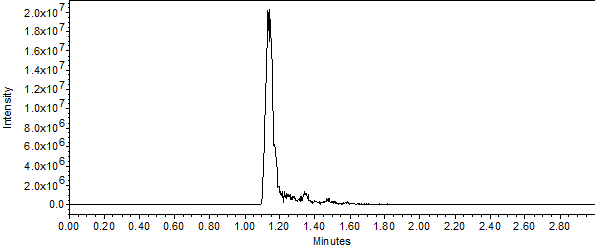


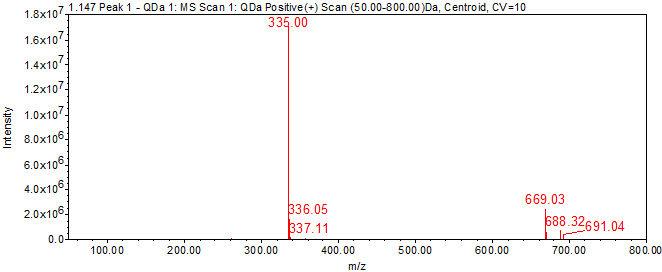


T-139 IR


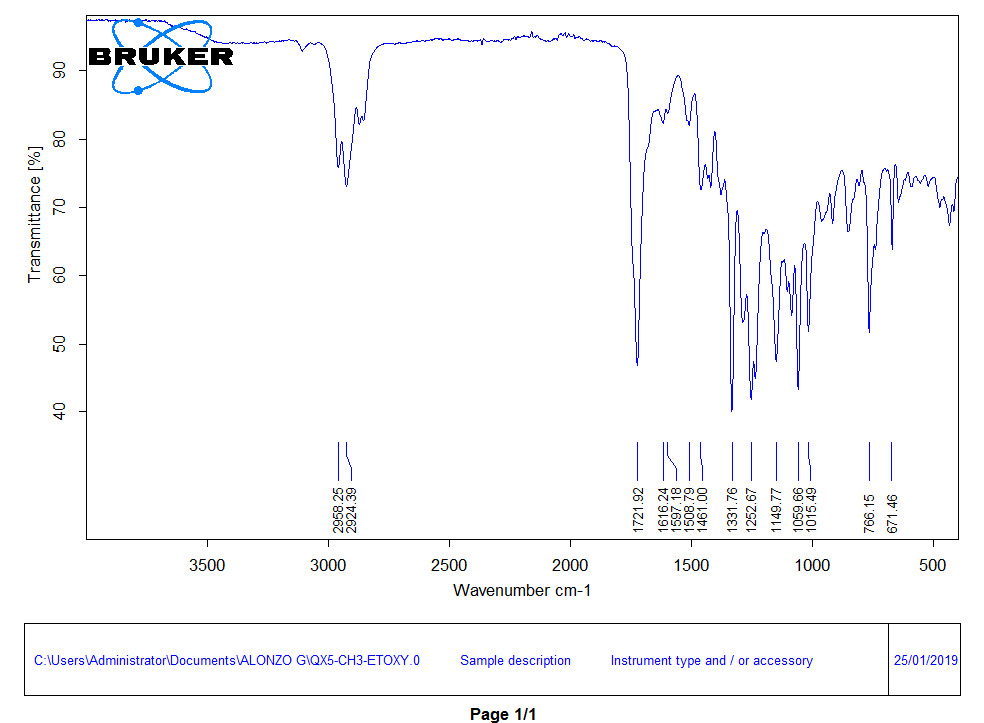


T-139 ^1^H-NMR


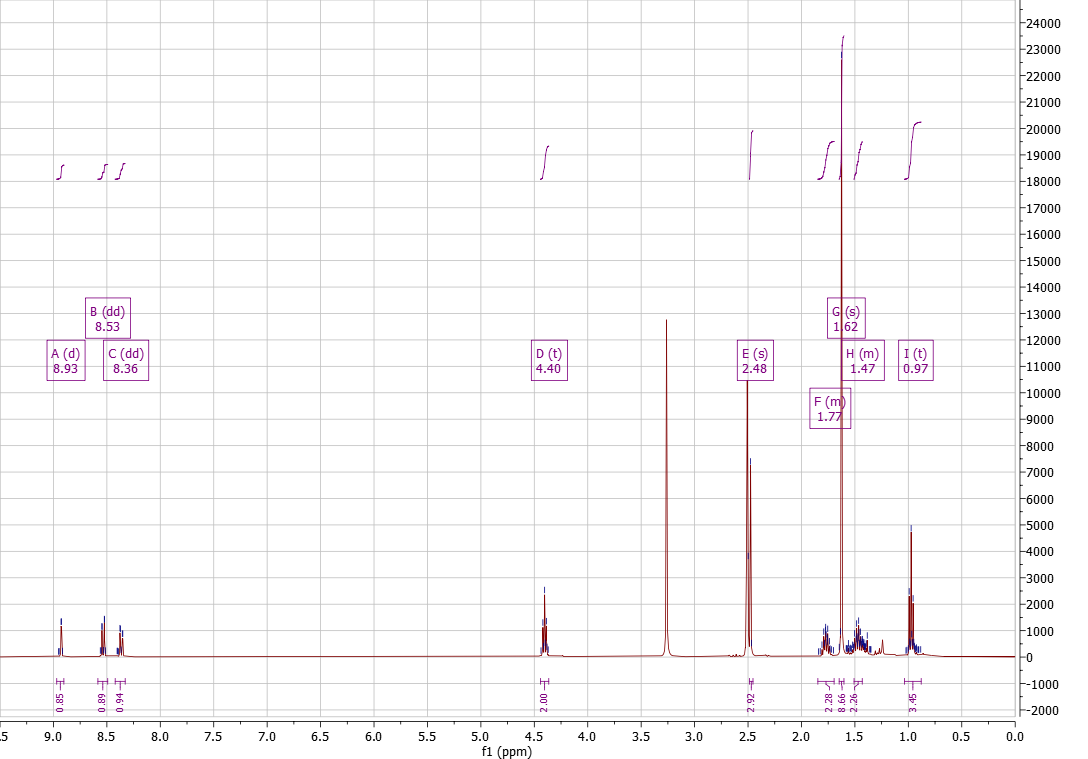


T-139 ^13^C-NMR


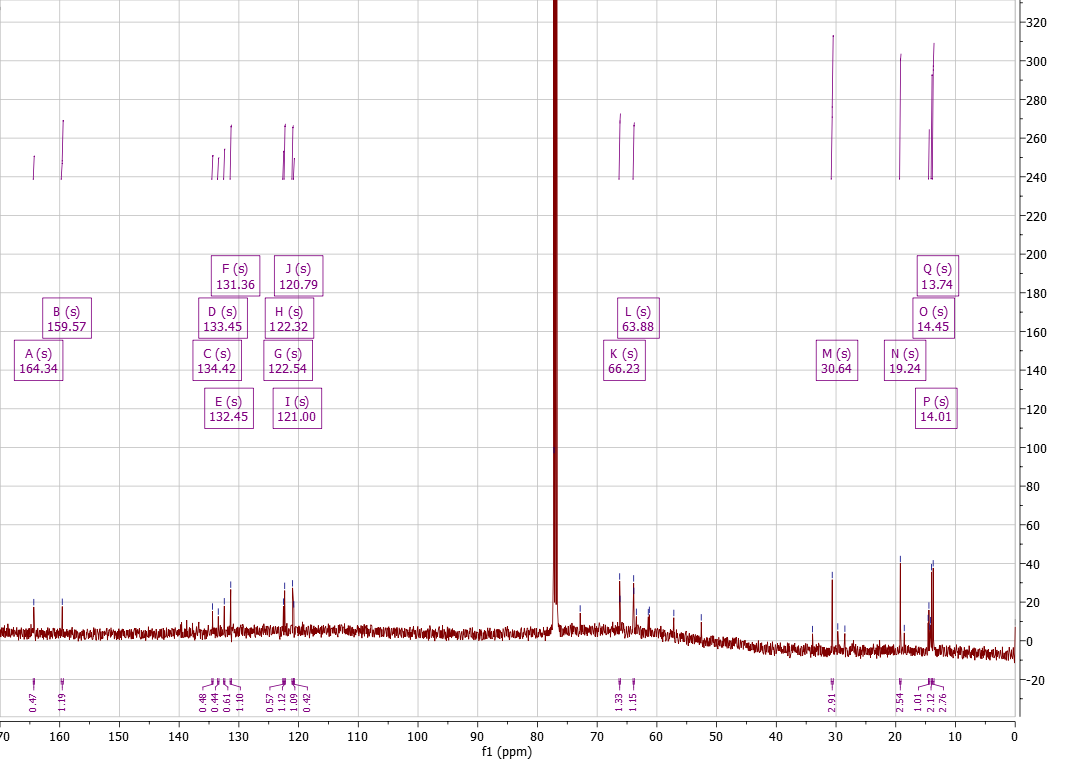


T-139 UPLC-MS


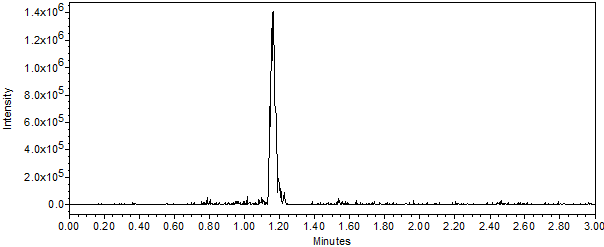


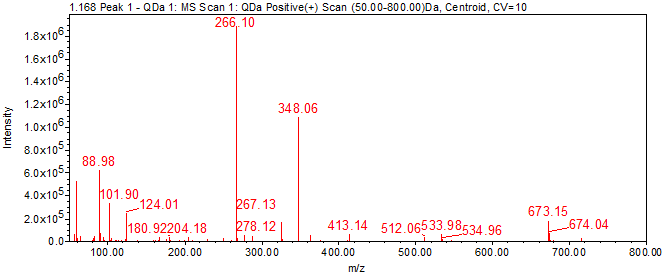


T-140 IR


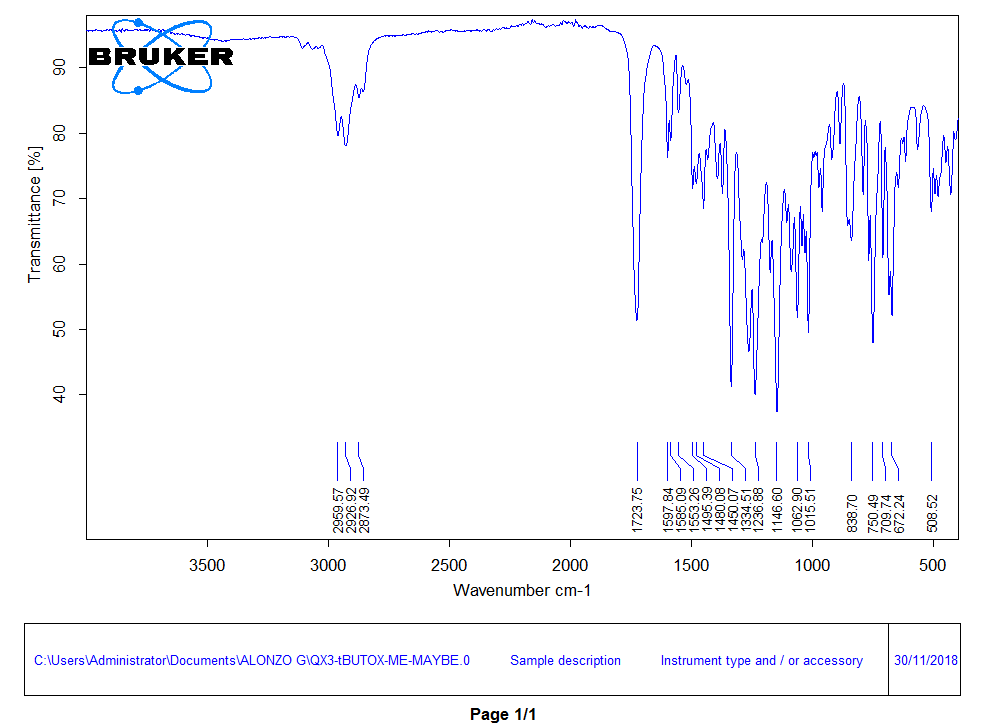


T-140 ^1^H-NMR


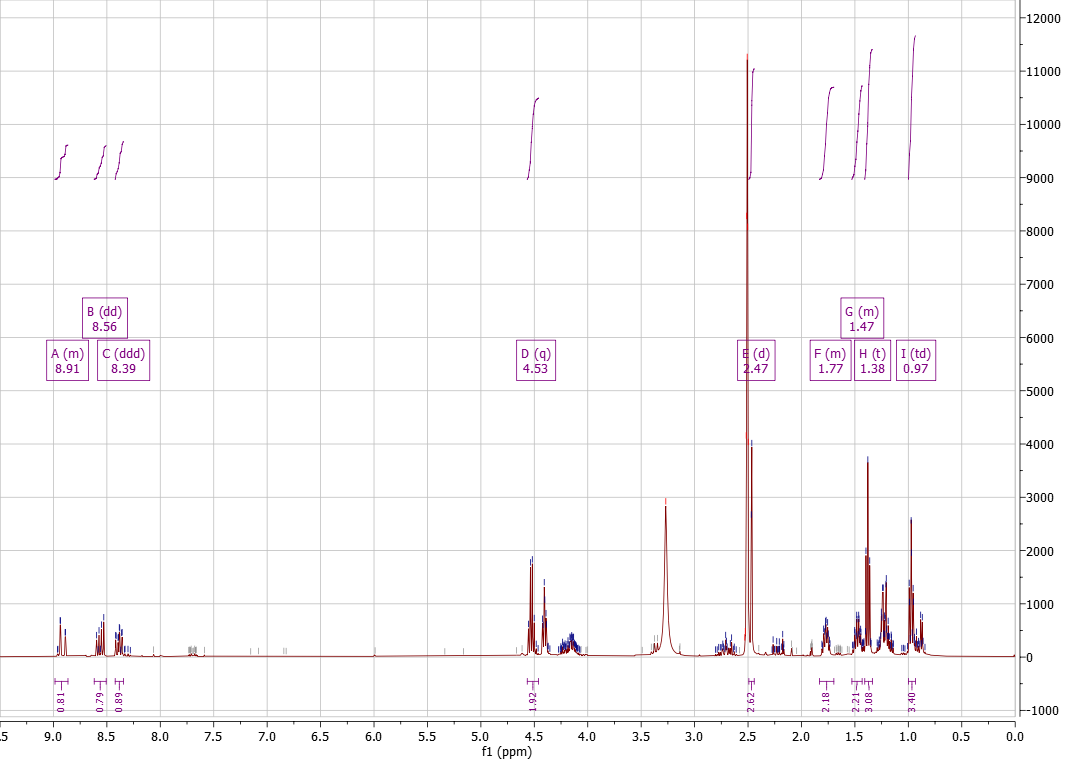


T-140 ^13^C-NMR


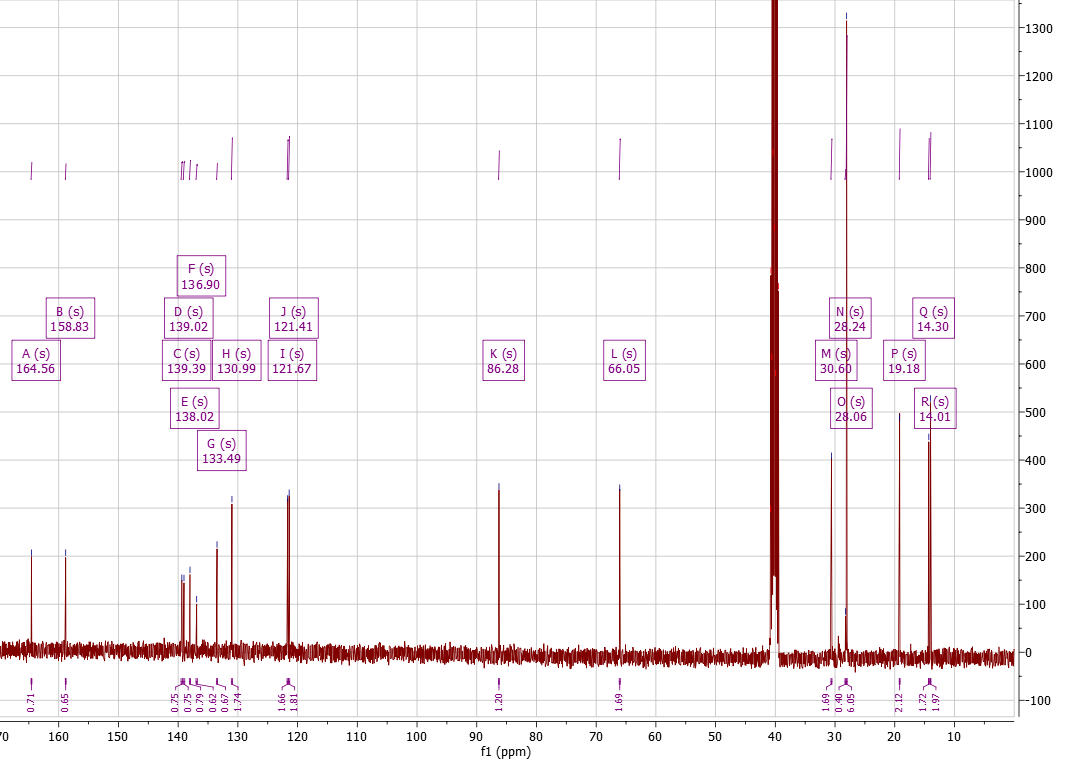


T-140 UPLC-MS


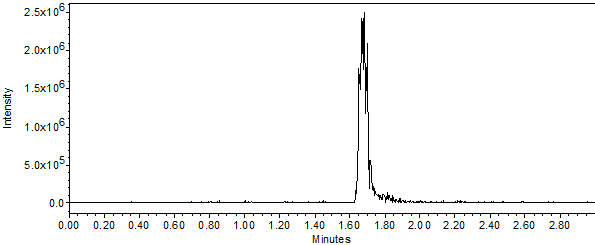


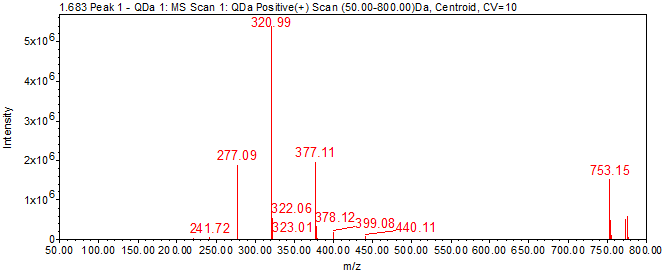


T-141 IR


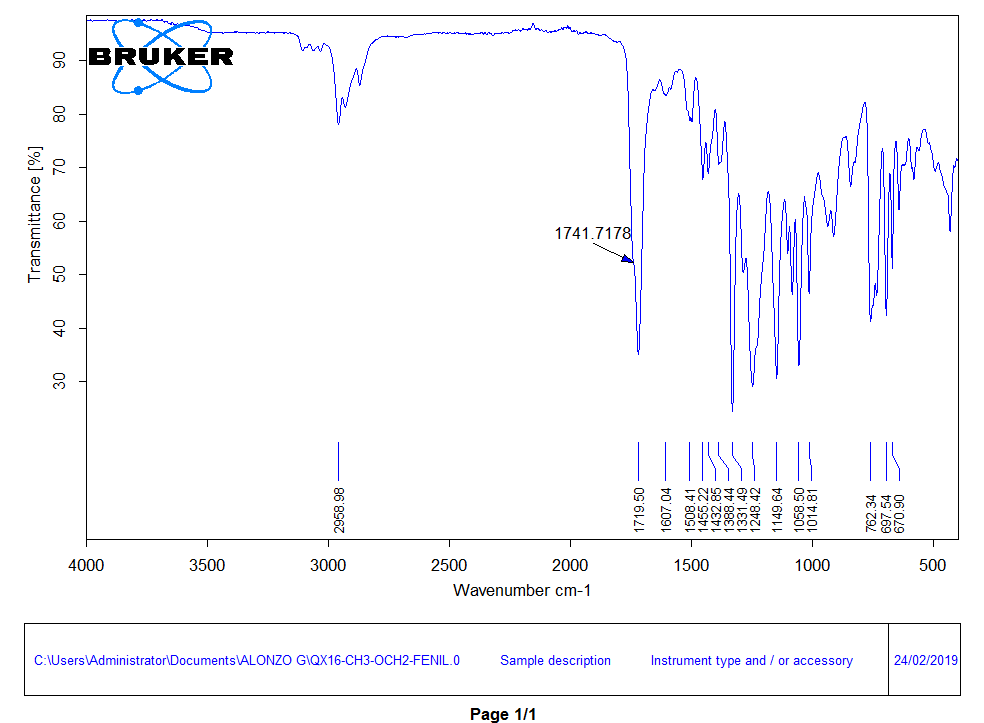


T-141 ^1^H-NMR


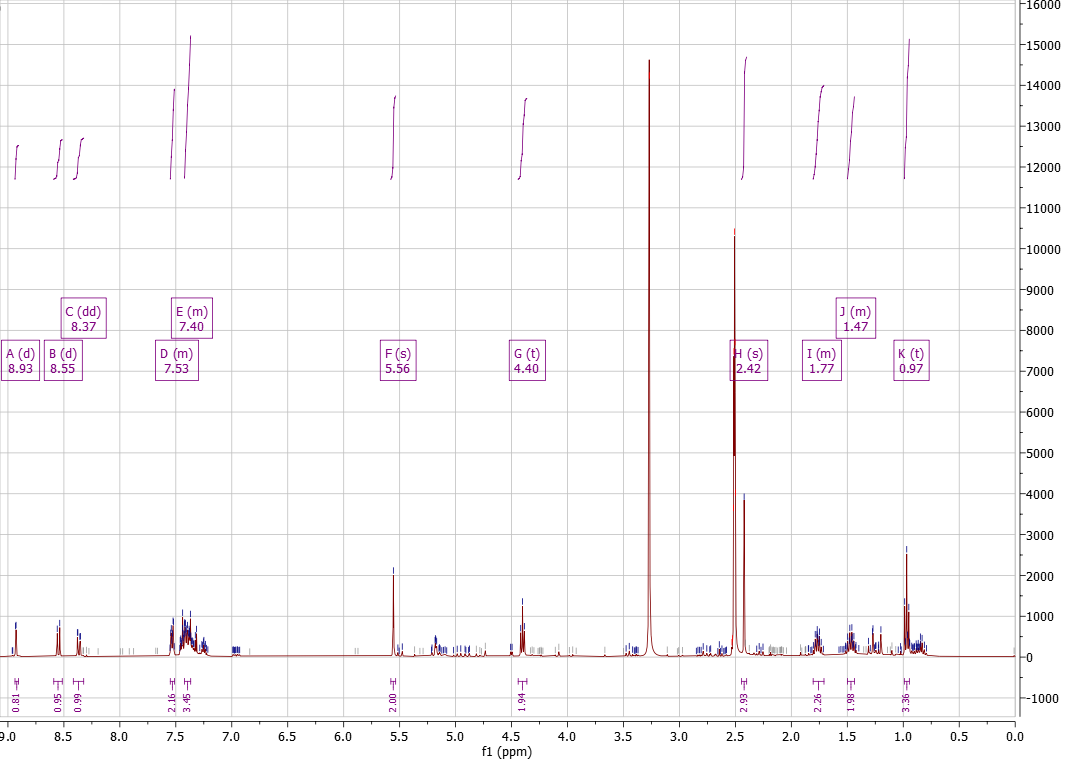


T-141 ^13^C-NMR


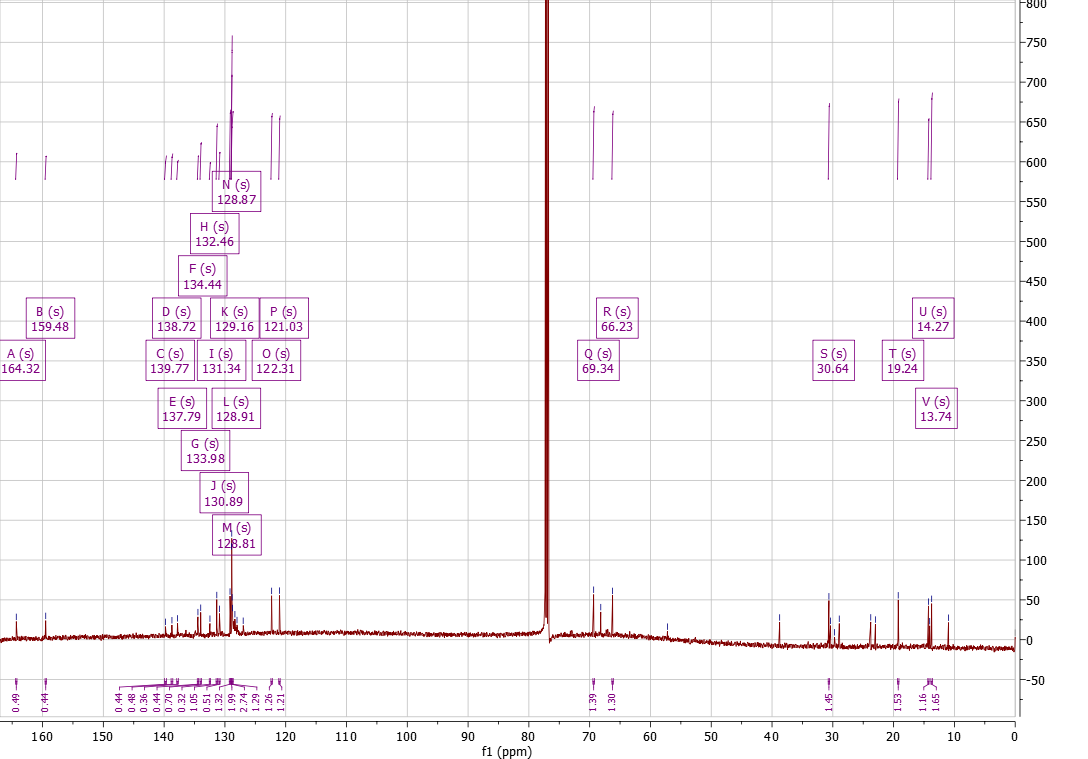


T-141 UPLC-MS


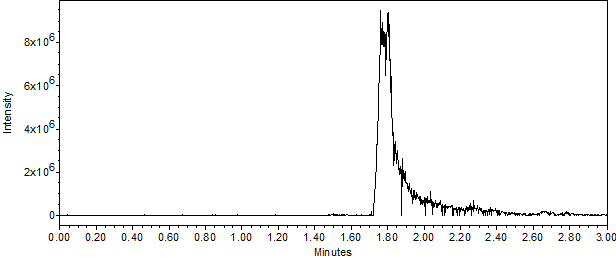


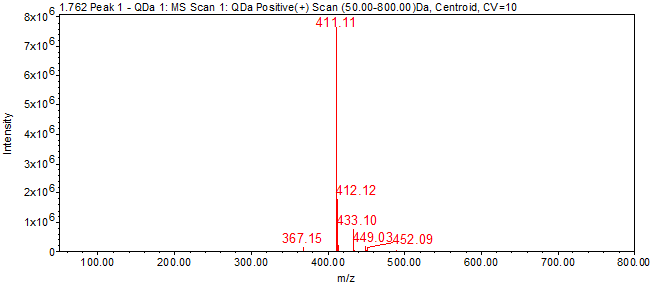


T-142 IR


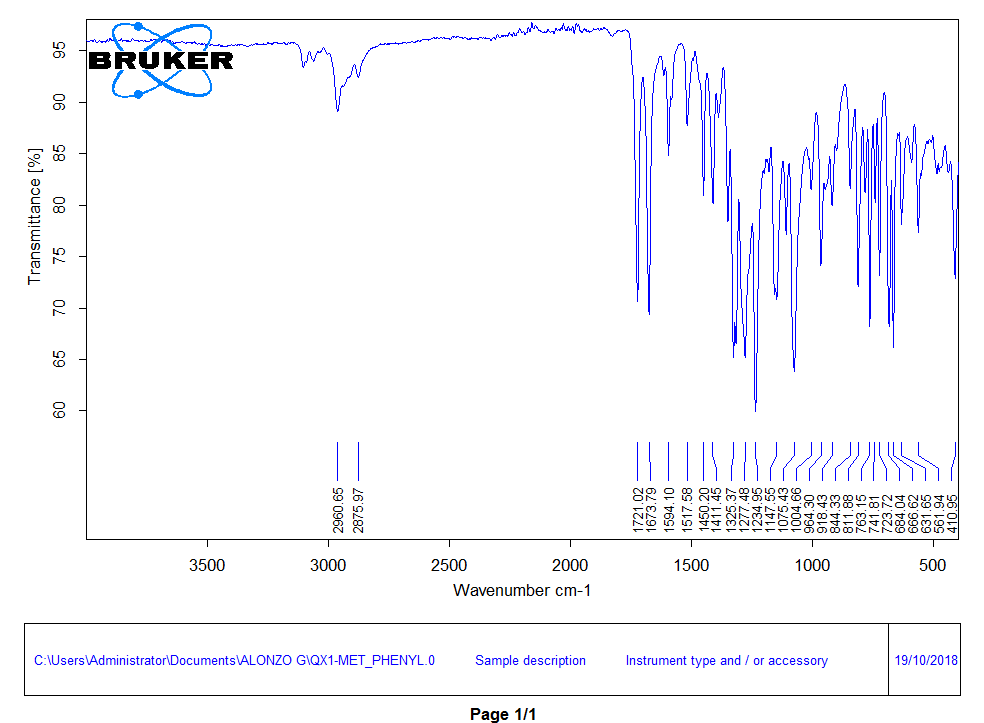


T-142 ^1^H-NMR


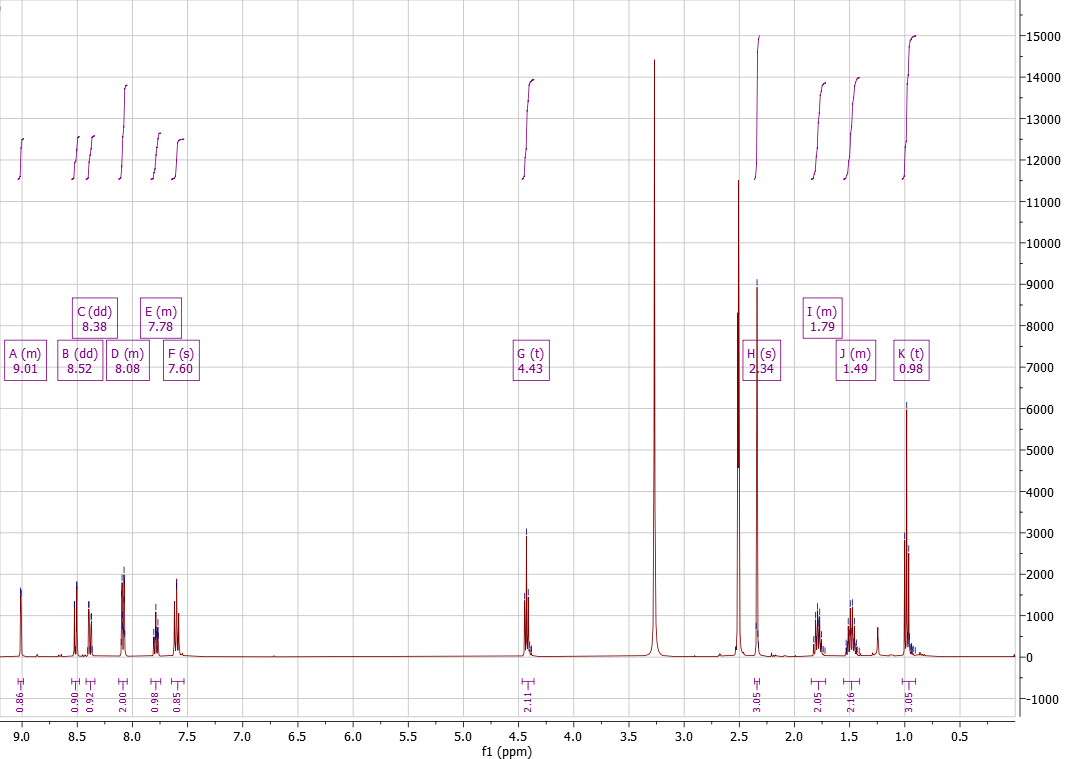


T-142 ^13^C-NMR


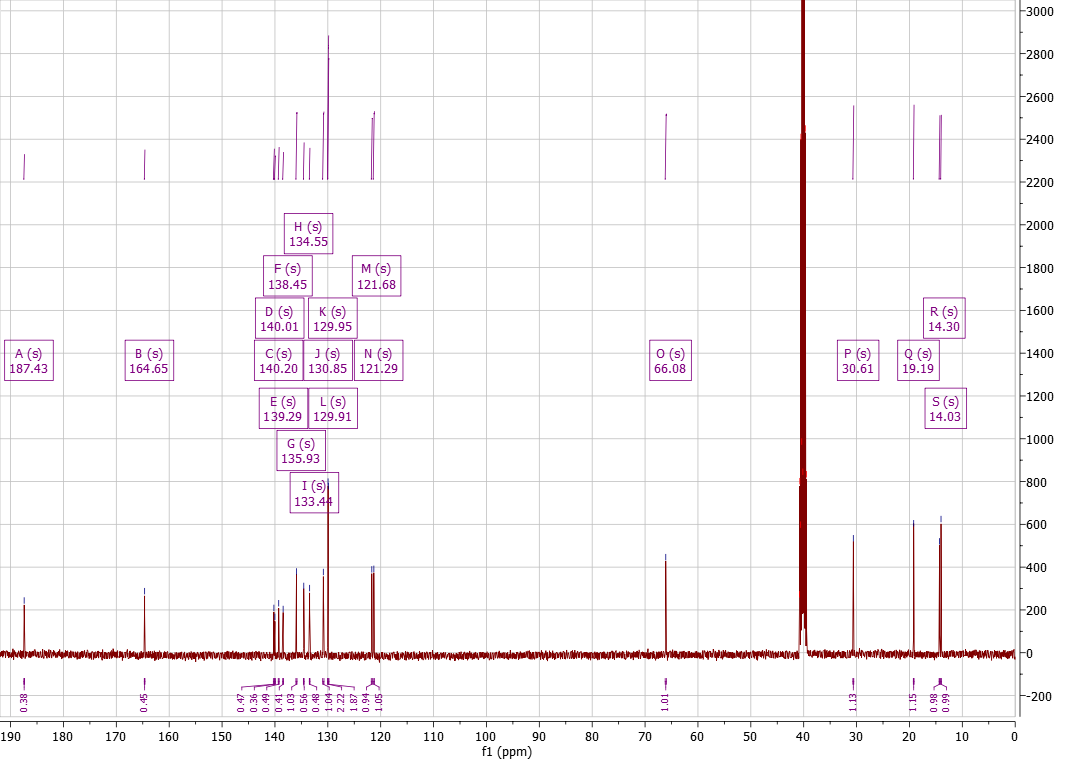


T-142 UPLC-MS


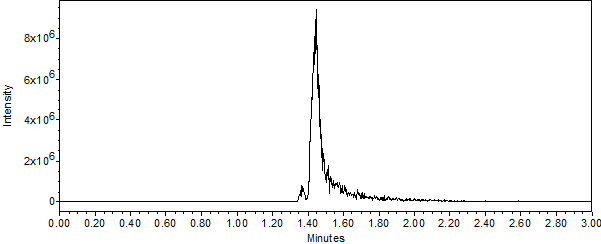


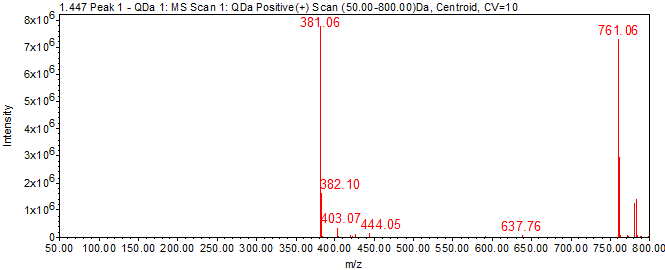


T-143 IR


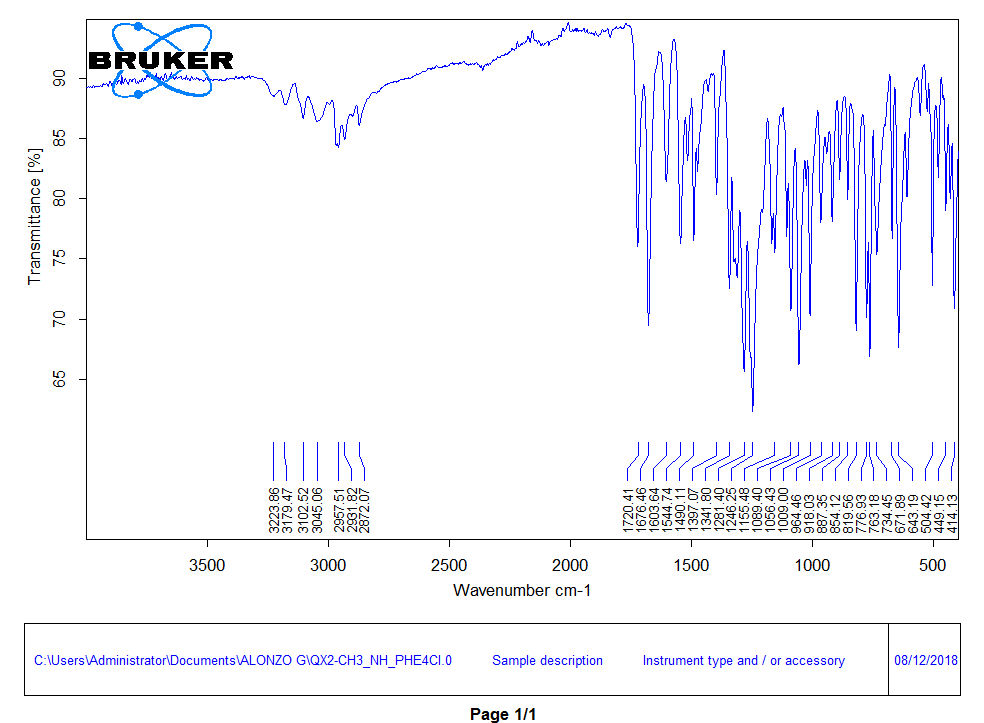


T-143 ^1^H-NMR


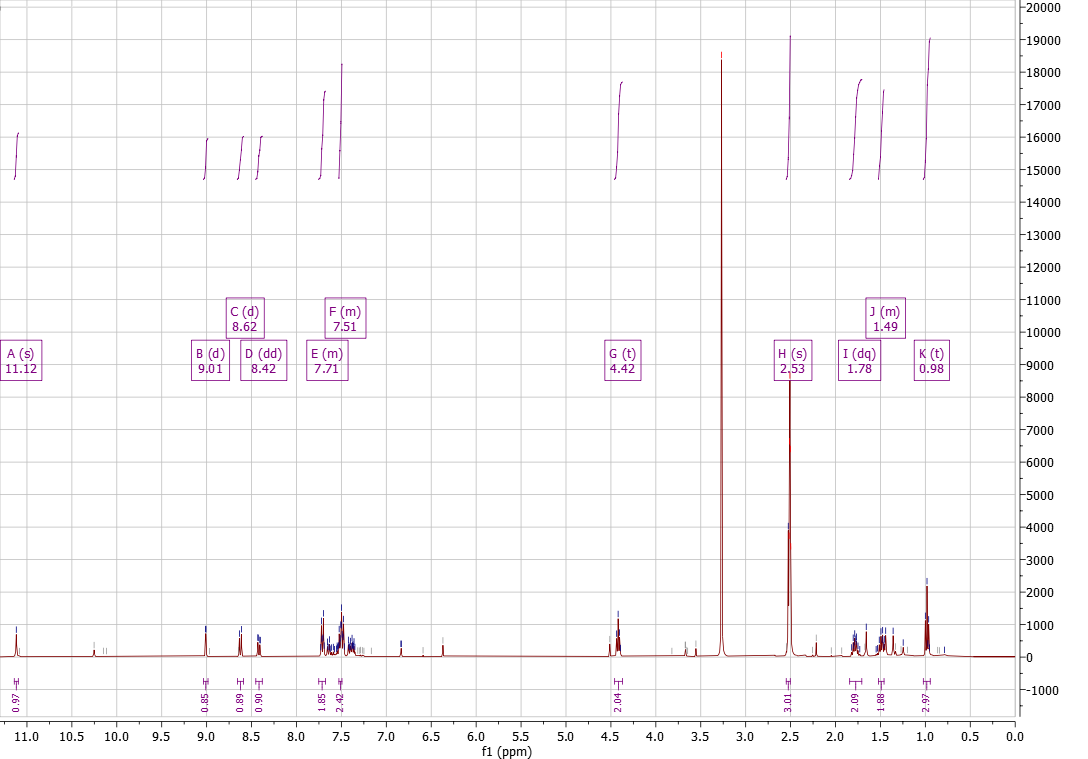


T-143 ^13^C-NMR


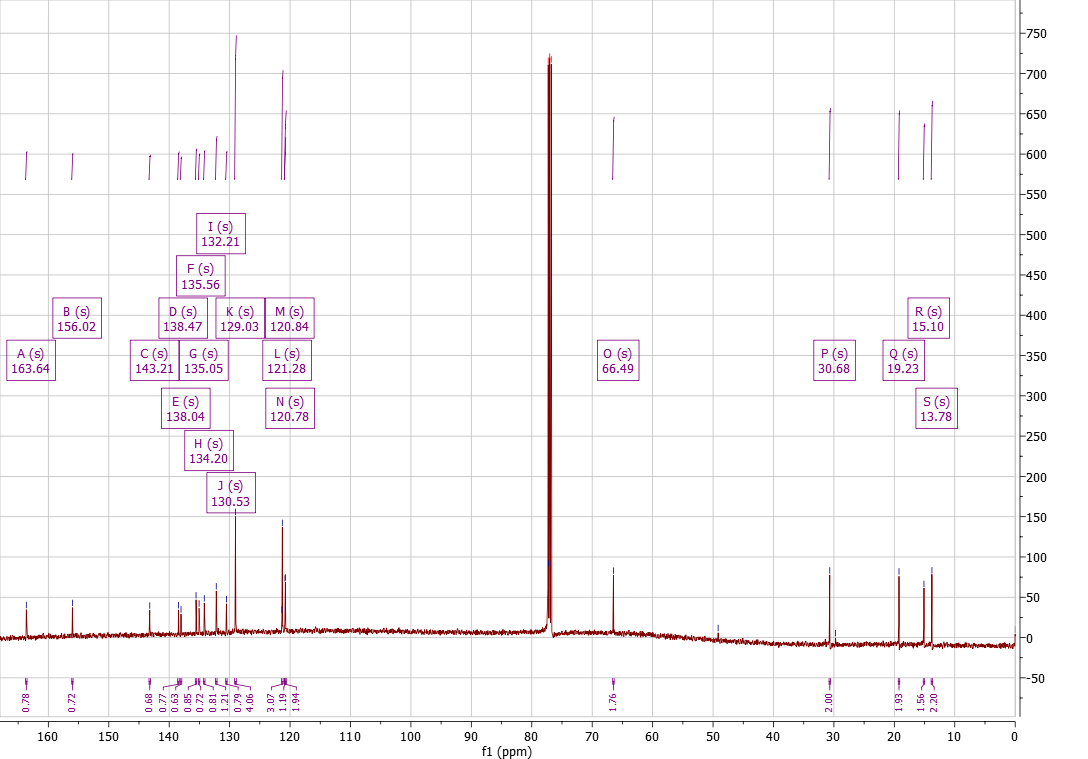


T-143 UPLC-MS


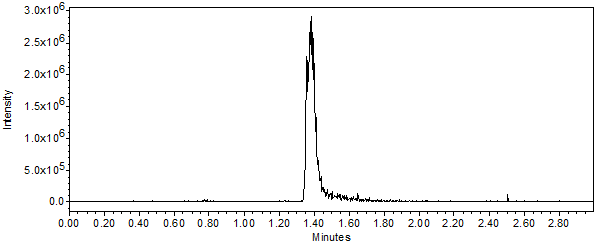


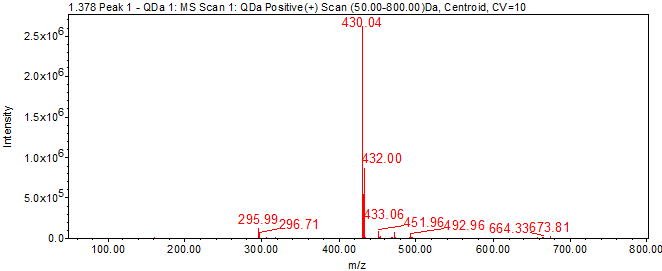


T-144 IR


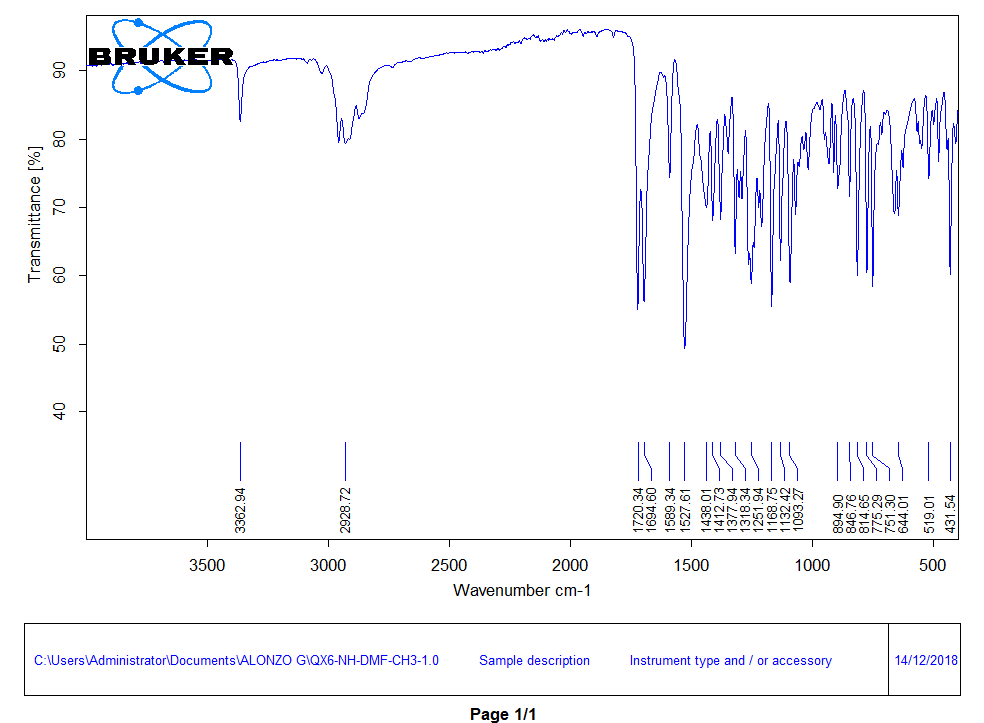


T-144 ^1^H-NMR


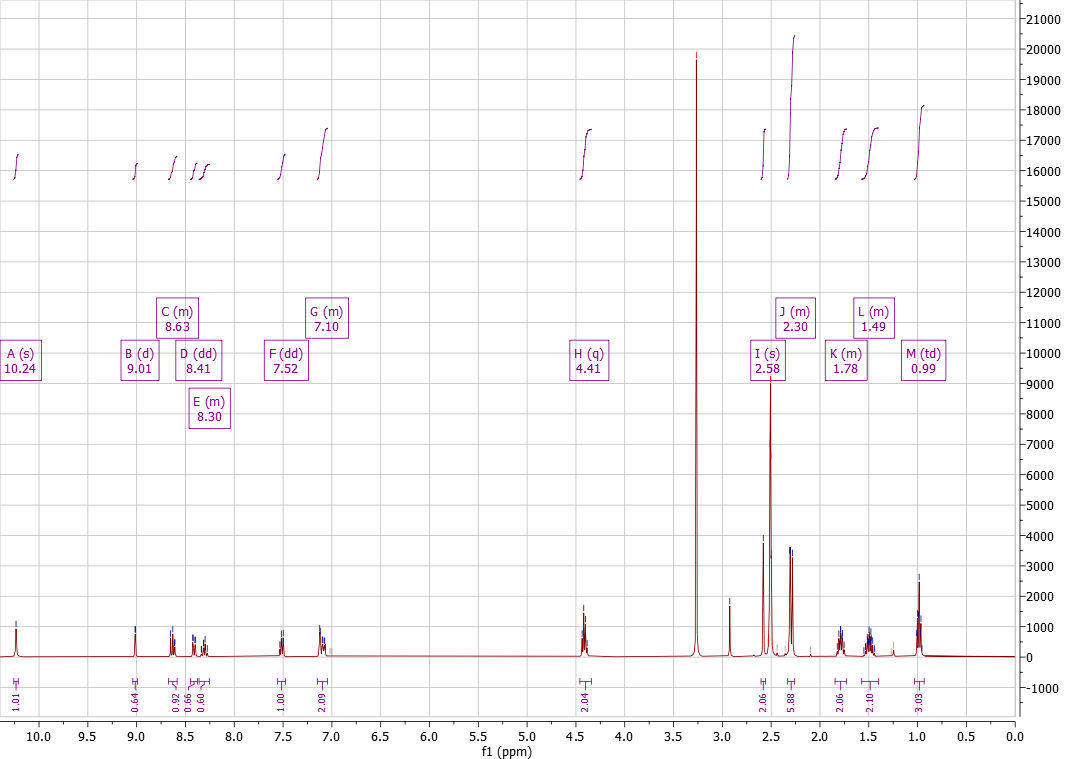


T-144 ^13^C-NMR


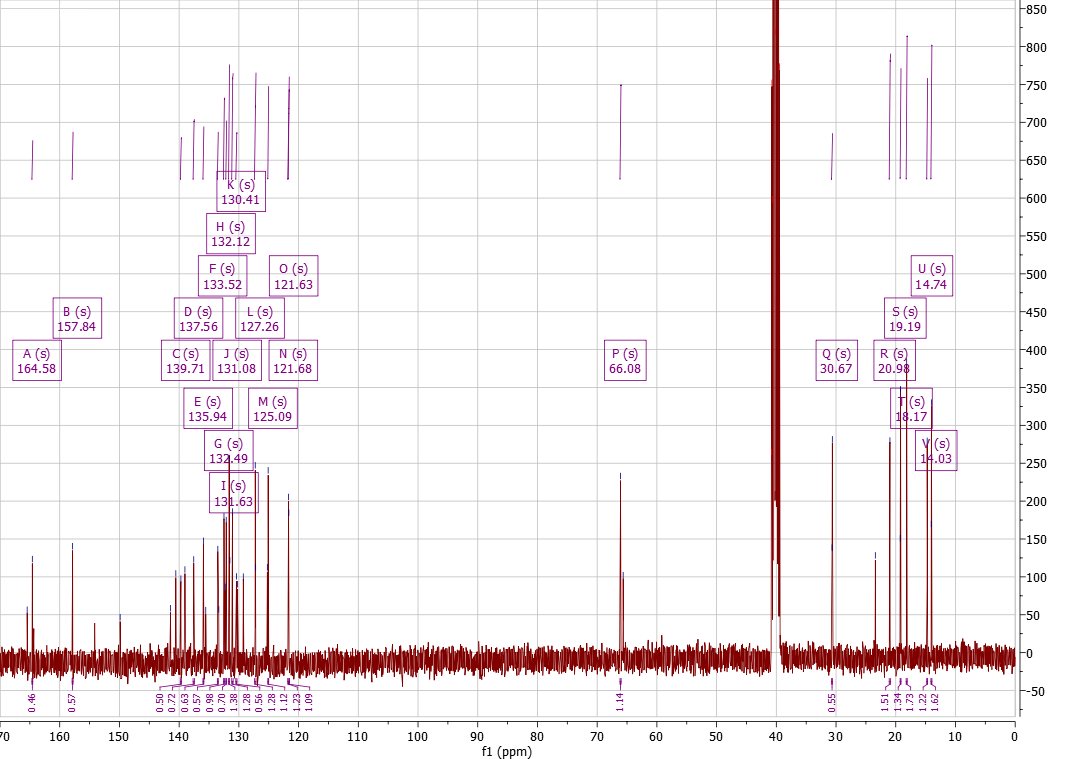


T-144 UPLC-MS


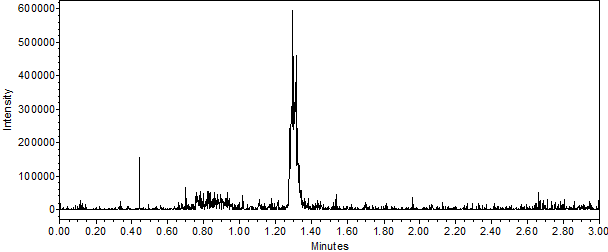


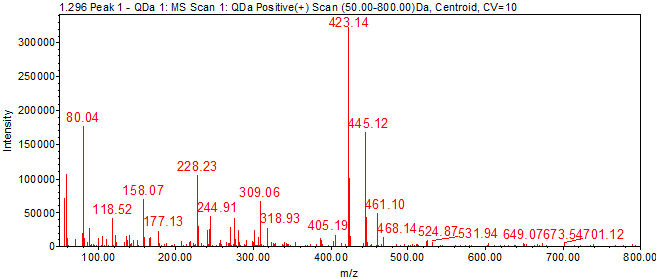


T-145 IR


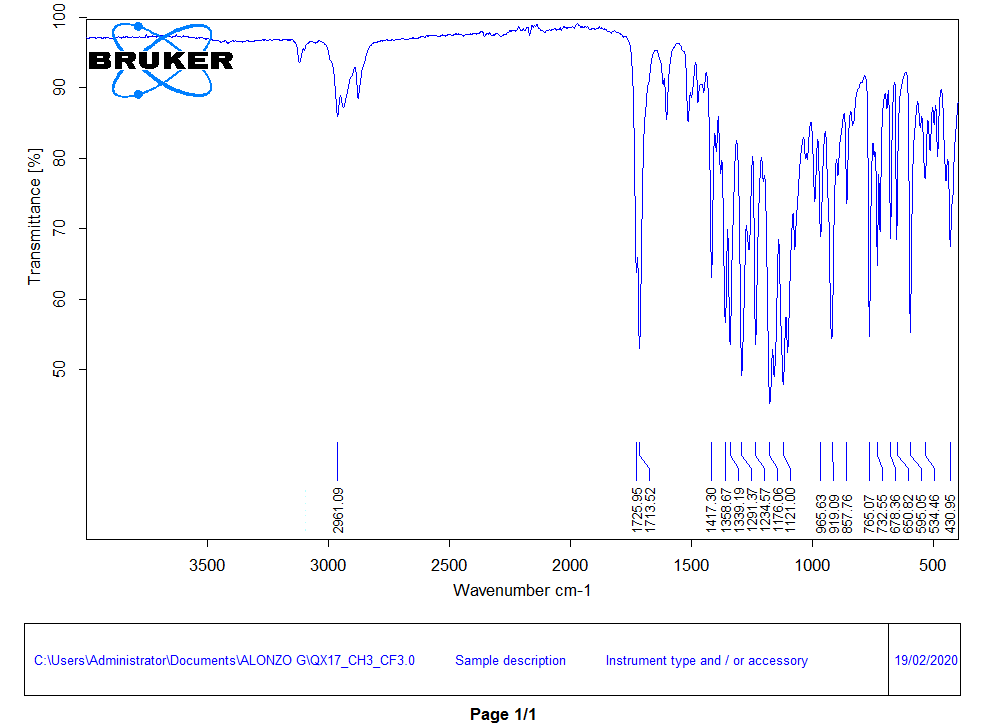


T-145 ^1^H-NMR


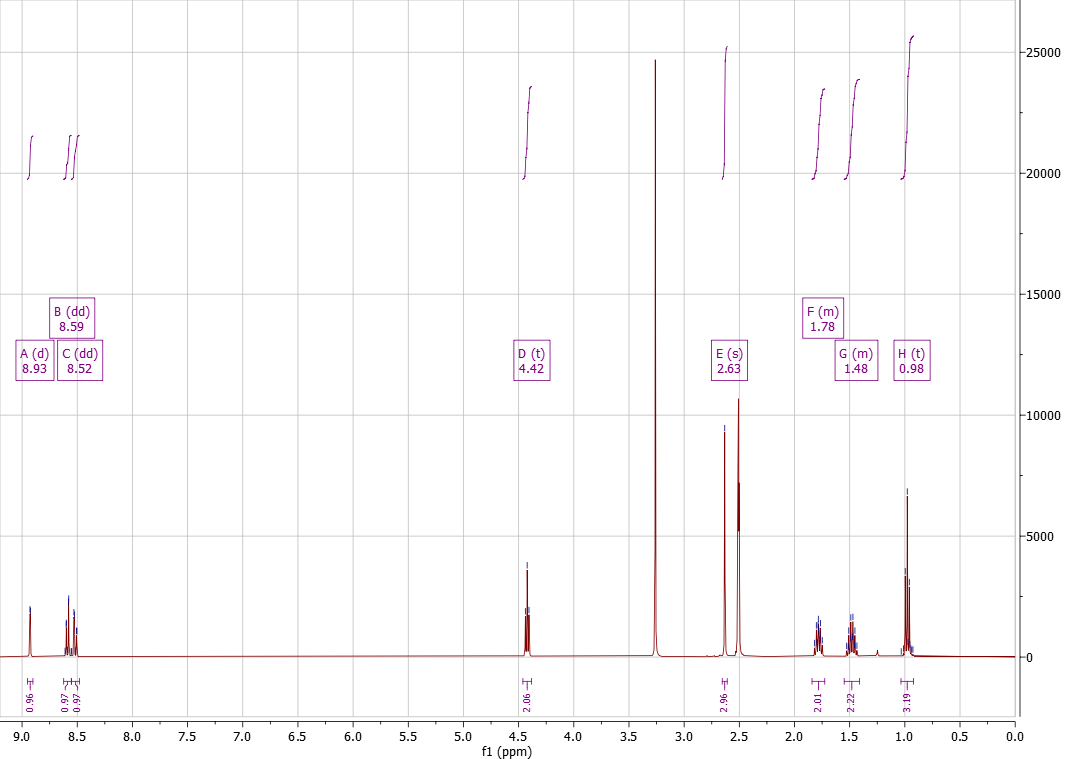


T-145 ^13^C-NMR


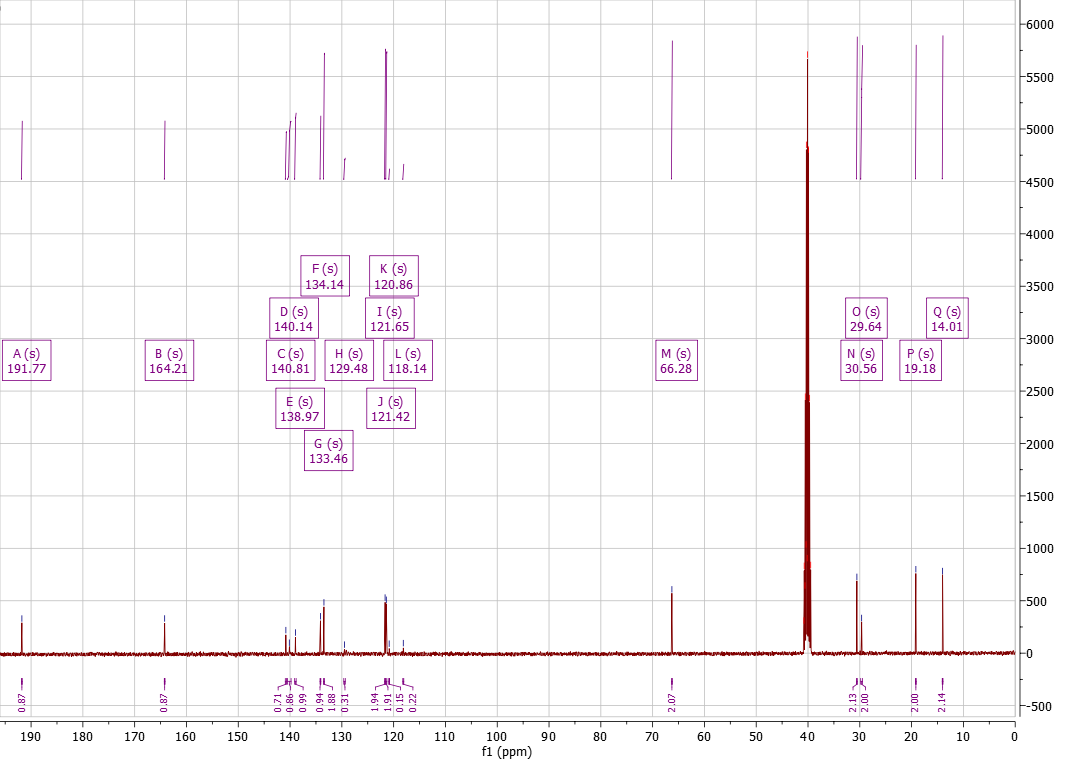


T-145 UPLC-MS


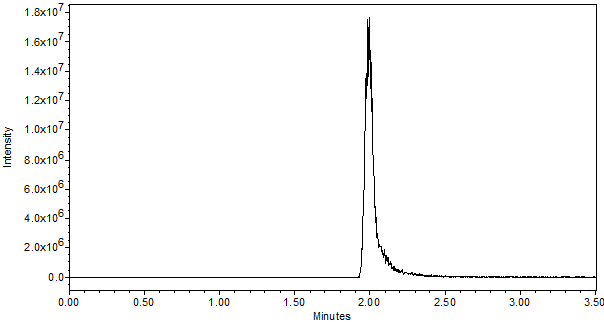


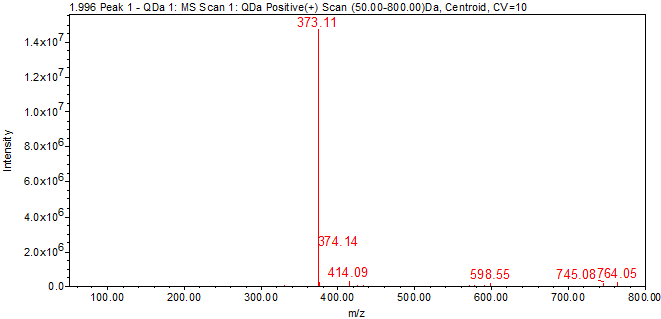


T-146 IR


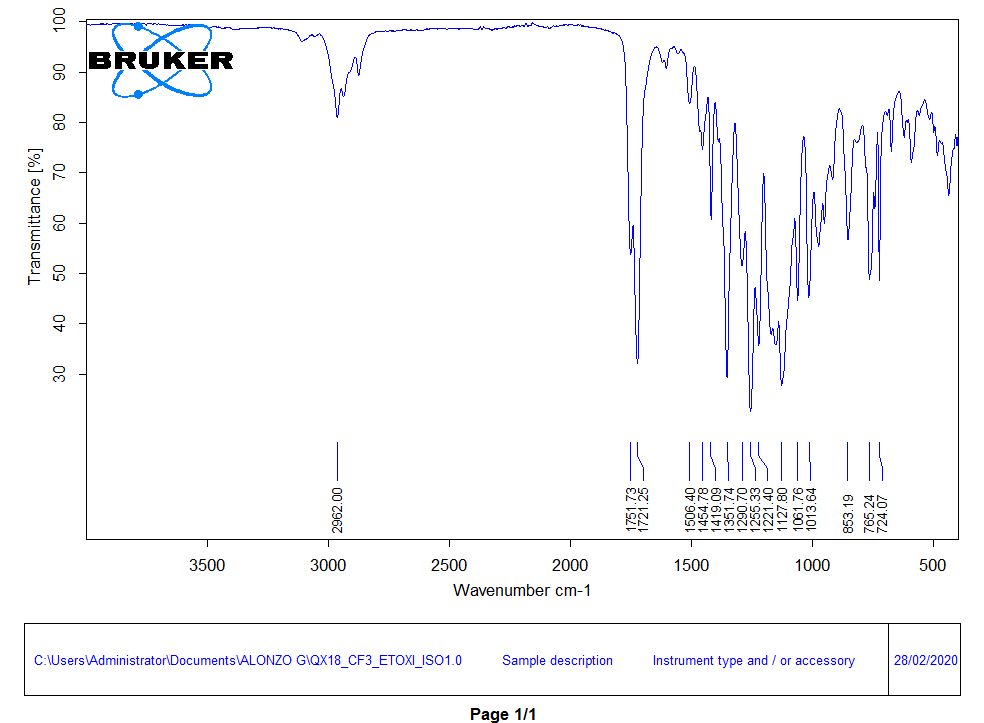


T-146 ^1^H-NMR


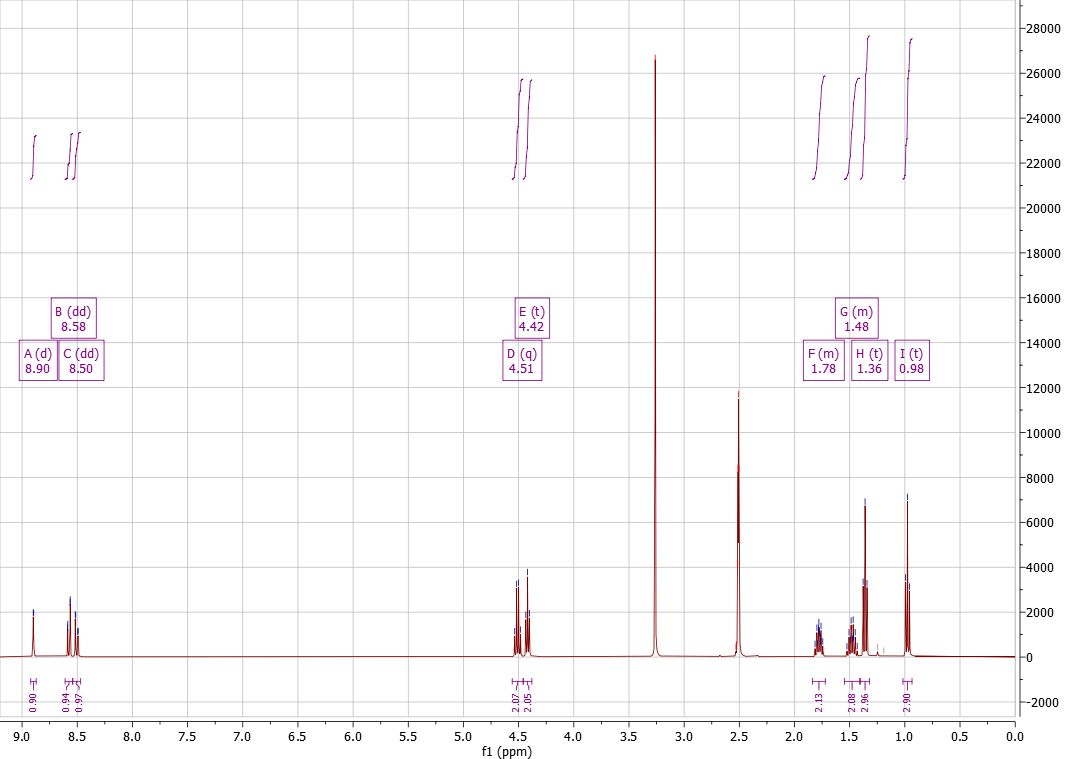


T-146 ^13^C-NMR


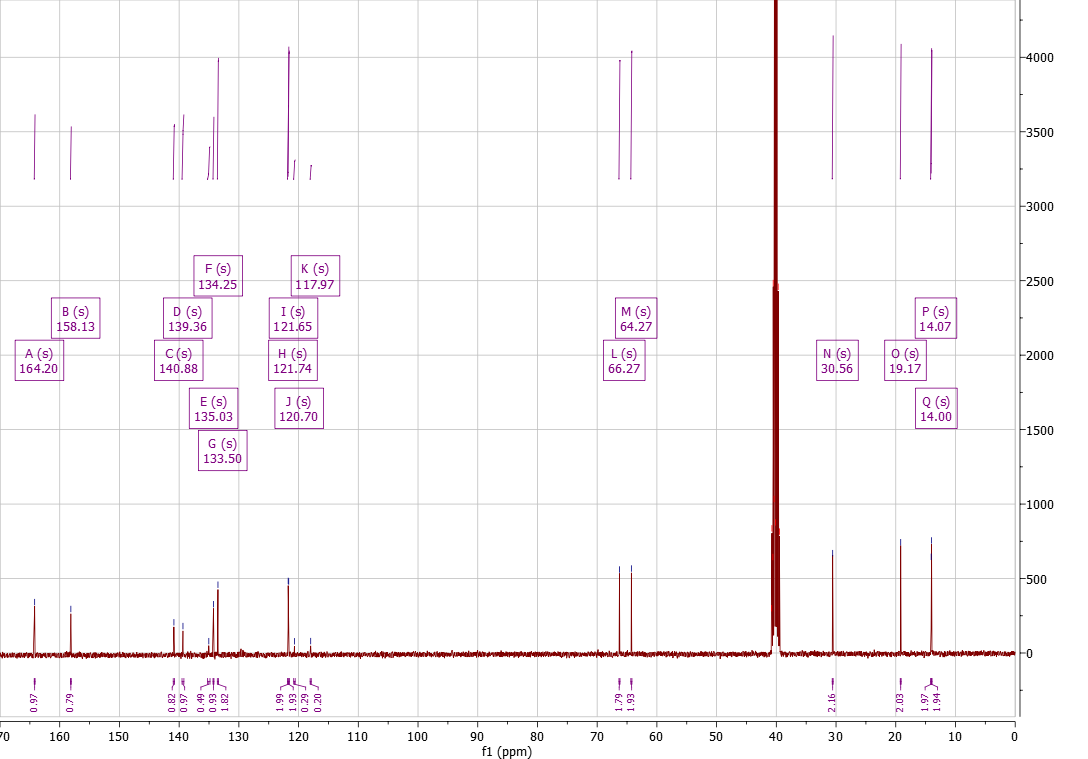


T-146 UPLC-MS


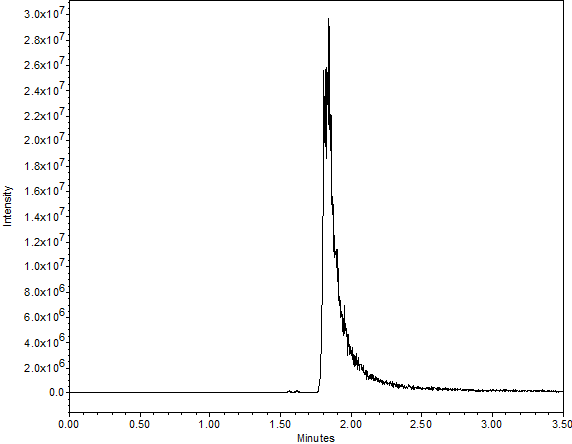


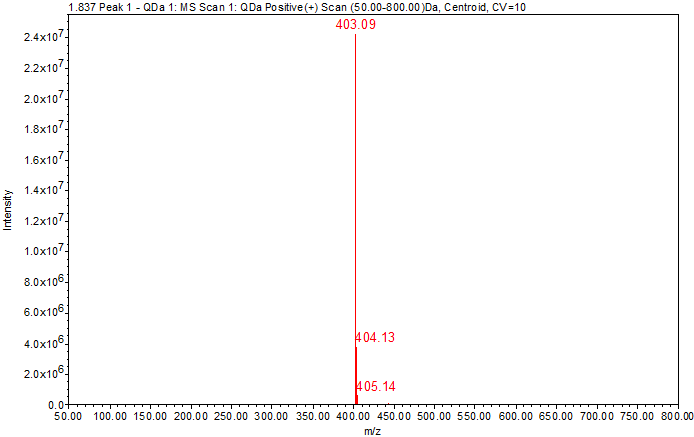


T-148 IR


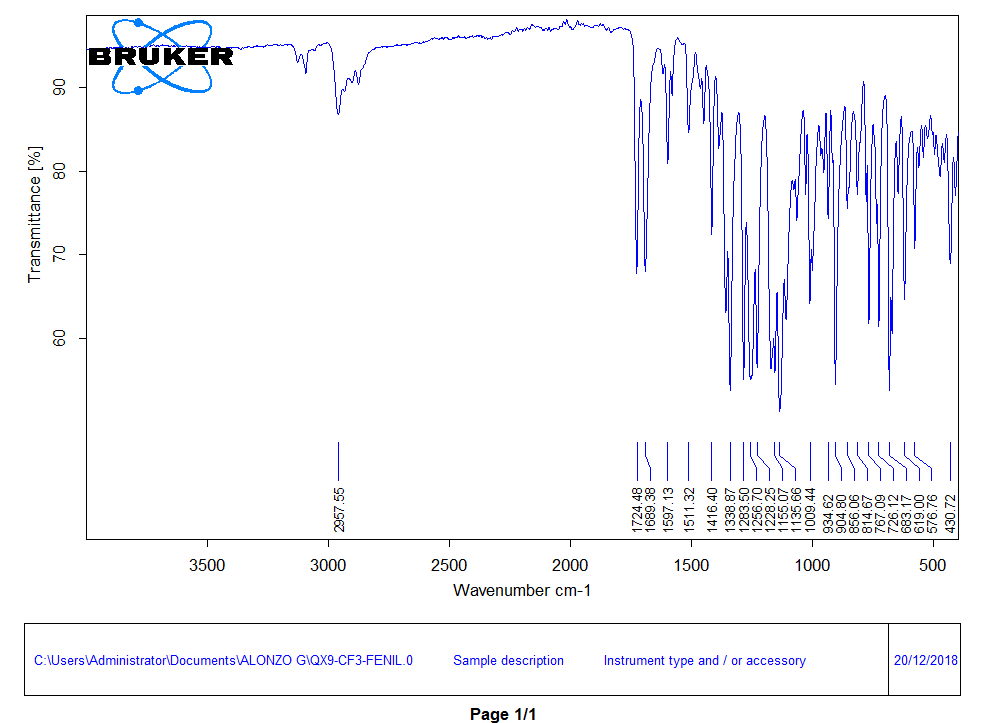


T-148 ^1^H-NMR


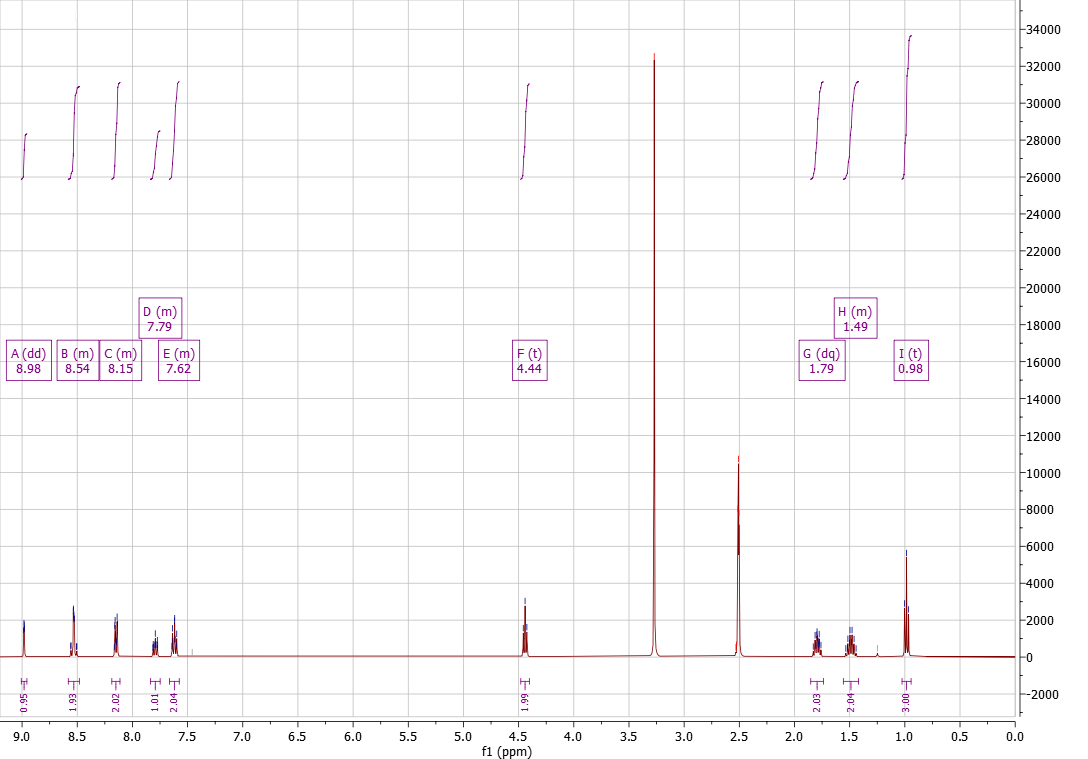


T-148 ^13^C-NMR


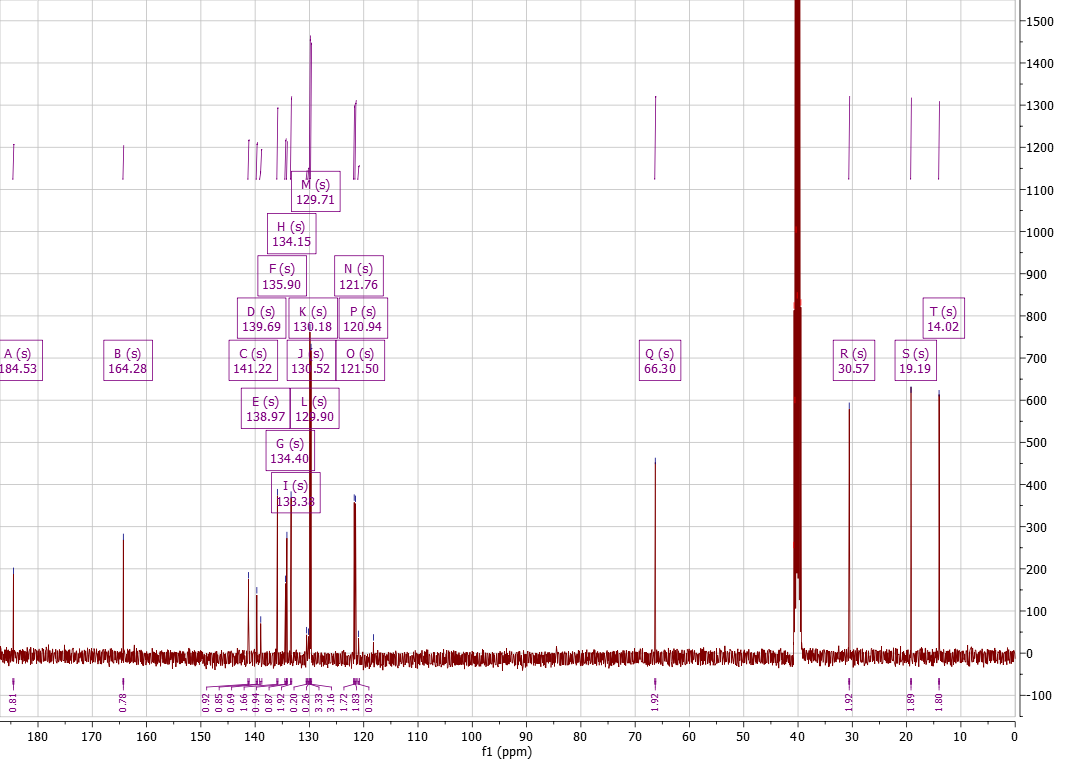


T-148 UPLC-MS


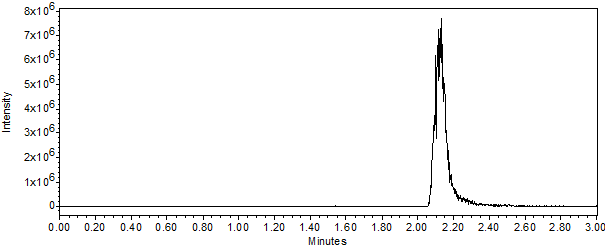


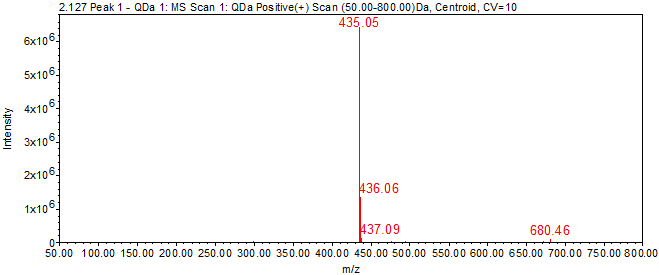


T-149 IR


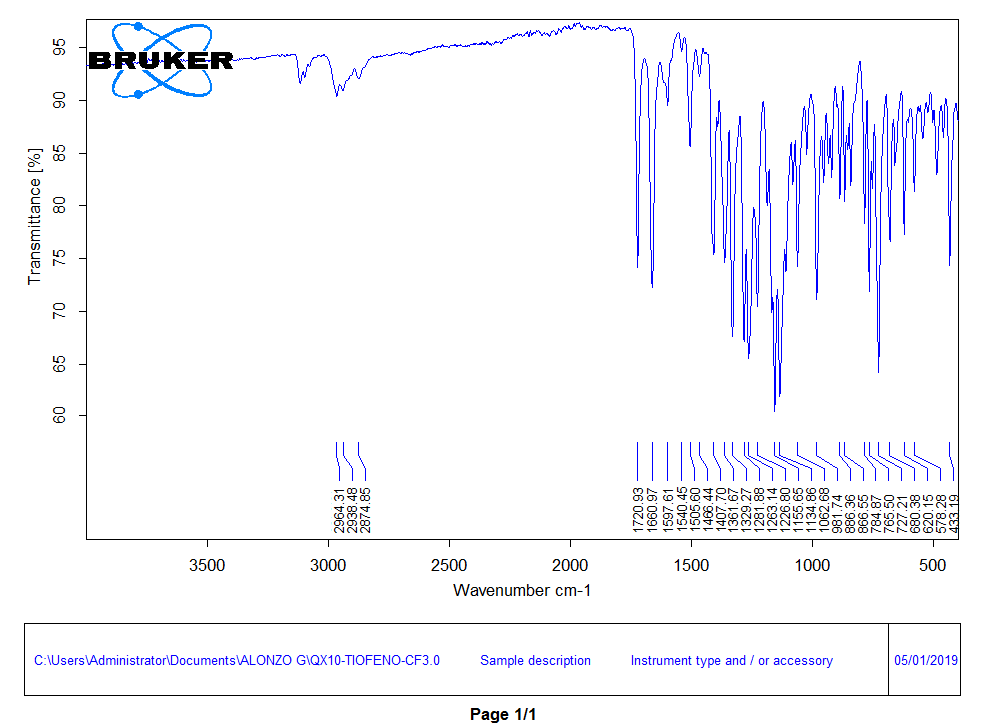


T-149 ^1^H-NMR


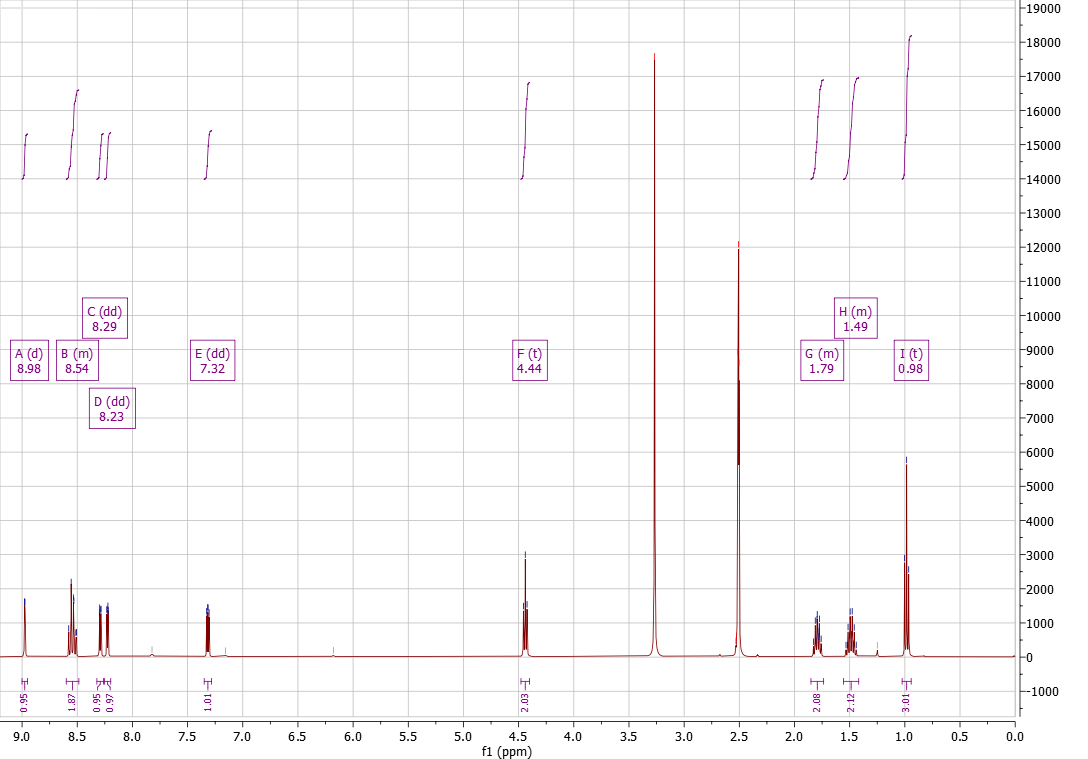


T-149 ^13^C-NMR


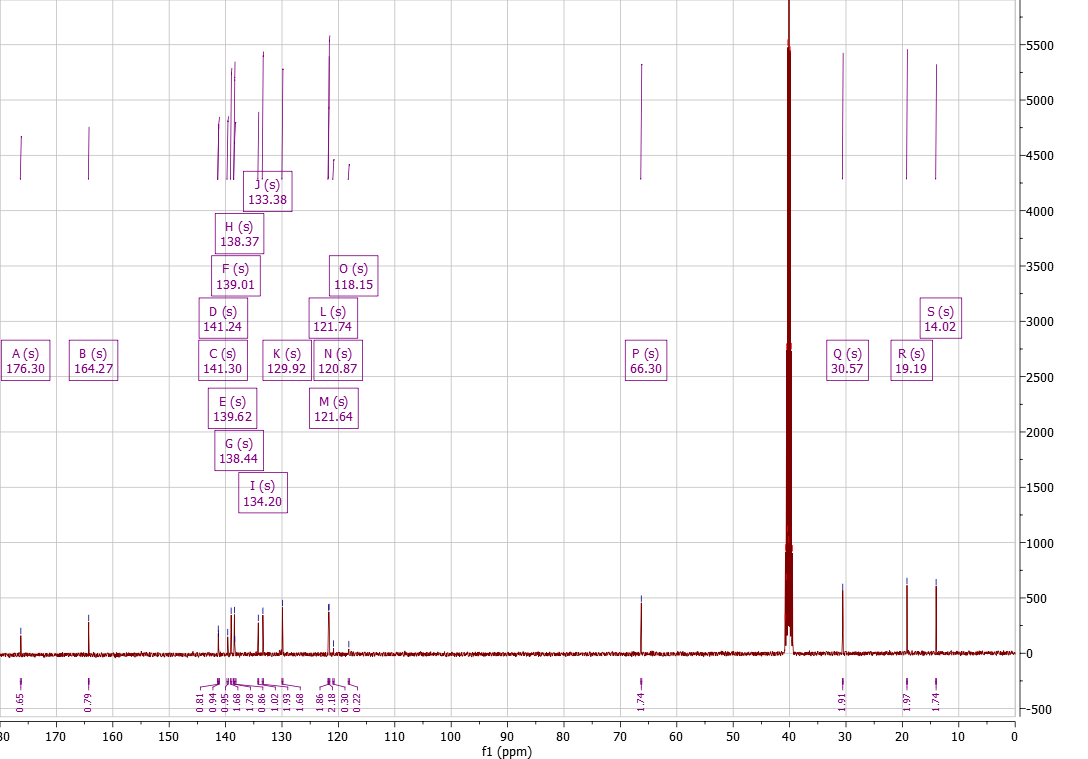


T-149 UPLC-MS


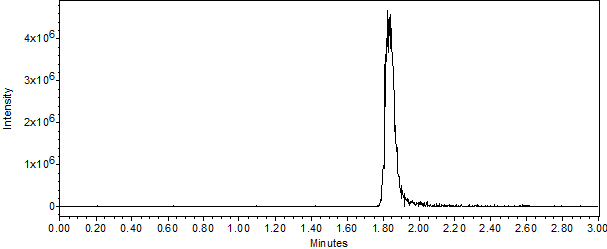


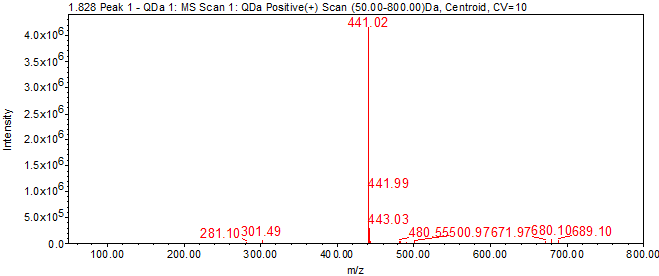


T-150 IR


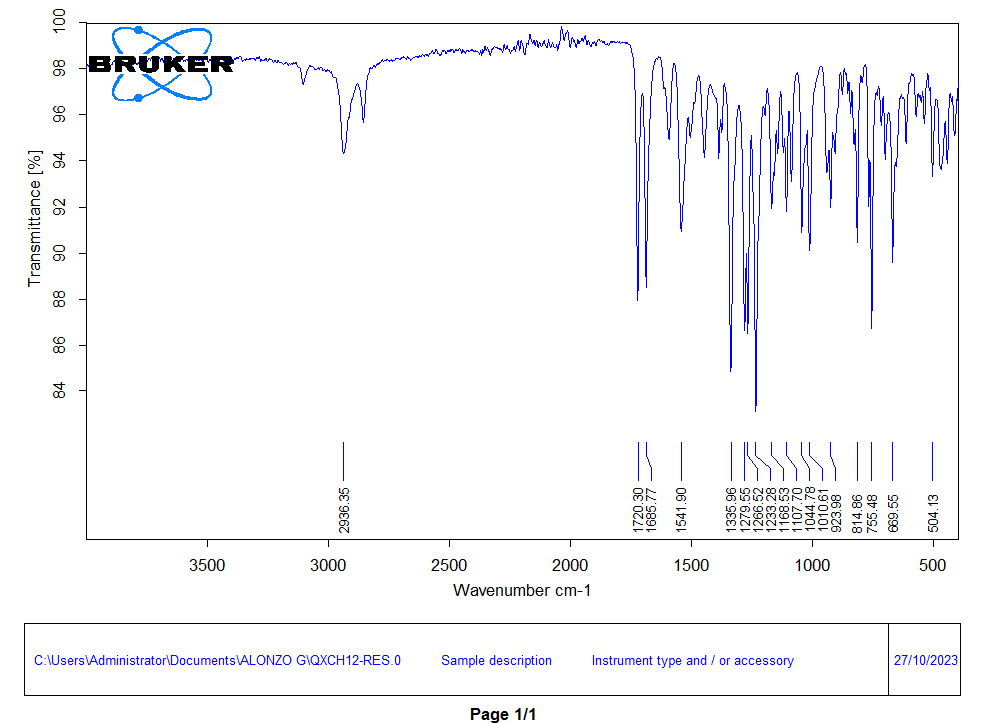


T-150 ^1^H-NMR


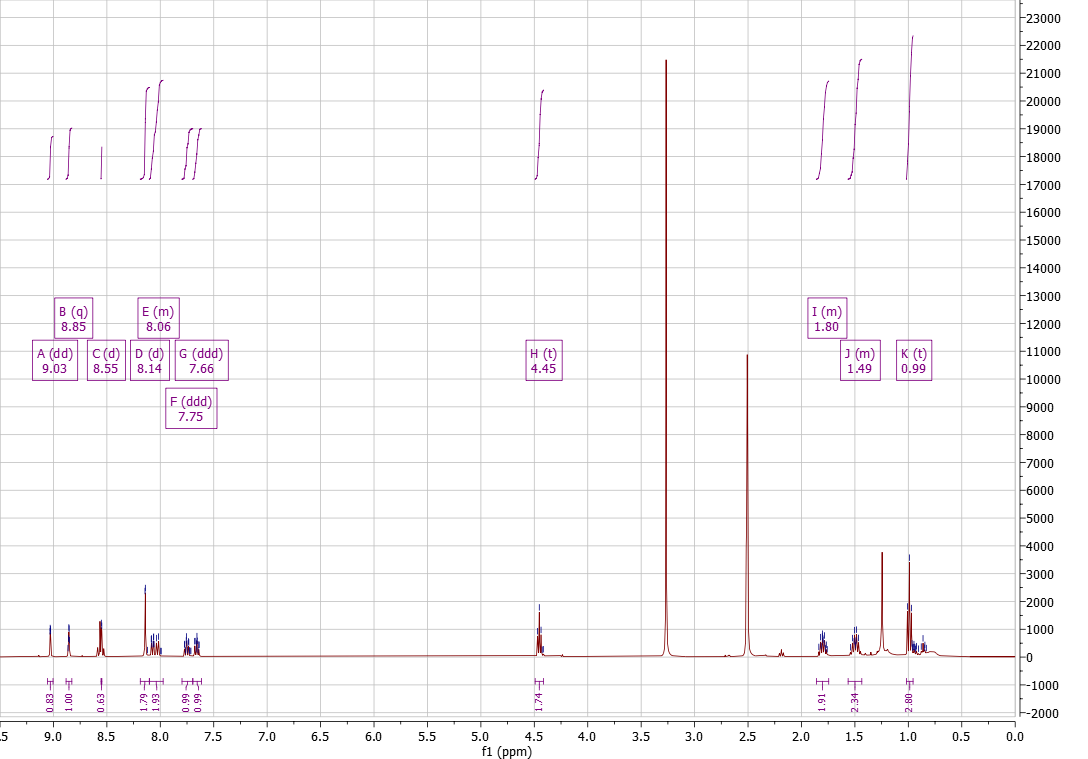


T-150 ^13^C-NMR


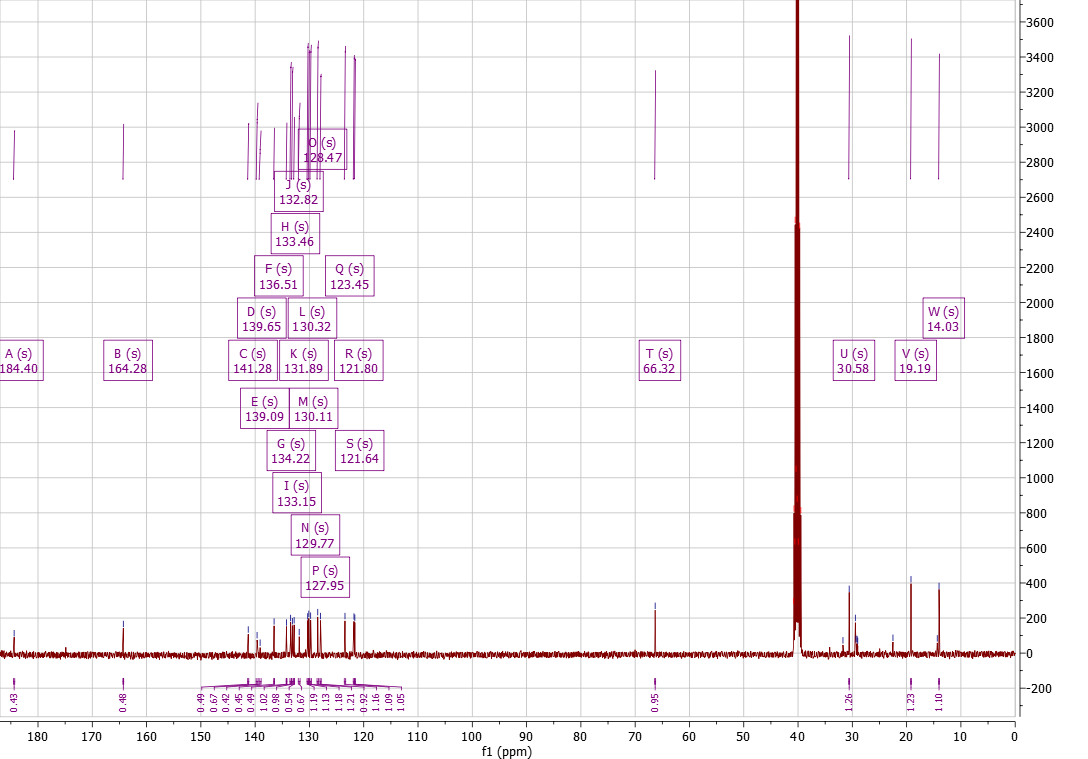


T-150 UPLC-MS


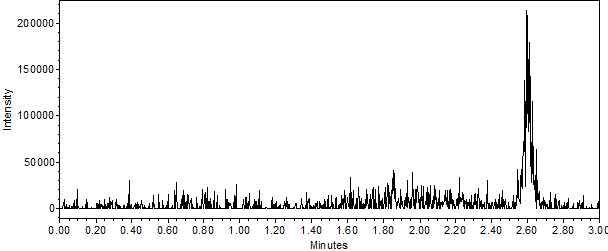


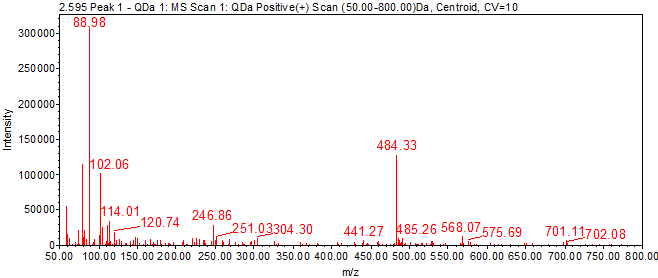


T-151 IR


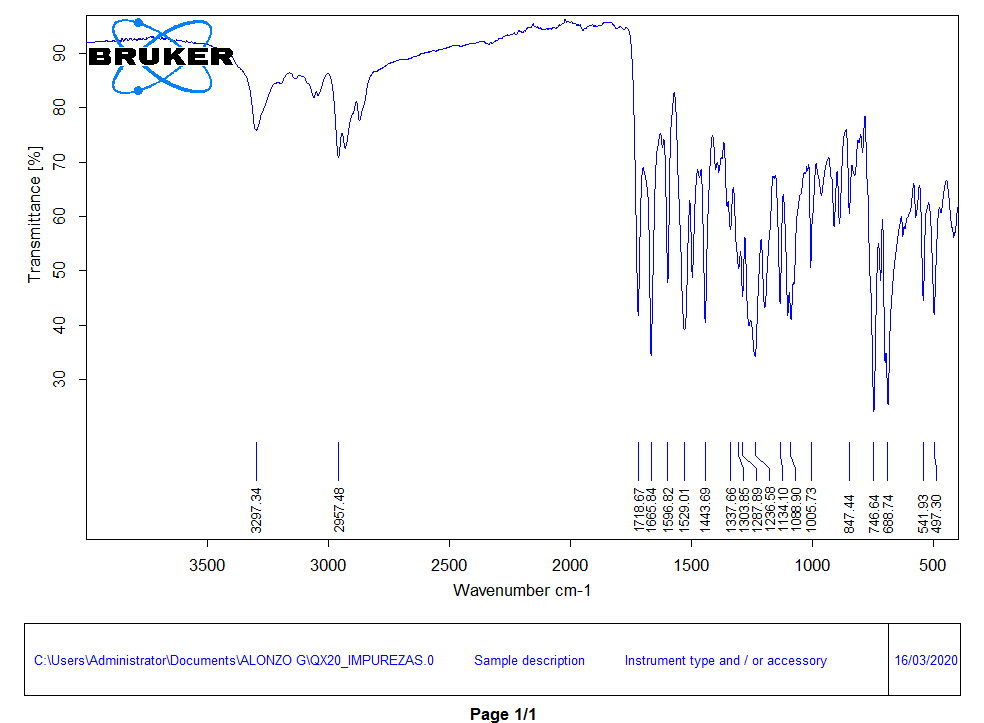


T-151 ^1^H-NMR


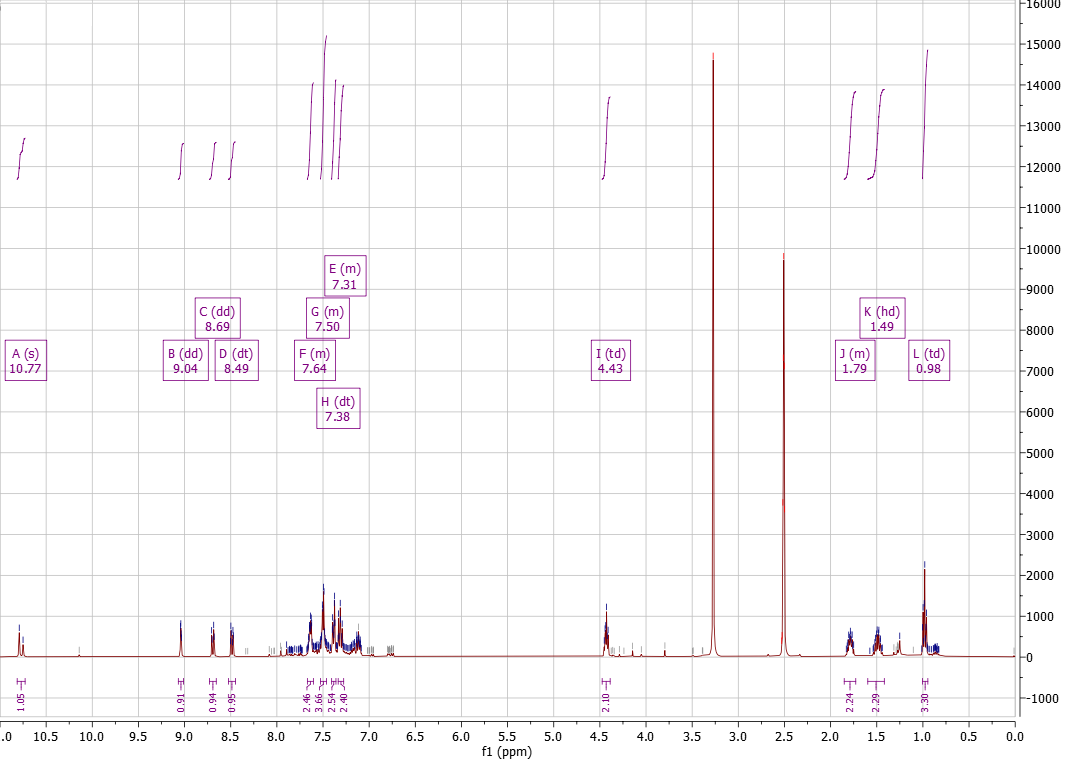


T-151 ^13^C-NMR


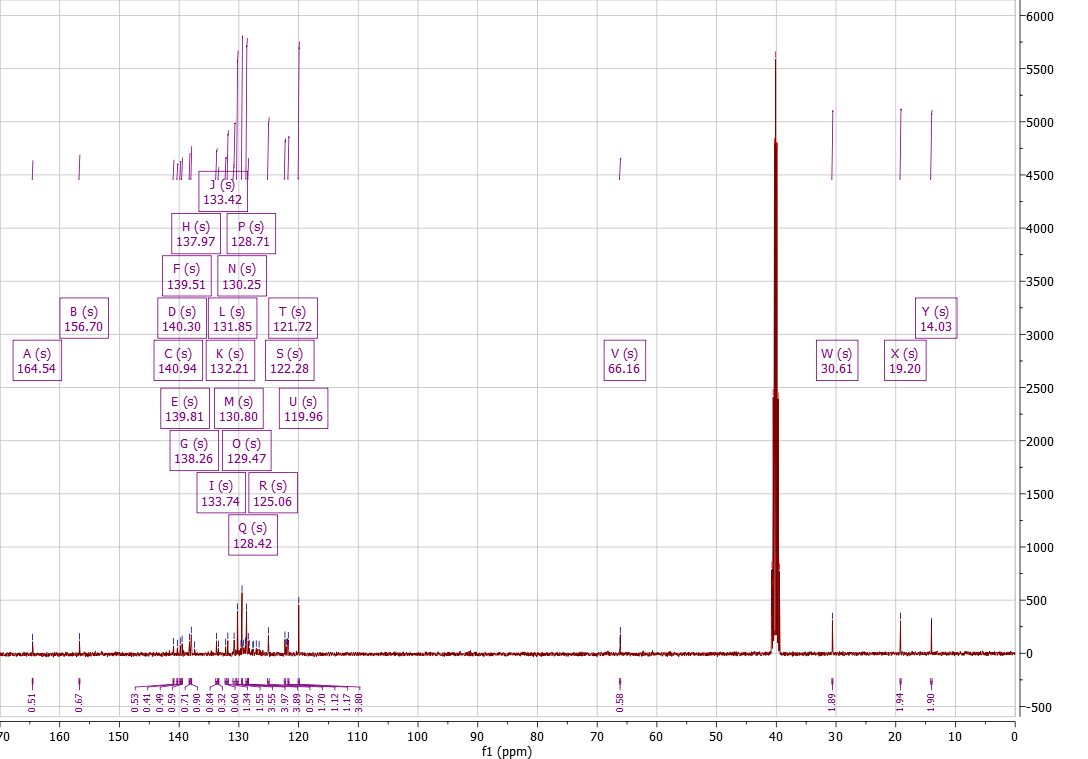


T-151 UPLC-MS


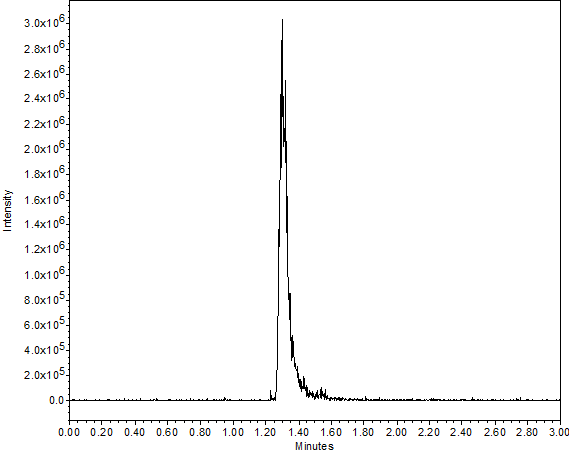


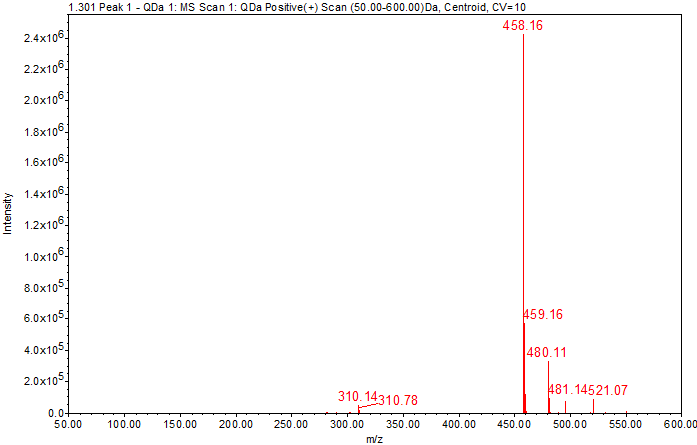


T-155 IR


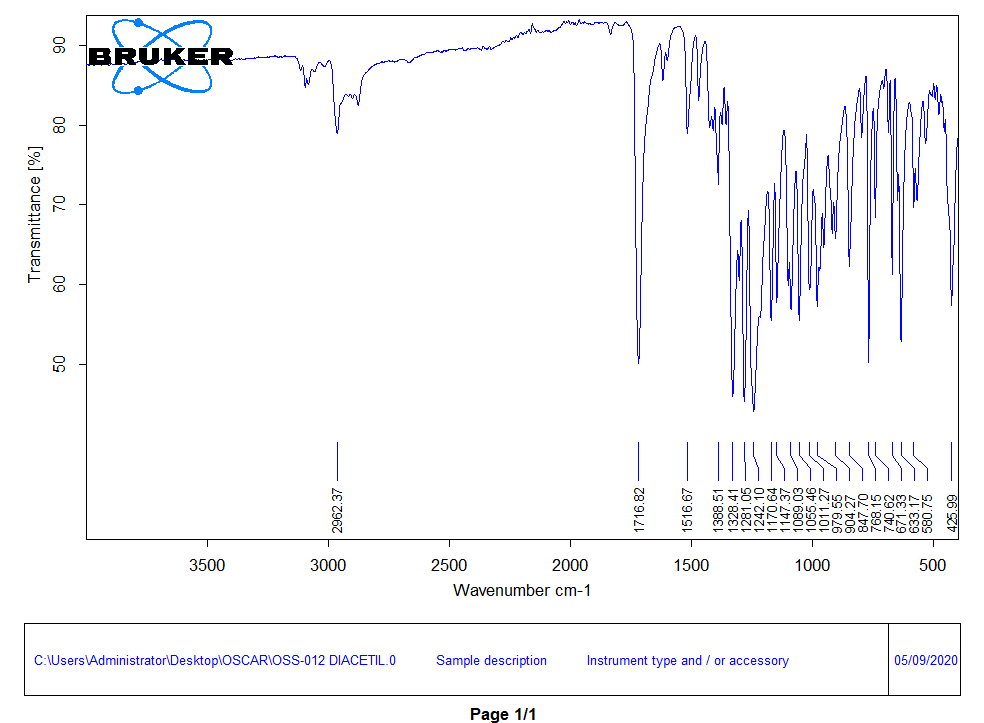


T-155 ^1^H-NMR


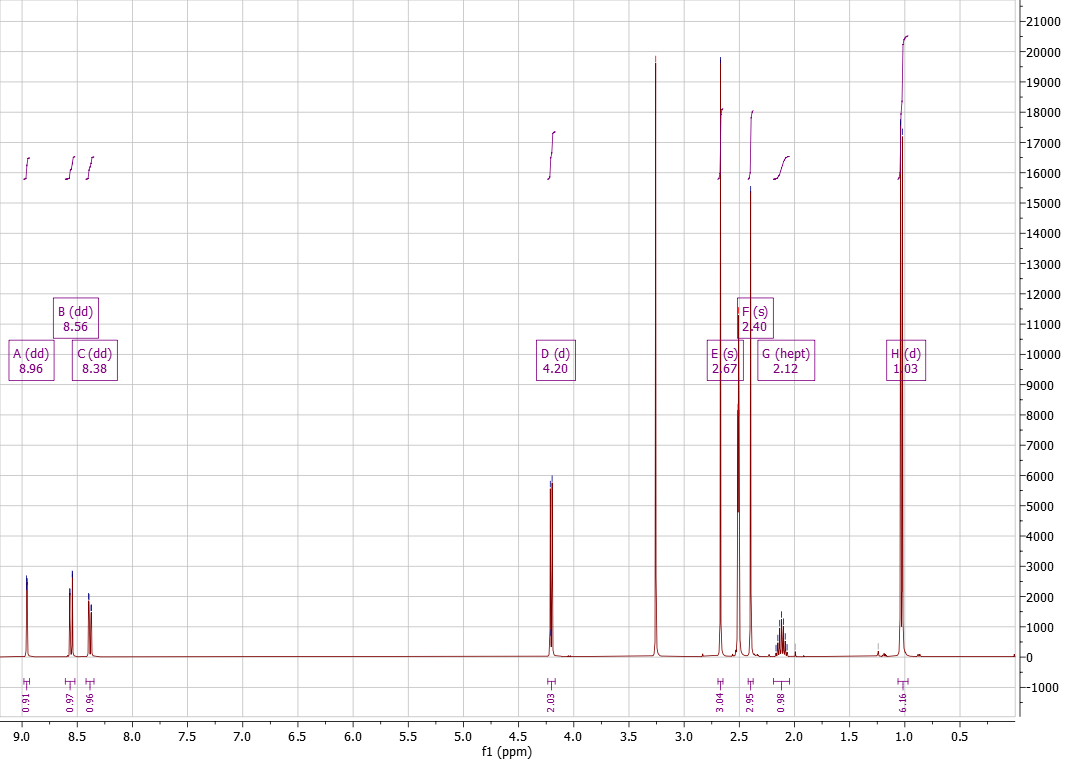


T-155 ^13^C-NMR


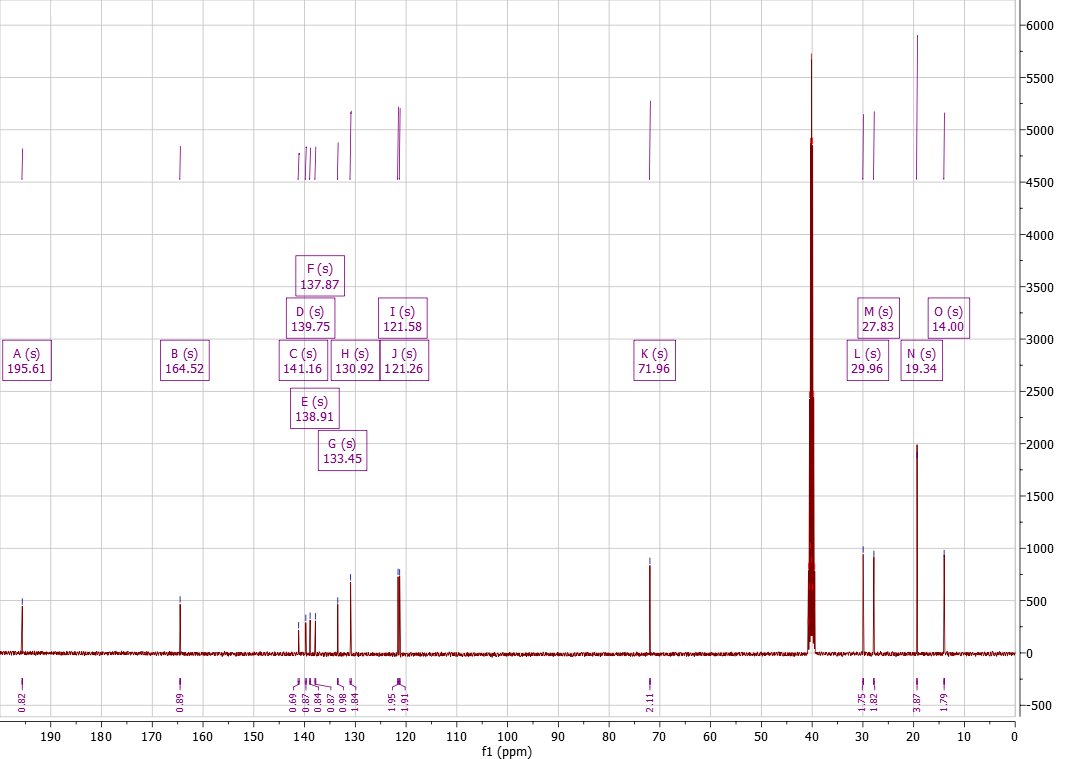


T-155 UPLC-MS


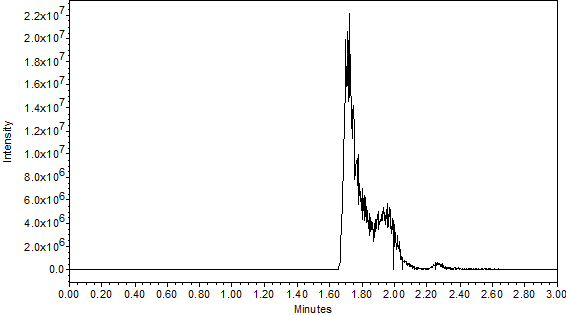


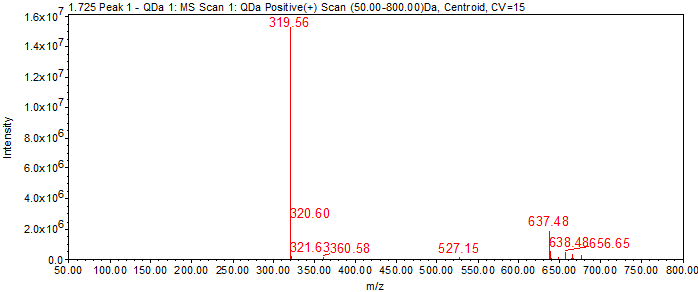


T-156 IR


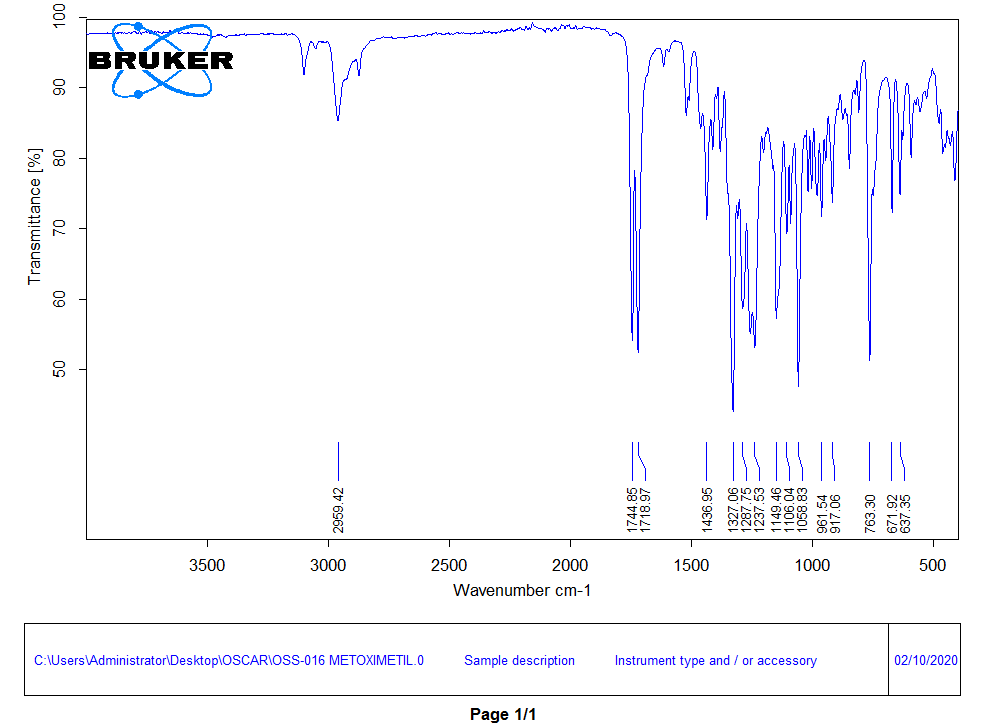


T-156 ^1^H-NMR


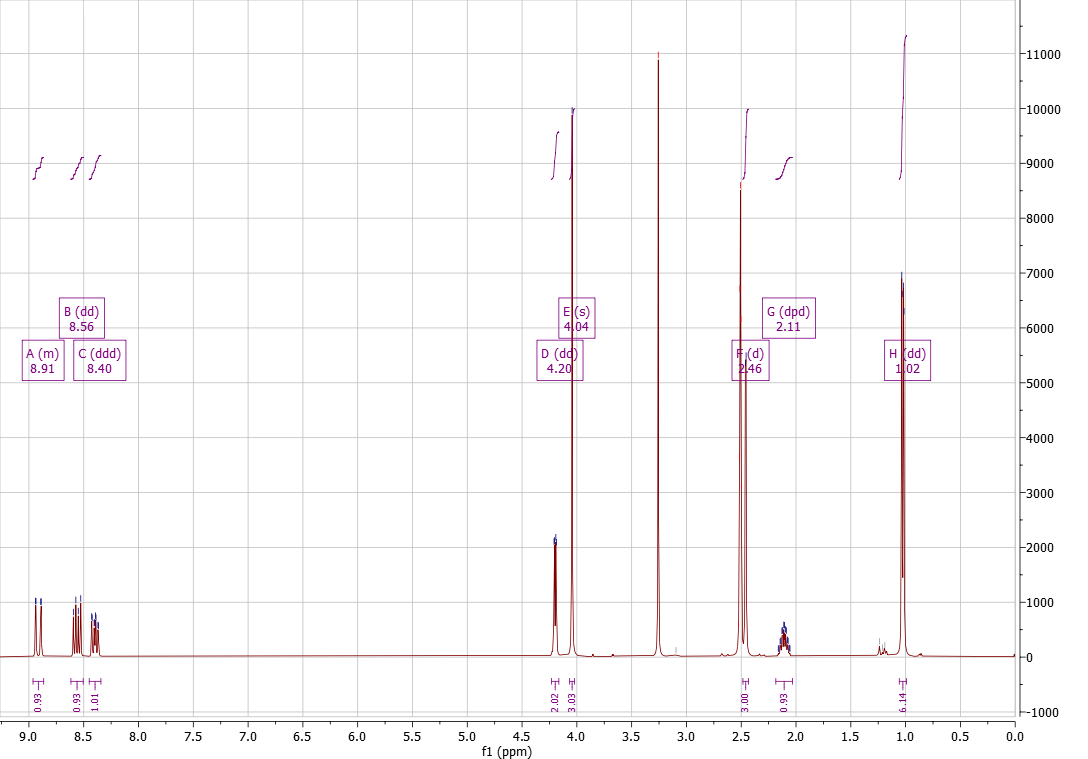


T-156 ^13^C-NMR


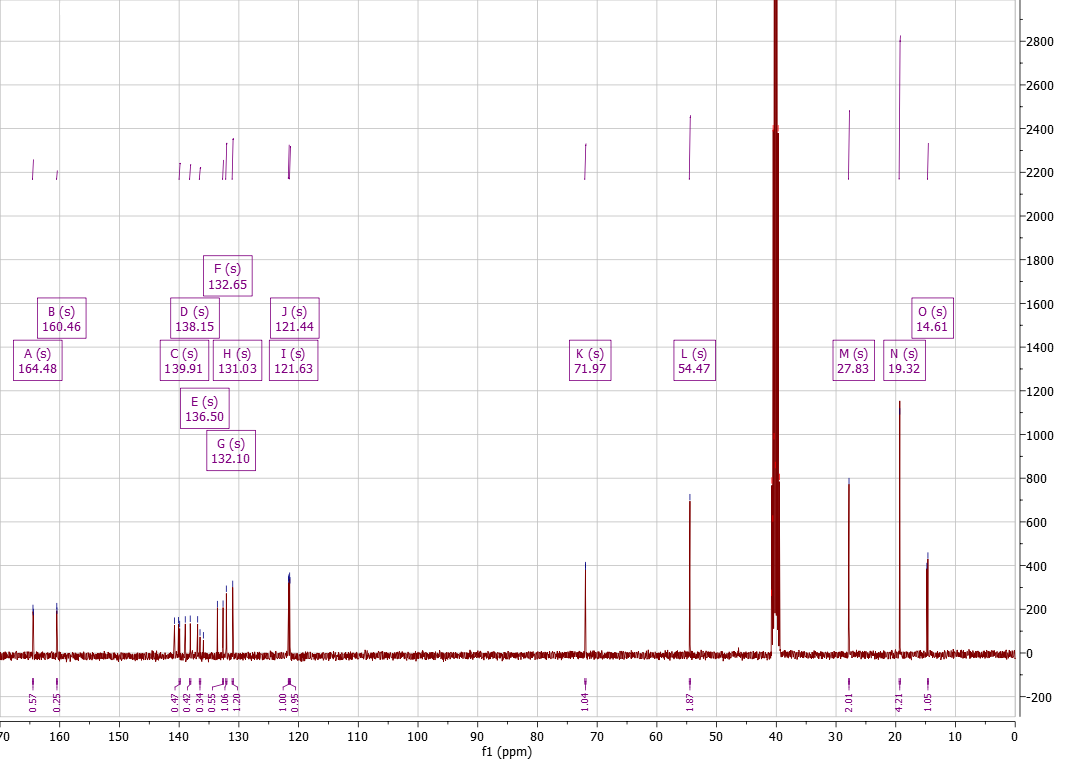


T-156 UPLC-MS


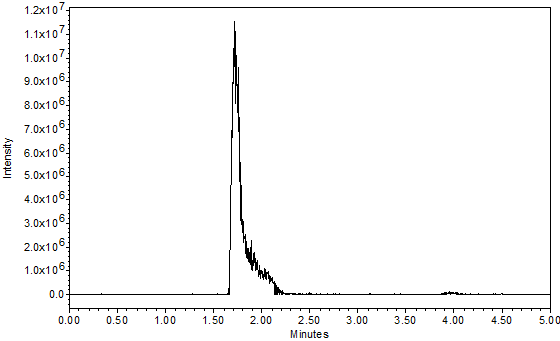


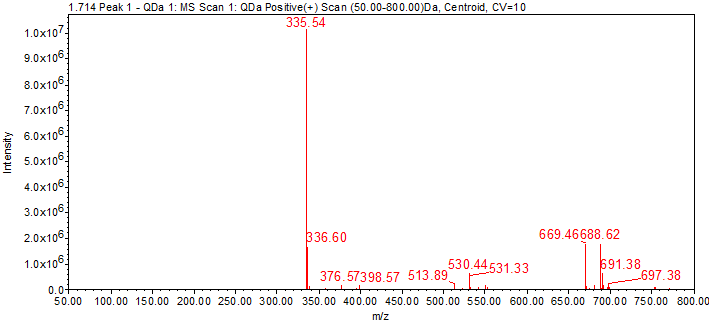


T-157 IR


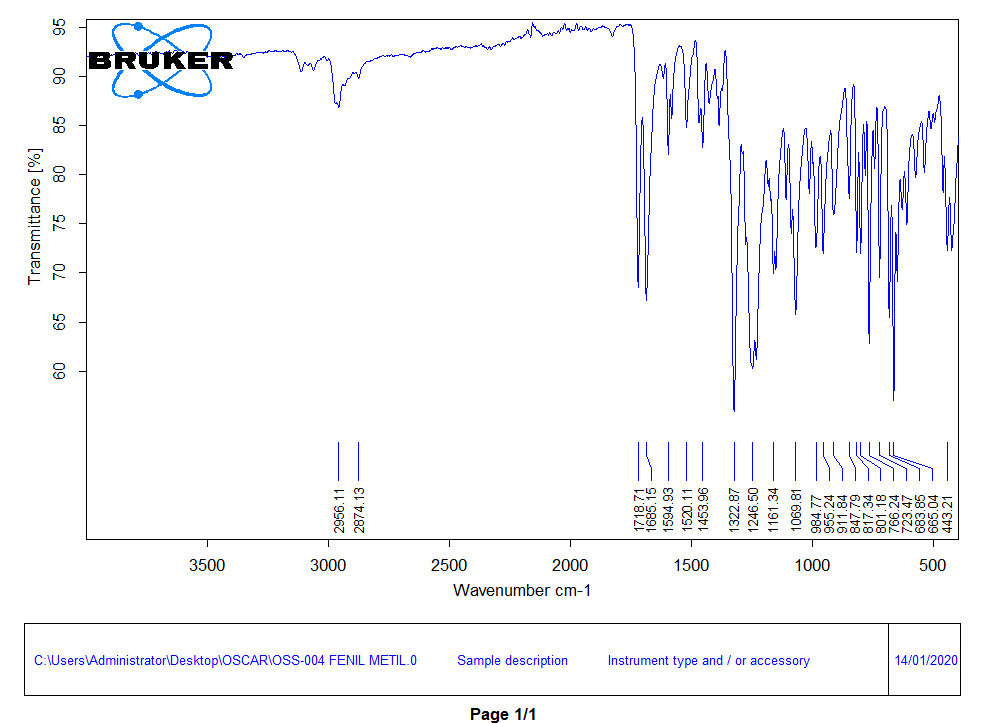


T-157 ^1^H-NMR


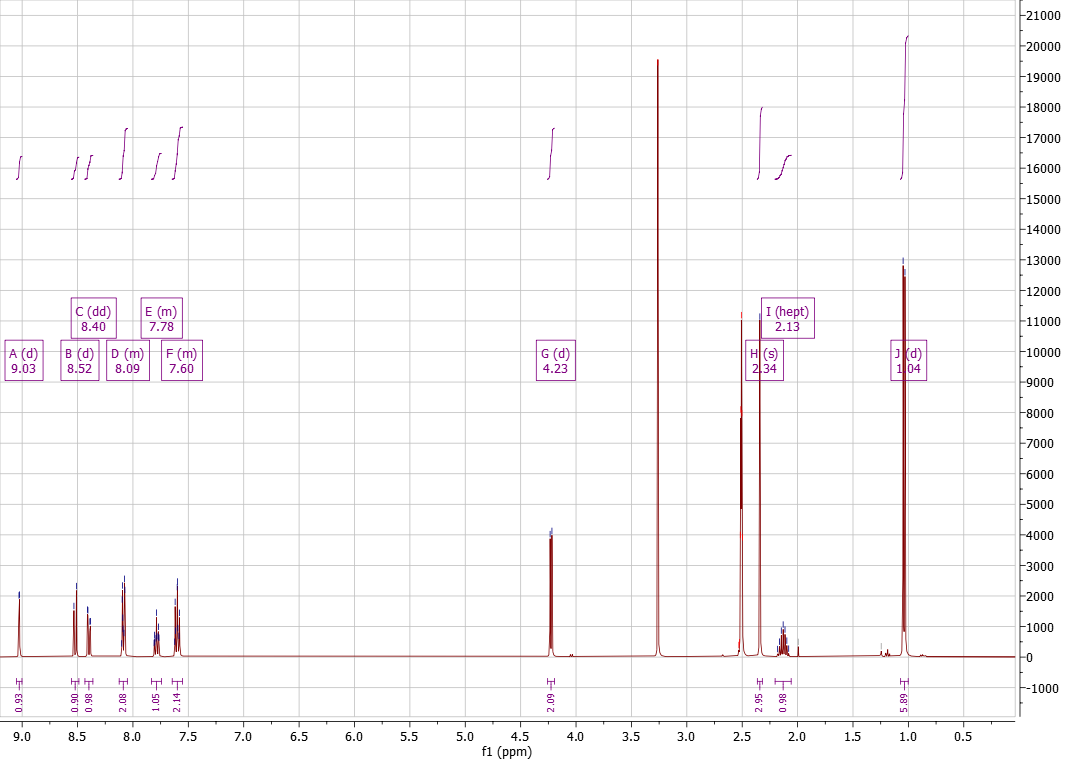


T-157 ^13^C-NMR


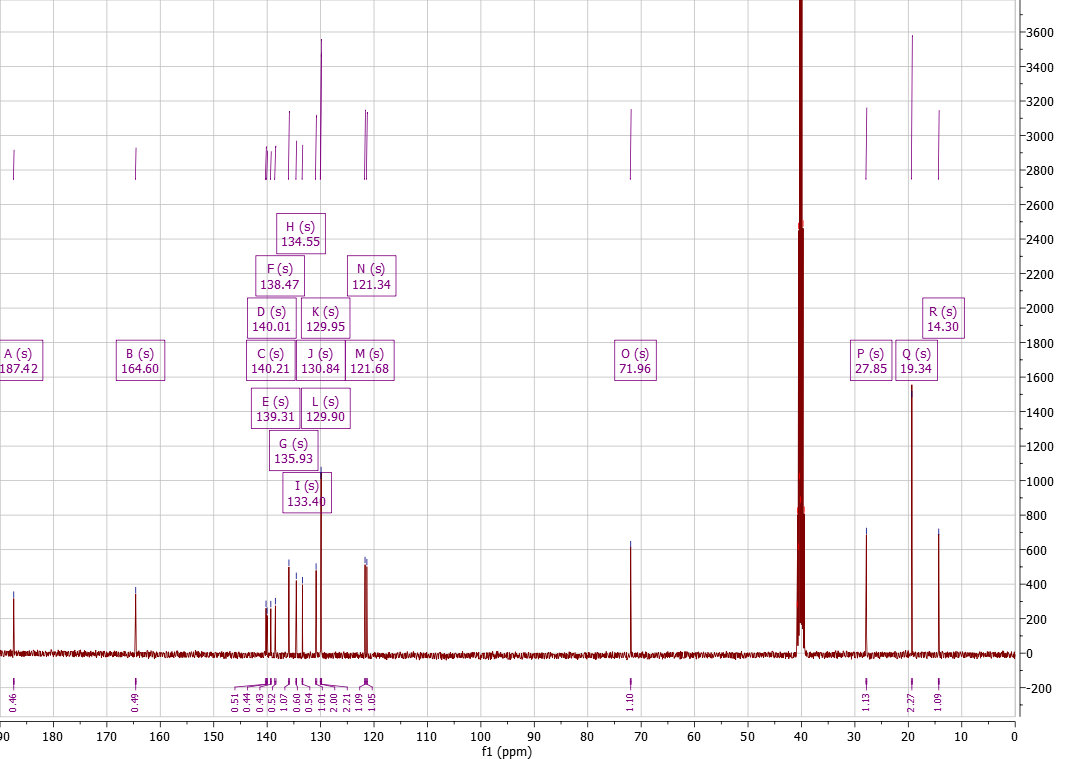


T-157 UPLC-MS


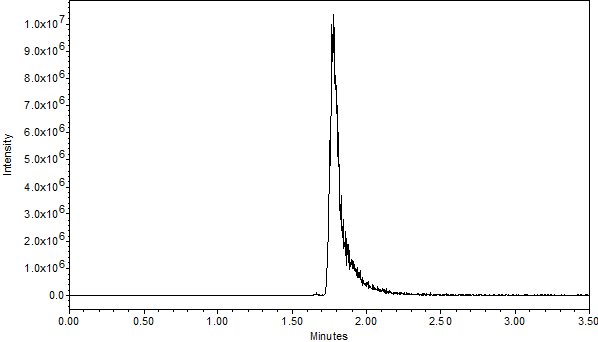


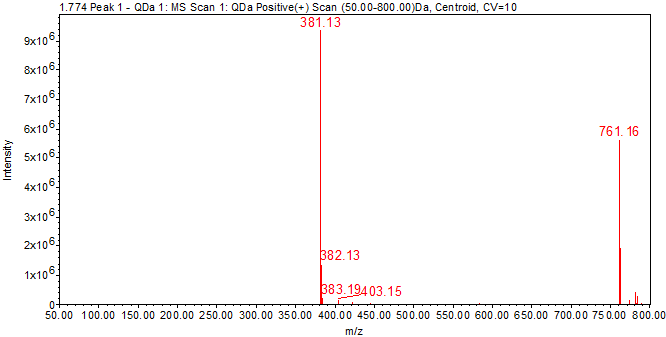


T-158 IR


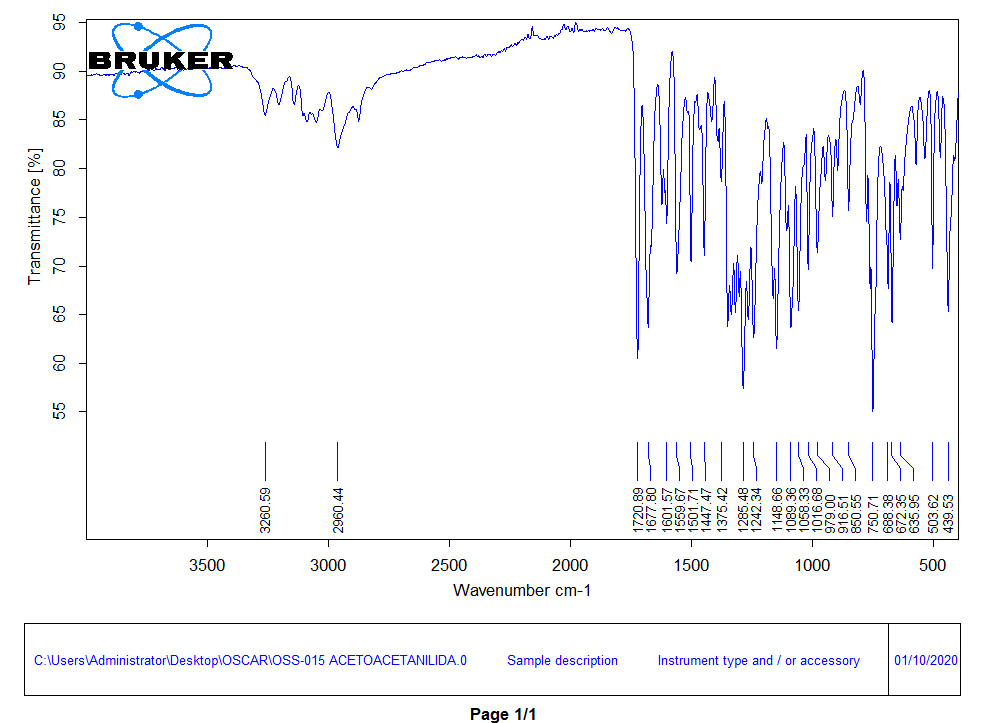


T-158 ^1^H-NMR


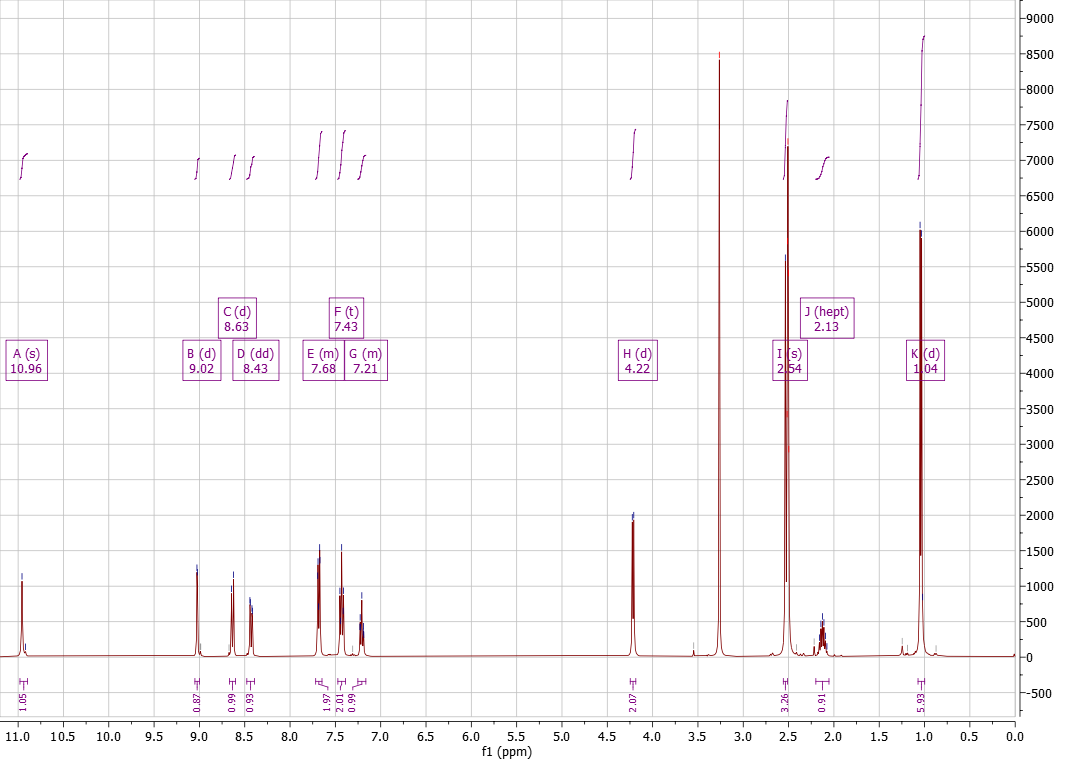


T-158 ^13^C-NMR


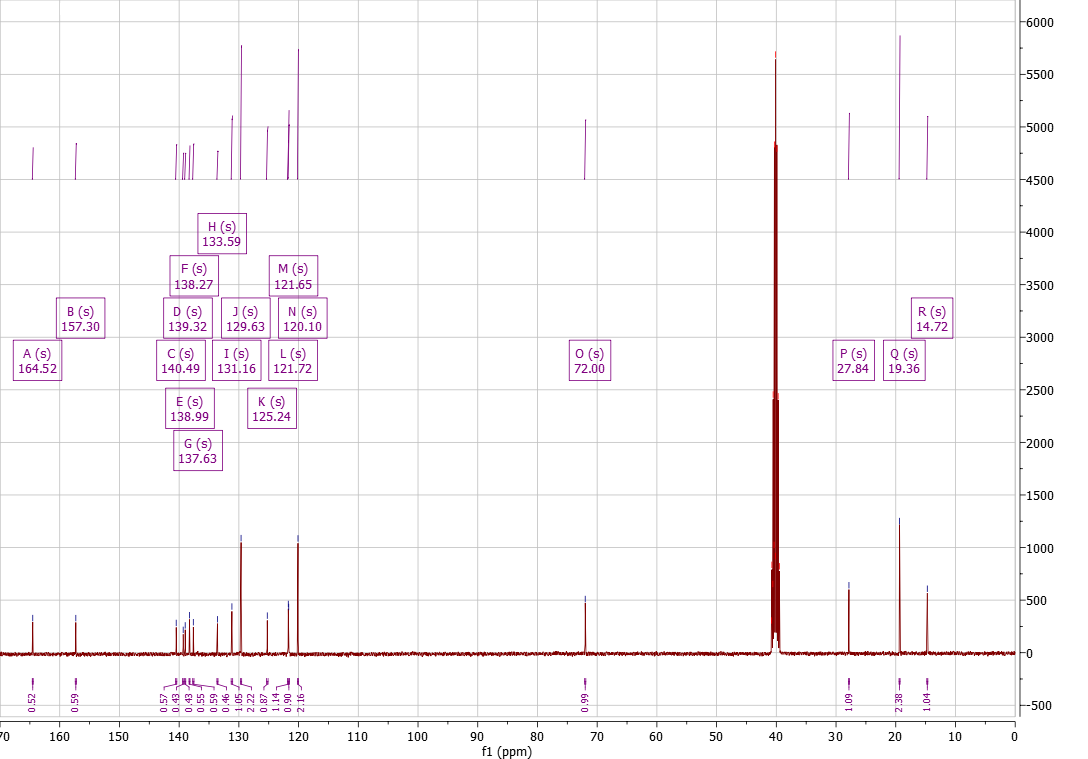


T-158 UPLC-MS


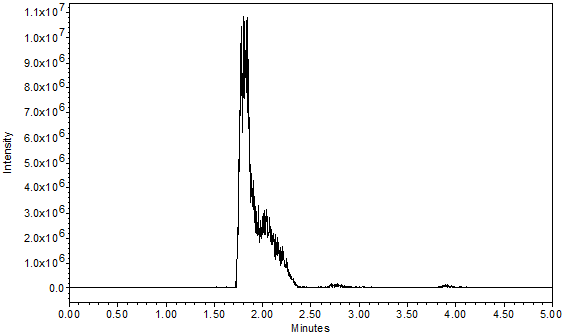


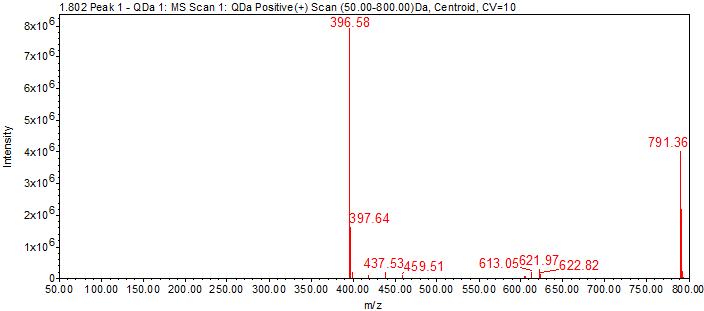


T-159 IR


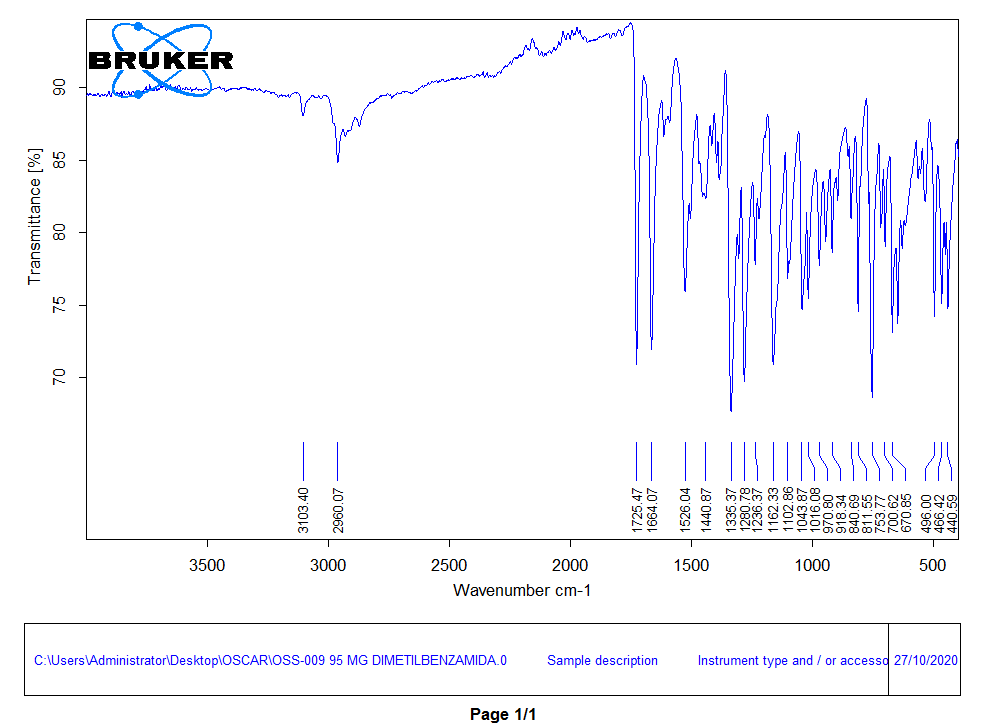


T-159 ^1^H-NMR


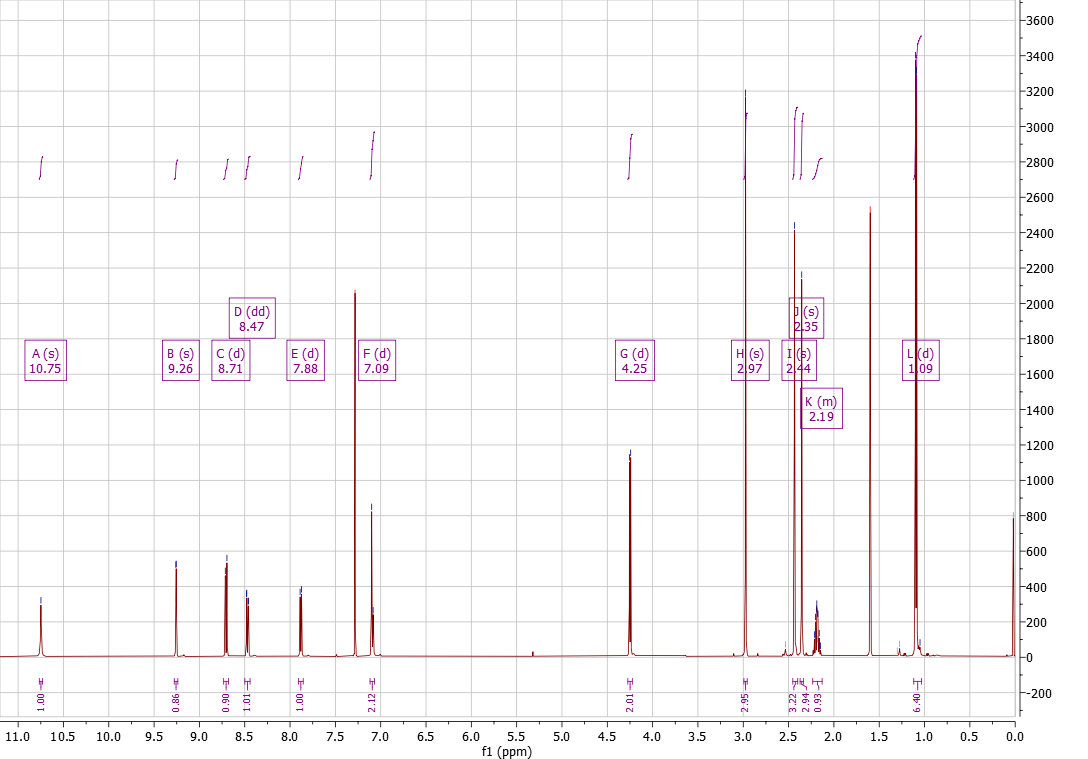


T-159 ^13^C-NMR


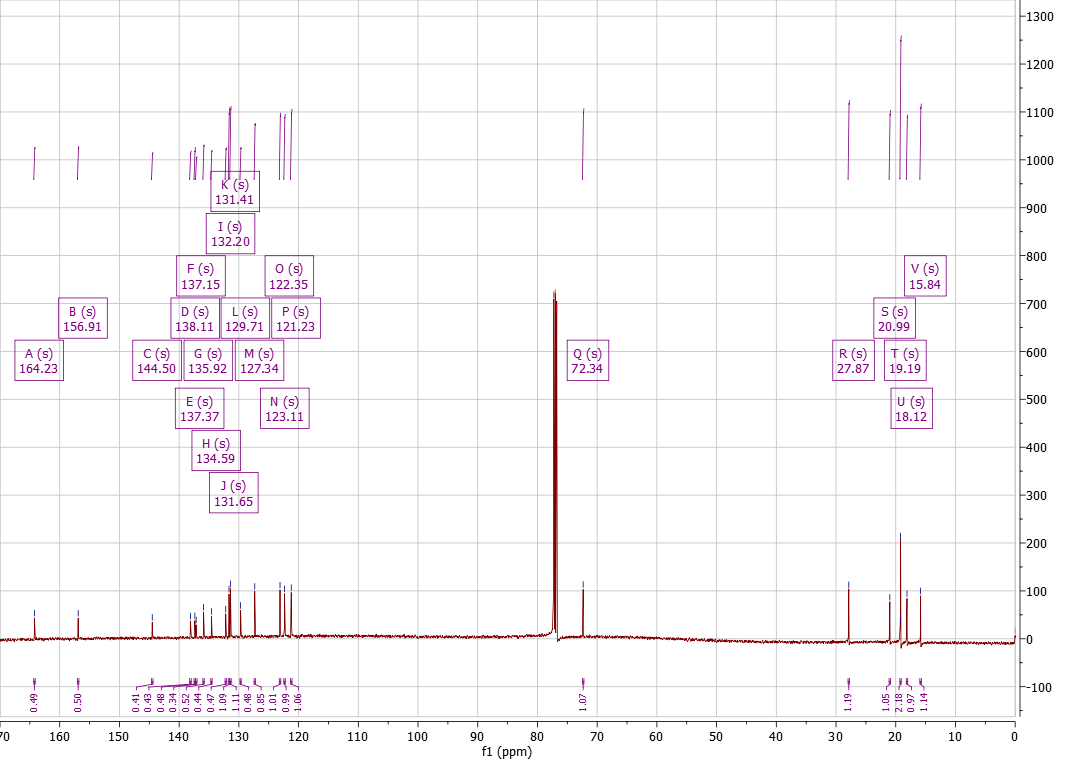


T-161 IR


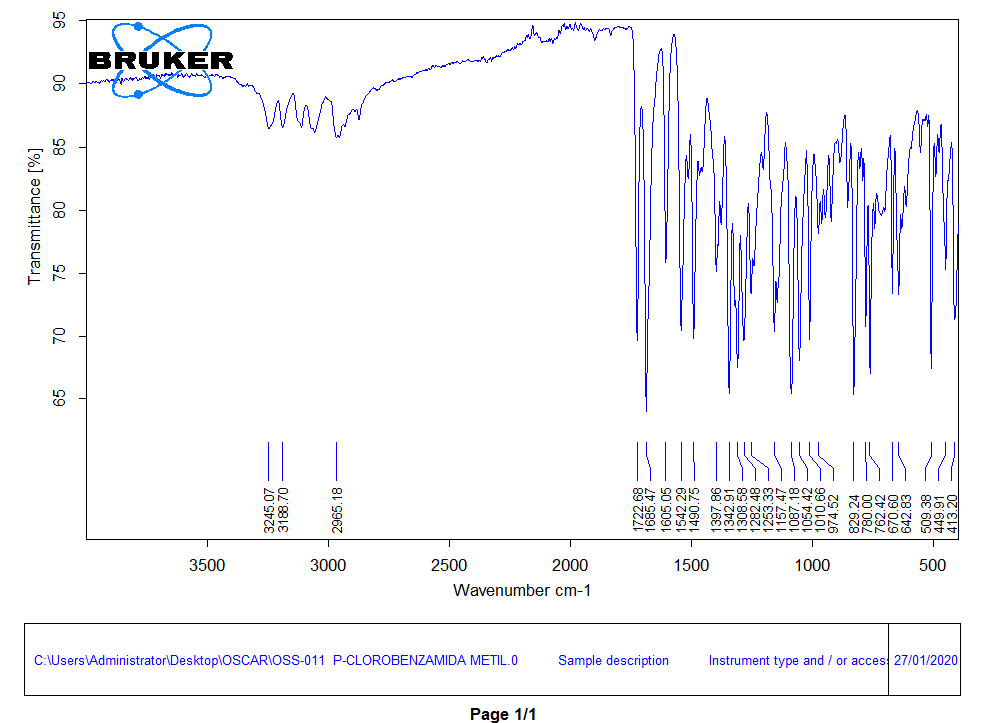


T-161 ^1^H-NMR


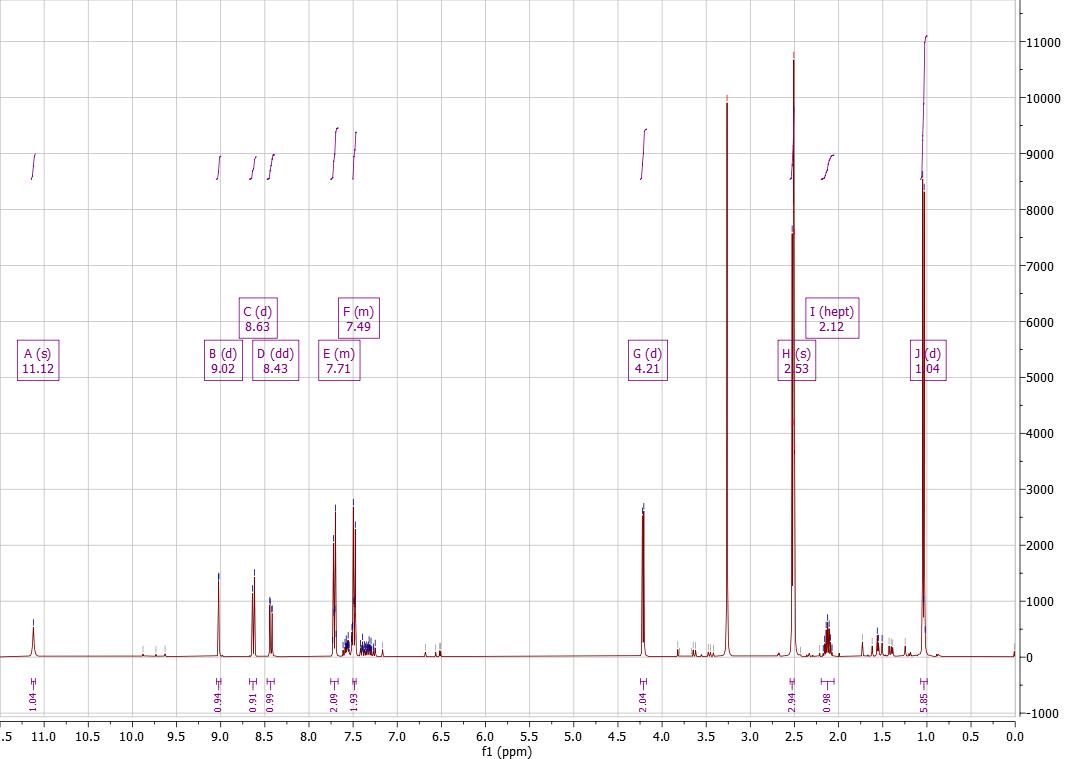


T-161 ^13^C-NMR


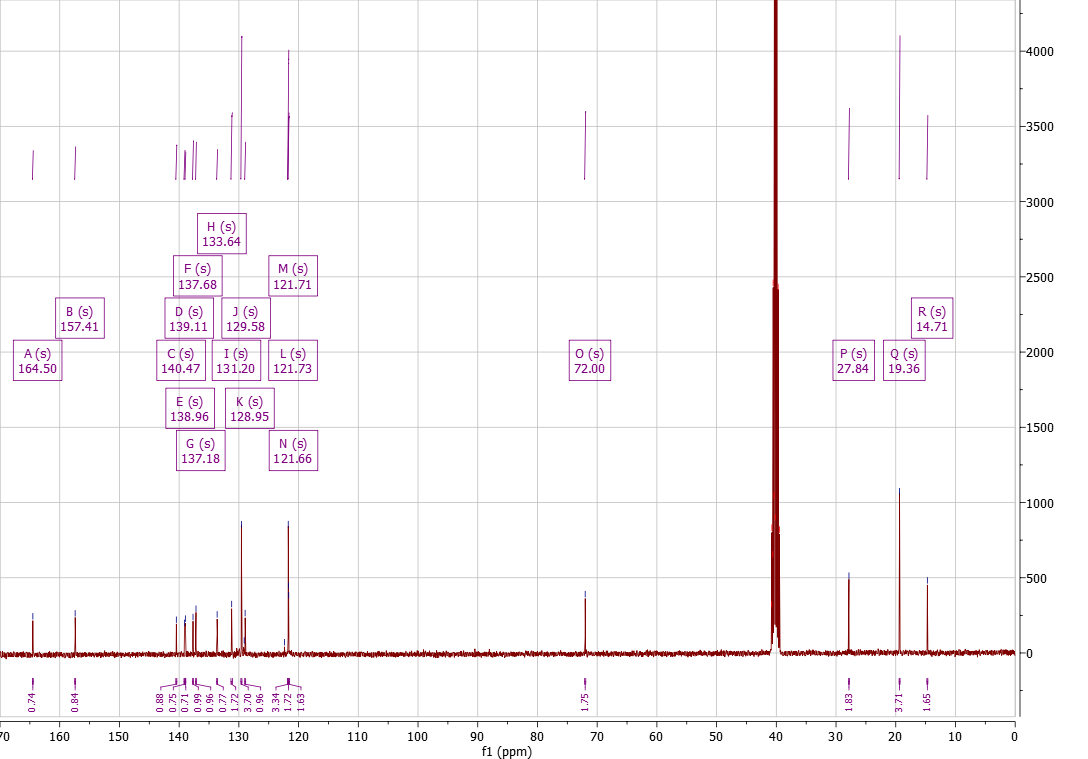


T-161 UPLC-MS


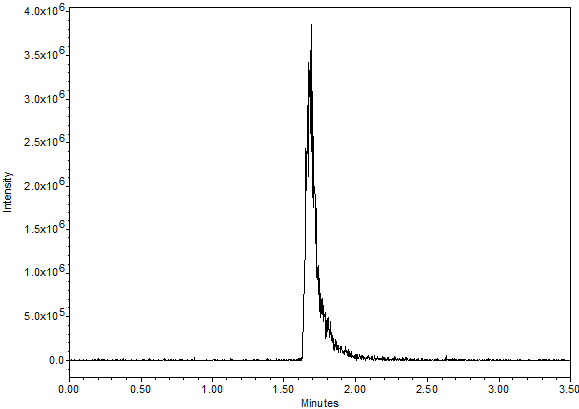


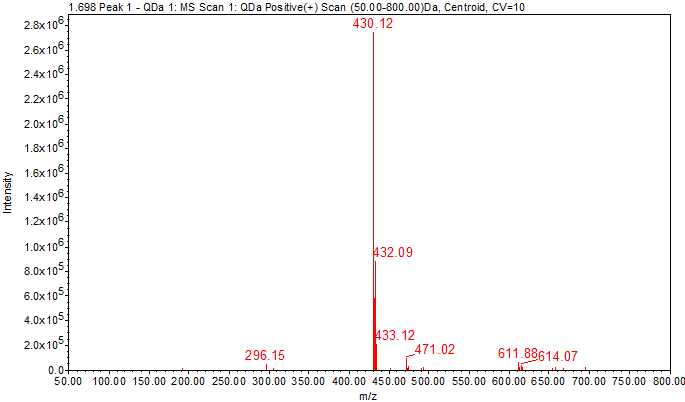


T-163 IR


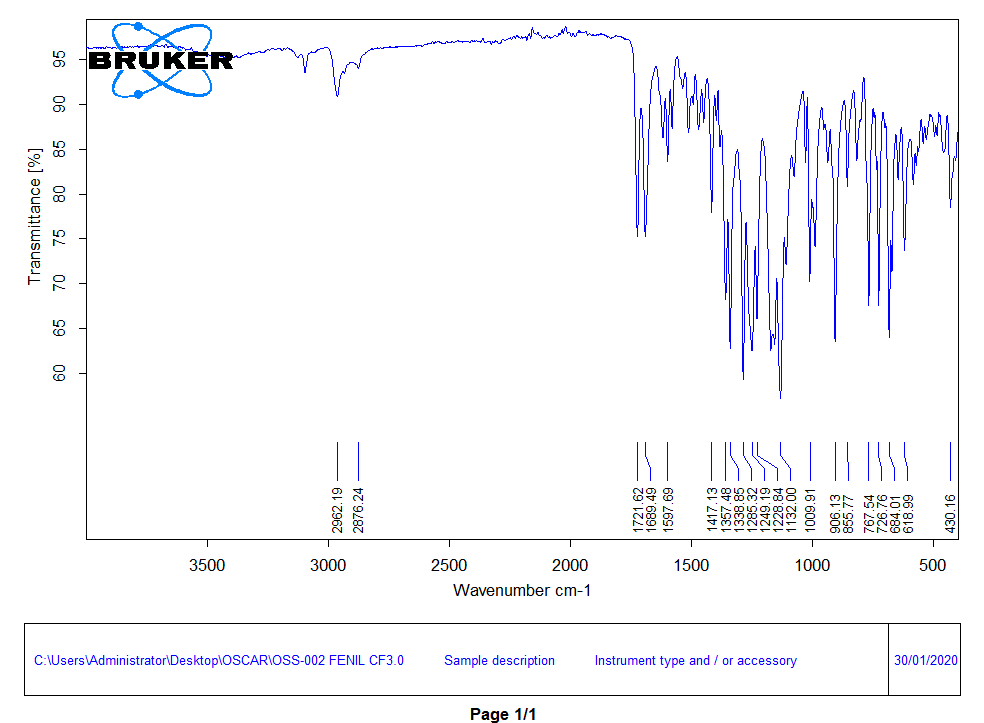


T-163 ^1^H-NMR


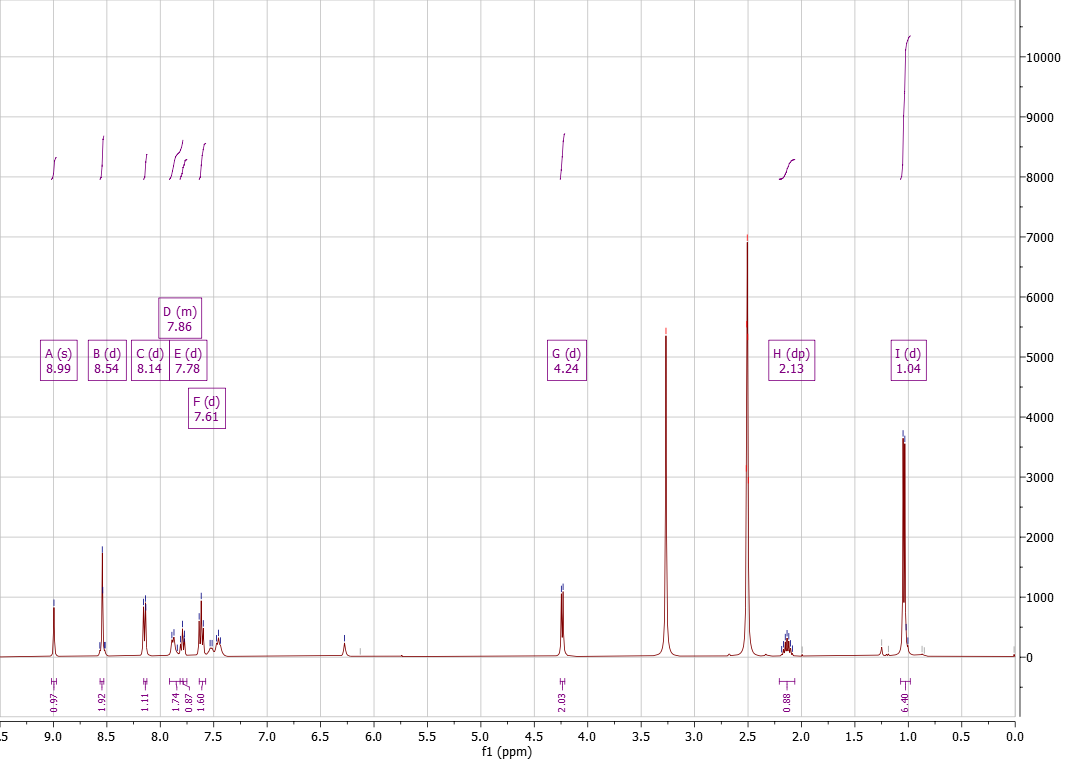


T-163 ^13^C-NMR

T-163 UPLC-MS

T-164 IR

T-164 ^1^H-NMR

T-164 ^13^C-NMR

T-164 UPLC-MS

T-165 IR

T-165 ^1^H-NMR

T-165 ^13^C-NMR

T-165 UPLC-MS

T-166 IR

T-166 ^1^H-NMR

T-166 ^13^C-NMR

T-167 IR

T-167 ^1^H-NMR

T-167 ^13^C-NMR

T-167 UPLC-MS

T-168 IR

T-168 ^1^H-NMR

T-168 ^13^C-NMR

T-168 UPLC-MS

T-169 IR

T-169 ^1^H-NMR

T-169 ^13^C-NMR

T-170 IR

T-170 ^1^H-NMR

T-170 ^13^C-NMR

T-170 UPLC-MS

Molecular docking analysis

Overlapped receptor crystals

**Figure S1**. *Gl*TIM pre-minimization (green), and post-minimization (cyan) RMSD 0.267 Å, active site catalytic diad, and other relevant residues are shown in sticks format.

**Figure S2**. *Tv*TIM pre-minimization (violet), and post-minimization (magneta) RMSD 0.0051Å, active site catalytic diad, and other relevant residues are shown in sticks format.

**Figure S3**. *Eh*TrxR pre-minimization (green), and post-minimization (cyan) RMSD 0.0168Å, NADPH interacting residues are shown in sticks format

*Gl*TIM

**Supplementary Table 1**. BFE and PLIP of *n*-butyl and iso-butyl quinoxaline-7-carboxylate-1,4-di-*N*-oxide derivatives on *G. lamblia*, and human triosephosphate isomerase.

| Code | *Gl*TIM (Active) | | *Gl*TIM (Interface) | | *Gl*TIM (Near C222) | | *Hs*TIM | |
| --- | --- | --- | --- | --- | --- | --- | --- | --- |
|  | BFE kcal/mol | PLIP | BFE kcal/mol | PLIP | BFE kcal/mol | PLIP | BFE kcal/mol | PLIP |
| T-137 | -5.53 | **K13^abd^**, N15^b^, **G176**^b^, **G238**^b^, S216^b^ | -6.86 | Y68^a^, R99^b^, M103^a^, E105^a^, Q109^b^ | -5.34 | S216^b^, N218^b^, G238^b^, A239^b^ | -5.76 | N11^b^ M14^ab^, Q64^a^, N65^a^, **H95^a^**, E97^a^, R98^ab^, V101^a^ F102^a^ |
| T-138 | -5.72 | **K13^abd^**, S216^b^, G237^b^, **G238**^b^ | -7.07 | A45^ab^, Q65^ab^, N66^ab^, T79^b^ | -5.75 | N218^b^, G237^b^, G238^b^, A239^b^, K242^b^ | -5.75 | **K13^d^**, T213^a^, G232^b^, A234^ab^, K237^d^ |
| T-139 | -5.37 | **K13^bd^**, N15^b^, I175^a^ | -7.05 | Y68^a^, L69^a^, R99^d^, M103^a^, Q109^a^ | -5.12 | V180^a^, N218^b^, G238^b^, A239^b^ | -5.68 | **K13^d^**, I170^a^, T213^a^, G232^b^, G233^b^, A234^ab^, K237^d^ |
| T-140 | -5.60 | **K13^ac^**, C14^b^, N15^b^, Q65^a^, L241^a^ | -6.88 | L69^a^, R99^b^, I102^a^, M103^a^, E105^s^, Q109^b^ | -5.70 | I175^a^, N218^b^, G237^b^, G238^b^, A239^b^ | -5.95 | **K13^ac^**, M14^ab^, N15^b^, E97^a^, L236^a^ |
| T-141 | -6.14 | **K13^c^**, Q65^a^, E98^a^, R99^a^, R101^a^, I102^a^, V172^a^, I175^a^ | -7.40 | Y68^a^, L69^a^, R99^b^, M103^a^, E105^s^, Q109^b^ | -5.95 | V180^a^, P183^a^, N218^ab^, N221^ab^, K224^a^, L225^a^, A239^a^ | -6.48 | **K13^ac^**, N15^b^, M14^b^, Q64^a^, **H95^c^**, E97^a^, V101^a^, F102^a^ |
| T-142 | -6.02 | **K13^ab^**, N15^b^, **G176**^a^, G178^b^, **G238**^a^, A239^a^ | -7.73 | A45^a^, A64^a^, Q65^a^, N66^a^, T79^b^, R99^d^ | -5.92 | N218^b^, G237^b^, A239^b^ | -6.52 | **K13**^a^, M14^a^, Q64^a^, E97^a^, R98^d^, V101^a^, F102^a^ |
| T-143 | -6.43 | **K13^ac^**, E98^b^, R99^a^, R101^a^, I102^a^, V172^a^, I175^a^, | -7.37 | Y68^a^, R99^b^, M103^b^, E105^a^ | -5.63 | G176^b^, G178^b^, V180^a^, N218^b^, S220^b^, N221^ab^, A239^a^ | -6.29 | **K13**^ac^, M14^ab^, Q64^a^, E97^a^, R98^d^, F101^a^ |
| T-144 | -6.37 | **K13^ac^**, C14^b^, E98^b^, R99^a^, I102^a^ | -7.92 | A45^a^, E78^b^, T79^b^, R99^b^, M103^a^ | -5.89 | I175^a^, V180^a^, N218^b^, G238^b^, A239^b^ | -6.63 | **K13**^ac^, M14^b^, N15^b^, E97^a^, R98^ad^, F102^a^, |
| T-145 | -5.55 | **K13^ab^**, N15^b^, **G176**^b^, G178^b^, N218^a^, **G238**^a^, A239^a^ | -7.21 | R99^b^, M103^a^, E105^a^, Q109^b^, K113^e^ | -5.32 | Q227^be^, D250^a^, I251^a^, K254^abc^, T255^b^ | -6.11 | **K13**^d^, **H95**^d^, I170^a^, V167^a^, V212^b^ |
| T-146 | -5.78 | **K13^abd^**, N15^b^, **G176**^b^, S216^ae^, **G238**^b^, A239^a^ | -7.28 | R99^b^, I102^a^, M103^a^, E105^a^, Q109^b^, K113^e^ | -5.38 | G176^e^, V180^a^, N218^b^, N221^a^, G238^b^, A239^b^ | -6.22 | **K13**^d^, I170^a^, G209^e^, T213^a^, G232^b^, G233^b^, A234^ab^, K237^d^ |
| T-148 | -5.85 | F20^a^, **G176**^e^, A239^a^, L241^a^, K242^bd^ | -7.69 | A45^a^, A64^a^, Q65^a^, N66^a^, T79^b^, R99^d^ | -5.77 | V180^a^, N218^b^, N221^a^ | -6.32 | **K13**^a^, M14^b^, Q64^a^, R98^ad^, E97^a^, V101^a^, F102^a^ |
| T-149 | -5.92 | **K13^b^**, N15^b^, E98^a^, V172^a^, I175^a^, **G238**^b^ | -7.33 | A45^a^, A64^a^, Y68^a^, T79^b^, R99^d^ | -5.58 | Q227^b^, D250^a^, I251^a^, K254^abc^, T255^b^ | -5.98 | **K13**^a^, M14^ab^, Q64^a^, E97^a^, F102^a^ |
| T-150 | -6.78 | **K13^acd^**, C14^b^, N15^b^, Q65^a^, R99^a^ | -8.08 | Y68^ac^, L69^a^, R99^bd^, I102^a^, E105^b^, Q109^a^, K113^bd^ | -6.73 | I175^a^, V180^a^, N218^b^, N221^a^, G237^b^, A239^b^ | -7.01 | **K13**^acd^, M14^ab^, N15^b^, Q64^a^ |
| T-151 | -6.15 | **K13^ab^**, N15^b^, **G176**^b^, G178^a^, N218^a^, **G238**^b^, A239^a^, L241^a^ | -7.67 | Y68^a^_,_ L69^a^, E78^b^, R99^b^, M103^a^ | -6.26 | I175^a^, G178^a^, N218^b^, G237^b^, A239^b^ | -6.48 | **K13**^abc^, M14^a^, N15^b^, E97^b^, V167^a^, I170^a^, G233^b^ |
| T-155 | -6.07 | N11^a^, **K13^d^**, I175^a^, **G176**^b^, S216^b^, L235^a^, K242^b^ | -7.27 | Y68^a^_,_ L69^a^, R99^b^, M103^a^, E105^a^, Q109^ab^ | -5.45 | G176^b^, G178^a^, S216^b^, N218^b^ | -6.11 | **K13**^a^, M14^ab^, Q64^a^, R98^a^, F102^a^, |
| T-156 | -5.69 | **K13^abd^**, G237^b^, **G238**^b^ | -7.23 | Y68^a^_,_ L69^a^, R99^b^, M103^a^, E105^a^, Q109^ab^ | -5.67 | I175^a^, N218^b^, G237^b^, A239^b^, K242^b^ | -5.82 | **K13**^c^, M14^ab^, **H95**^a^, G97^a^, R98^ad^, F102^a^, |
| T-157 | -6.05 | N15^a^, F20^a^, A239^a^, L241^a^, K242^b^ | -8.04 | A45^a^, A64^a^, Q65^a^, N66^a^, Y68^a^, T79^b^, R99^d^ | -5.93 | V180^a^, N218^b^, N221^a^ | -6.74 | **K13**^ac^, M14^b^, N15^b^, Q64^a^, R98^c^, V101^a^, F102^a^, L236^a^ |
| T-158 | -6.41 | **K13**^ab^, N15^b^, G178^b^, N218^a^, **G238**^b^ | -7.68 | Y68^a^_,_ L69^a^, R99^b^, I102^ab^, M103^a^, E105^a^, Q109^ab^ | -6.11 | G178^b^, N218^b^, G237^b^, A239^b^ | -6.46 | **K13**^a^, M14^b^, N15^b^, Q64^a^, E97^a^, R98^ac^, F102^a^, |
| T-159 | -6.79 | **K13**^c^, C14^b^, Q65^a^, N66^a^, E98^ab^, R99^a^, I102^a^, L241^a^ | -8.01 | Y68^a^_,_ L69^a^, R99^b^, I102^ab^, M103^a^, E105^a^, Q109^ab^ | -6.47 | I175^a^, V180^a^, N218^b^, G237^b^, A239^b^ | -6.92 | **K13**^a^, M14^b^, Q64^a^, **H95**^a^, R98^c^, F102^a^, |
| T-161 | -6.40 | **K13**^ab^, N15^b^, G178^b^, N218^a^, **G238**^b^ | -7.79 | Y68^a^_,_ L69^a^, R99^b^, I102^ab^, M103^a^, E105^a^, Q109^ab^ | -6.05 | G178^b^, V180^a^, P183^a^, N218^b^, N221^b^, K224^a^, L225^a^ | -6.54 | **K13**^a^, M14^b^, N15^b^, E97^a^, R98^ac^, F102^a^ |
| T-163 | -5.93 | N15^a^, F20^a^, L241^a^, K242^b^ | -8.00 | A45^a^, Q65^a^, N66^a^, Y68^a^, T79^b^, R99^d^ | -5.97 | V180^a^, N218^b^, N221^a^ | -6.61 | **K13**^a^, M14^b^, Q64^a^, **H95**^a^, E97^a^, R98^d^, F102^a^ |
| T-164 | -6.02 | **K13**^abc^, N15^b^, G178^b^, N218^a^, **G238**^b^ | -7.38 | A45^a^, Q65^a^, N66^a^, Y68^a^, T79^b^, R99^d^ | -5.71 | S216^e^, N218^a^, G219^b^, S220^b^, E244^a^ | -6.21 | M14^b^, Q64^a^, **H95**^a^, E97^a^, R98^ad^, F102^a^ |
| T-165 | -6.32 | **K13**^ab^, N15^b^, G178^b^, N218^a^, **G238**^b^ | -7.51 | A45^a^, N66^a^, Y68^a^, T79^b^, R99^d^ | -6.06 | N218^b^, G237^b^, A239^b^, K242^b^ | -6.34 | **K13**^c^, G171^b^, T172^b^, G173^b^, G232^b^, G233^b^ |
| T-166 | -6.77 | **K13**^b^, F20^a^, **G176**^b^, G178^b^, N218^a^, L241^a^ | -7.59 | R99^bce^, M103^a^, E105^a^, T106^a^ | -6.64 | K13^ad^, G176^b^, S216^b^, N218^b^, G237^b^, G238^b^, E244^e^ | -7.53 | **K13**^ac^, M14^b^, N15^b^, Q64^a^, F102^a^, L236^a^ |
| T-167 | -6.15 | **K13**^abd^, N15^b^, **G176**^b^, S216^b^, **G238**^b^ | -7.86 | L69^a^, R99^b^, M103^a^, E105^a^, Q109^ab^ | -5.35 | I175^a^, N218^b^, G237^b^, G238^b^, A239^b^ | -6.12 | G209^e^, T213^a^, G232^b^, G233^b^, A234^ab^, L237^d^ |
| T-168 | -6.14 | **K13**^abd^, N15^b^, G178^b^, N218^a^, **G238**^b^, A239^a^ | -7.78 | Y68^a^, R99^b^, I102^a^, E105^a^, K113^c^ | -6.22 | K13^ad^, G176^b^, S216^b^, N218^b^ | -6.38 | A11^a^ **K13**^d,^ E165^a^, I170^a^, T213^a^, L230^a^, G232^b^, G233^b^, A234^ab^ |
| T-169 | -6.68 | **K13**^abd^, N15^b^, I175^a^, **G176**^b^, S216^b^ | -8.19 | Y68^a^, L69^a^, R99^b^, M103^ab^, E105^a^, T106^a^, Q109^a^, K113^d^ | -6.17 | I175^a^, G237^b^, A239^b^, K242^ab^ | -6.74 | **K13**^ab^. M14^ab^, N15^b^, Q64^a^, R98^a^, F102^a^ |
| T-170 | -6.01 | **K13**^b^, N15^b^, E98^a^, R101^a^, I102^a^, I175^a^, L241^a^ | -8.01 | Y68^a^, L69^a^, R99^b^, I102^a^, M103^a^, E105^a^, Q109^b^, K113^d^ | -5.56 | N218^b^, S220^b^, A239^a^, K242^d^, P243^a^, T247^a^ | -6.32 | **K13**^d^, **H95**^d^, S96^b^, I170^a^, V212^ab^, T213^a^, V231^a^, G232^b^, A234^ab^, K237^c^ |
| Ome |  |  |  |  | -6.08 | G215^b^, S216^b^_,_ A217^b^, N218^b^, G237^b^, A239^b^_,_ E244^d^ |  |  |

*Tv*TIM

**Supplementary Table 2**. BFE and PLIP of *n*-butyl and iso-butyl quinoxaline-7-carboxylate-1,4- di-*N*-oxide derivatives on *T. vaginalis*, and human triosephosphate isomerase

| Code | *Tv*TIM | | *Hs*TIM | |
| --- | --- | --- | --- | --- |
|  | BFE  kcal/mol | PLIP | BFE  kcal/mol | PLIP |
| T-137 | -6.33 | **H94^a^,** F44^ab^, V45^b^, I91^a^, R97^ad^ | -5.76 | N11^b^ M14^ab^, Q64^a^, N65^a^, **H95^a^**, E97^a^, R98^ab^, V101^a^ F102^a^ |
| T-138 | -6.07 | A12^a^, P43^a^, F44^ab^, F46^a^, E63^ab^, E96^a^, R97^ab^ | -5.75 | **K13^d^**, T213^a^, G232^b^, A234^ab^, K237^d^ |
| T-139 | -6.14 | A12^a^, P43^a^, F44^a^, V45^a^, F46^a^, E63^a^, N64^a^, R97^ad^ | -5.68 | **K13^d^**, I170^a^, T213^a^, G232^b^, G233^b^, A234^ab^, K237^d^ |
| T-140 | -6.49 | A12^ab^, P43^a^, F44^b^, **H94^a^**, E96^ab^, R97^b^, L101^a^ | -5.95 | **K13^ac^**, M14^ab^, N15^b^, E97^a^, L236^a^ |
| T-141 | -7.20 | P43^a^, F44^b^, V45^a^, F46^a^, E63^a^, I91^a^, **H94^a^**, E96^b^, R97^a^, L101^a^ | -6.48 | **K13^ac^**, N15^b^, M14^b^, Q64^a^, **H95^c^**, E97^a^, V101^a^, F102^a^ |
| T-142 | -7.44 | A12^a^, P43^a^, F44^ab^, V45^a^, F46^a^, E63^a^, I91^a^, **H94^a^**, R97^d^ | -6.52 | **K13**^a^, M14^a^, Q64^a^, E97^a^, R98^d^, V101^a^, F102^a^ |
| T-143 | -6.90 | A12^a^, P43^a^, F44^b^, F46^a^, E63^a^, I91^a^, **H94^a^**, E96^b^, I101^a^ | -6.29 | **K13**^ac^, M14^ab^, Q64^a^, E97^a^, R98^d^, F101^a^ |
| T-144 | -7.09 | A12^b^, F44^ab^, V45^ab^, E63^a^**, H94^a^**, R97^ad^ | -6.63 | **K13**^ac^, M14^b^, N15^b^, E97^a^, R98^ad^, F102^a^, |
| T-145 | -6.61 | P43^a^, F44^ab^, V45^ab^, F46^a^, E63^b^, E96^e^, R97^b^ | -6.11 | **K13**^d^, **H95**^d^, I170^a^, V167^a^, V212^b^ |
| T-146 | -7.09 | A12^a^, P43^a^, F44^b^, V45^b^, F46^a^, E63^ab^_,_ **H94^a^**, E96^e^, R97^d^ | -6.22 | **K13**^d^, I170^a^, G209^e^, T213^a^, G232^b^, G233^b^, A234^ab^, K237^d^ |
| T-148 | -7.44 | **K11^a^**, A12^ab^, P43^a^, F44^b^, V45^ab^, E63^a^, **H94^a^**, E96^b^, R97^ab^, | -6.32 | **K13**^a^, M14^b^, Q64^a^, R98^ad^, E97^a^, V101^a^, F102^a^ |
| T-149 | -7.02 | **K11^a^**, A12^a^, P43^a^, F44^ab^, V45^ab^, F46^a^, E63^abe^, R97^b^ | -5.98 | **K13**^a^, M14^ab^, Q64^a^, E97^a^, F102^a^ |
| T-150 | -8.07 | **K11^a^**, P43^a^, F44^b^, V45^b^, F46^a^, E63^ab^, E96^a^, R97^b^, I100^a^ | -7.01 | **K13**^acd^, M14^ab^, N15^b^, Q64^a^ |
| T-151 | -7.42 | A12^ab^, K15^a^, P43^a^, F44^ab^, V45^ab^, E63^a^, **H94^a^**, R97^ad^ | -6.48 | **K13**^abc^, M14^a^, N15^b^, E97^b^, V167^a^, I170^a^, G233^b^ |
| T-155 | -6.32 | A12^a^, P43^a^, F44^ab^, V45^ab^, F46^a^, E63^ab^, R97^ab^ | -6.11 | **K13**^a^, M14^ab^, Q64^a^, R98^a^, F102^a^, |
| T-156 | -6.32 | A12^a^, P43^a^, F44^ab^, V45^ab^, F46^a^, E63^ab^, R97^ab^ | -5.82 | **K13**^c^, M14^ab^, **H95**^a^, G97^a^, R98^ad^, F102^a^, |
| T-157 | -7.55 | **K11^a^**, P43^a^, F44^ab^, V45^a^, F46^a^, E63^ab^, E96^a^, R97^ab^, I100^a^ | -6.74 | **K13**^ac^, M14^b^, N15^b^, Q64^a^, R98^c^, V101^a^, F102^a^, L236^a^ |
| T-158 | -7.22 | **K11^a^**, A12^a^, F44^ab^, **H94^a^**, R97^ad^ | -6.46 | **K13**^a^, M14^b^, N15^b^, Q64^a^, E97^a^, R98^ac^, F102^a^, |
| T-159 | -7.17 | **K11^a^**, P43^a^, E63^b^, E96^a^, R97^a^ | -6.92 | **K13**^a^, M14^b^, Q64^a^, **H95**^a^, R98^c^, F102^a^, |
| T-161 | -7.20 | **K11^a^**, F44^ab^, I91^a^, **H94^a^**, R97^d^ | -6.54 | **K13**^a^, M14^b^, N15^b^, E97^a^, R98^ac^, F102^a^ |
| T-163 | -7.64 | **K11^a^**, P43^a^, F44^ab^, V45^ab^, F46^a^, E63^ab^, E96^ae^, R97^b^ | -6.61 | **K13**^a^, M14^b^, Q64^a^, **H95**^a^, E97^a^, R98^d^, F102^a^ |
| T-164 | -7.36 | **K11^a^**, A12^a^, F44^b^, I91^a^, **H94^a^**, R97^d^ | -6.21 | M14^b^, Q64^a^, **H95**^a^, E97^a^, R98^ad^, F102^a^ |
| T-165 | -7.33 | **K11^a^**, A12^a^, F44^b^, I91^a^, **H94^a^**, R97^d^ | -6.34 | **K13**^c^, G171^b^, T172^b^, G173^b^, G232^b^, G233^b^ |
| T-166 | -8.68 | **K11^a^**, E96^a^, P43^a^, F44^ab^, V45^ab^, F46^a^, E63^b^, R97^b^, I100^a^ | -7.53 | **K13**^ac^, M14^b^, N15^b^, Q64^a^, F102^a^, L236^a^ |
| T-167 | -6.88 | A12^a^, P43^a^, F44^ab^, V45^ab^, F46^a^, E63^b^, E96^e^, R97^b^ | -6.12 | G209^e^, T213^a^, G232^b^, G233^b^, A234^ab^, L237^d^ |
| T-168 | -7.94 | **K11^a^**, P43^a^, F44^ab^, V45^a^, F46^a^, E63^a^, **H94^a^**, R97^d^ | -6.38 | A11^a^ **K13**^d,^ E165^a^, I170^a^, T213^a^, L230^a^, G232^b^, G233^b^, A234^ab^ |
| T-169 | -7.41 | **K11^a^**, A12^a^, Pro43^a^, V45^a^, E63^ab^, **H94^a^**, E96^b^, R97^a^ | -6.74 | **K13**^ab^. M14^ab^, N15^b^, Q64^a^, R98^a^, F102^a^ |
| T-170 | -7.83 | **K11^a^**, A12^a^, P43^a^, F44^a^, V45^a^, F46^a^, E63^ab^, **H94^a^**, R97^d^ | -6.32 | **K13**^d^, **H95**^d^, S96^b^, I170^a^, V212^ab^, T213^a^, V231^a^, G232^b^, A234^ab^, K237^c^ |
| EQX-20 | -6.46 | F44^a^, E63^ab^, E96^a^, R97^b^, L101^a^ | -5.76 | **K13^bd^, H95^d^, I170^a^, G233^b^** |

*Eh*TrxR

**Supplementary Table 3.** BFE and PLIP of *n*-butyl and iso-butyl quinoxaline-7-carboxylate-1,4-di-*N*-oxide derivatives on *E. histolytica* thioredoxin reductase.

| Code | *Eh*TrxR [NADPH] | | *Eh*TrxR [Active] | |
| --- | --- | --- | --- | --- |
|  | BFE  kcal/mol | PLIP | BFE  kcal/mol | PLIP |
| T-137 | -6.73 | **G160**^b^, **A163**^a^, **H182**^d^, **R183**^abc^, R188^d^, E210^a^, I246^a^ | -6.36 | T50^b^, Q64^b^, A121^a^, **A163**^a^ |
| T-138 | -6.64 | V158^a^, **G160**^b^, G161^b^, **R183**^acd^, R188^d^, I246^a^ | -6.19 | T50^b^, I142^a^, **A163**^a^, E166^a^, G167^a^, H170^a^ |
| T-139 | -6.73 | **K122**^ad^, **G160**^b^, **H182**^d^, **R183**^acd^ | -6.15 | W133^a^, A139^ab^ |
| T-140 | -7.00 | **K122**^ad^, **G160**^b^, **H182**^d^, **R183**^acd^ | -6.57 | Q46^a^, T50^b^, I142^a^, **A163**^a^, E166^a^, E167^b^, H170^a^ |
| T-141 | -7.42 | **K122**^ad^, **H182**^d^, **R183**^acd^ | -7.09 | V41^a^, T49^a^, A121^a^, W133^a^, Q134^b^, A139^ab^, **C140^b^**, V147^a^, |
| T-142 | -7.10 | G159^b^, **G160**^b^, G161^b^, **A163**^a^, **R183**^bcd^, E210^a^, I246^a^ | -7.24 | Q46^a^, L47^a^, T50^b^, I142^a^, **A163**^a^, E166^a^, E167^b^, H170^a^ |
| T-143 | -7.66 | **K122**^d^, V158^a^, **R183**^ab^, I246^a^, H248^a^ | -7.74 | T49^b^, T50^b^, T51^a^, I142^a^, E166^a^, H170^a^, |
| T-144 | -8.08 | V158^a^, **G160**^b^, G161^b^, **A163**^b^, **R183**^ab^, A188^a^, E210^a^, I246^a^ | -7.76 | Q46^a^, T49^b^, T50^b^, I142^a^, E166^a^ |
| T-145 | -7.03 | **A163**^a^, **R183**^abc^, A188^b^, I246^a^ | -6.58 | V41^a^, T49^a^, A121^a^, W133^a^, A139^b^ |
| T-146 | -7.00 | **A163**^a^, **R183**^abcd^, A188^b^, I246^a^ | -6.32 | T49^b^, **A163**^e^ |
| T-148 | -7.32 | Q46^b^, T50^b^, A121^a^, **A163**^a^ | -7.36 | Q46^a^, L47^a^, T50^b^, I142^a^, **A163**^a^, E166^a^, E167^b^, H170^a^ |
| T-149 | -7.07 | **K122**^e^, G159^b^, **G160**^b^, G161^b^, **A163**^a^, **H182**^d^, **R183**^bcd^, E210^a^ | -6.94 | T50^b^, A121^a^, I142^a^, **A163**^a^, E166^a^, H170^a^ |
| T-150 | -8.15 | V158^a^, **G160**^b^, G161^b^, D162^b^, **A163**^b^, **R183**^ac^, I246^a^ | -7.45 | Q46^a^, L47^a^, T50^b^, T51^b^, I142^a^, E166^a^, H170^a^ |
| T-151 | -7.46 | **K122**^c^, V158^a^, **G160**^b^, D162^b^, **A163**^a^, **R183**^abc^, I246^a^ | -6.83 | V41^a^, T49^a^, H248^d^ |
| T-155 | -6.91 | **G160**^b^, **A163**^a^, **H182**^d^, **R183**^abc^, R188^d^, E210^a^, I246^a^ | -6.65 | M37^a^, V41^a^, A121^a^, W133^a^, Q134^b^, A139^ab^, **C140**^b^ |
| T-156 | -6.87 | **K122**^ad^, **G160**^b^, **H182**^d^, **R183**^acd^, I246^a^ | -6.60 | M37^a^, V41^a^, W133^a^, Q134^b^, A139^ab^, **C140**^b^ |
| T-157 | -7.30 | V158^a^, **G160**^b^, G161^b^, **A163**^b^, **R183**^ab^, R188^ac^, I246^a^ | -7.28 | Q46^a^, L47^a^, T50^b^, **A163**^a^, E166^a^ |
| T-158 | -7.76 | V158^a^, **G160**^b^, G161^b^, **A163**^b^, **R183**^ab^, R188^ac^, I246^a^ | -7.68 | Q46^a^, T49^b^, T50^b^, T51^a^, I142^a^, E166^a^, H170^a^ |
| T-159 | -8.63 | V158^a^, **G160**^b^, G161^b^, **A163**^b^, **R183**^ab^, R188^c^, I246^a^ | -8.40 | Q46^a^, T49^b^, T50^b^, I142^a^, E166^a^ |
| T-161 | -7.81 | V158^a^, **G160**^b^, G161^b^, D162^b^, **A163**^ab^, **R183**^abc^, I246^a^ | -7.97 | Q46^a^, T49^b^, T50^b^, T51^a^, I142^a^, E166^a^, H170^a^ |
| T-163 | -7.25 | **G160**^b^, G161^b^, D162^a^, **H182**^d^, **R183**^d^, I246^a^, G247^b^ | -7.47 | Q46^a^, L47^a^, T50^b^, **A163**^a^, E166^a^ |
| T-164 | -6.98 | Q46^a^, T49^b^, **A163**^a^, H248^c^ | -7.16 | Q46^a^, L47^a^, T50^b^, **A163**^a^, E167^b^ |
| T-165 | -7.20 | Q46^a^, T49^b^, A121^a^, H248^c^ | -7.40 | Q46^a^, T49^b^, A121^a^, H248^c^ |
| T-166 | -8.34 | V158^a^, G161^b^, **R183**^abc^, I246^a^, H248^a^ | -7.84 | V41^a^_,_ W133^a^, Q134^a^, A139^ab^, **C140**^b^ |
| T-167 | -7.07 | **A163**^a^, **R183**^bc^, Ile246^a^ | -6.70 | T49^b^, T50^b^, T51^a^, E166^a^, E167^b^ |
| T-168 | -7.56 | V158^a^, **G160**^b^, **R183**^ac^, R188^b^, L211^b^, I246^a^ | -7.48 | I142^a^, **A163**^a^, E166^a^, L169^a^, H170^a^ |
| T-169 | -7.12 | V158^a^, **A163**^a^, **H182**^d^, **R183**^ad^, R188^a^, I246^a^ | -7.29 | T51^b^, I142^a^, **A163**^a^, E167^b^, L169^a^, H170^a^ |
| T-170 | -7.84 | V158^a^, **G160**^b^, G161^b^, **R183**^ab^, E210^a^, I246^a^ | -8.38 | T49^b^, T50^b^, T51^a^, I142^a^, E166^a^ |
| MNZ | -4.76 | **G160**^b^, G161^b^, **R291**^b^ |  |  |

Metronidazole (MNZ). Interactions: ^a^ hydrophobic, ^b^ H-bond, ^c^ π-cation, ^d^ salt bridge, ^e^ halogen
